# Supplementary material for: Visual experience has opposing influences on the quality of stimulus representation in adult primary visual cortex
Source: eLife. 2022 Nov 2;11:e80361. doi: 10.7554/eLife.80361 (PMC9629826; doi:10.7554/eLife.80361)
Supplement: Supplementary file 1. — (A) Fraction of responsive neurons: Data are normalized to the number of neurons segmented in a given session. The SEM was non-overlapping between Baseline 1 (B1) and post dark exposure (pDE), therefore one STD is included. The fraction of responsive neurons in B1 versus pDE was not significantly different (paired t-test [df: 5, t: 2.213, power: 0.433], p=0.078; n=6 animals). (B) Fraction of neurons included in Figures 1 and 2: Data are normalized to the number of neurons segmented in a given session. The SEM was overlapping in all cases. (C) Fraction of neurons included in Figure 3: Data are normalized to the number of neurons segmented in a given session. In Figure 3A and D, the SEM was non-overlapping between Baseline 2 (B2), pDE, and Rec, therefore one STD is included; the fraction of included neurons in B2 versus pDE and B2 versus Rec was not significantly different (paired t-test [df: 5, t: −2.458,–2.685, power: 0.509, 0.580], adjusted for two multiple comparisons, p=0.0574 and p=0.0871, respectively; n=6 animals). In Figure 3E, the SEM was non-overlapping between B1 and B2 (pDE-B2), therefore one STD is included; the fraction of included neurons in B1 versus B2 (pDE-B2) was not significantly different (paired t-test [df: 5, t: 2.50, power: 0.523], p=0.0545; n=6 animals). (D) Fraction of neurons included in Figure 1—figure supplement 1: Data are normalized to the number of neurons segmented in a given session. The SEM was overlapping in all cases. [file elife-80361-supp1.docx]

|  | Fraction of responsive neurons (ANOVA) | | | |
| --- | --- | --- | --- | --- |
|  | B1 | B2 | pDE* | Rec |
| mouse 1 | 0.365449 | 0.373188 | 0.319392 | 0.296748 |
| mouse 2 | 0.606299 | 0.615385 | 0.463115 | 0.514019 |
| mouse 3 | 0.603774 | 0.425837 | 0.387755 | 0.413043 |
| mouse 4 | 0.376404 | 0.399083 | 0.443709 | 0.455696 |
| mouse 5 | 0.404255 | 0.371179 | 0.351515 | 0.264901 |
| mouse 6 | 0.490741 | 0.448413 | 0.32973 | 0.44403 |
| Average | 0.47 | 0.44 | 0.38 | 0.40 |
| SEM | 0.05 | 0.04 | 0.02 | 0.04 |
| 1 STD | 0.11 | 0.09 | 0.06 | 0.10 |

Supplementary File 1A

| Figures 1 and 2 | Fraction of neurons included | | | | | |
| --- | --- | --- | --- | --- | --- | --- |
|  | Baseline | | DE | | LRx | |
|  | B1 | B2 | B2 | pDE | pDE | Rec |
| mouse 1 | 0.119601 | 0.130435 | 0.112319 | 0.117871 | 0.121673 | 0.130081 |
| mouse 2 | 0.23622 | 0.25641 | 0.260684 | 0.25 | 0.209016 | 0.238318 |
| mouse 3 | 0.183962 | 0.186603 | 0.205742 | 0.219388 | 0.19898 | 0.211957 |
| mouse 4 | 0.213483 | 0.174312 | 0.174312 | 0.251656 | 0.271523 | 0.259494 |
| mouse 5 | 0.153191 | 0.157205 | 0.100437 | 0.139394 | 0.109091 | 0.119205 |
| mouse 6 | 0.185185 | 0.15873 | 0.134921 | 0.183784 | 0.189189 | 0.130597 |
| Average | 0.18 | 0.18 | 0.16 | 0.19 | 0.18 | 0.18 |
| SEM | 0.02 | 0.02 | 0.03 | 0.02 | 0.02 | 0.03 |

Supplementary File 1B

| Figure 3A,D | Fraction of neurons included | | |  |  |  |  |  |
| --- | --- | --- | --- | --- | --- | --- | --- | --- |
|  | B2 | pDE* | Rec* |  |  |  |  |  |
| mouse 1 | 0.413043 | 0.43346 | 0.463415 |  |  |  |  |  |
| mouse 2 | 0.418803 | 0.401639 | 0.457944 |  |  |  |  |  |
| mouse 3 | 0.358852 | 0.382653 | 0.407609 |  |  |  |  |  |
| mouse 4 | 0.293578 | 0.423841 | 0.405063 |  |  |  |  |  |
| mouse 5 | 0.240175 | 0.333333 | 0.364238 |  |  |  |  |  |
| mouse 6 | 0.380952 | 0.518919 | 0.358209 |  |  |  |  |  |
| Average | 0.35 | 0.42 | 0.41 |  |  |  |  |  |
| SEM | 0.03 | 0.03 | 0.02 |  |  |  |  |  |
| 1 STD | 0.07 | 0.06 | 0.04 |  |  |  |  |  |
| Figure 3E | Fraction of neurons included | | | | | | | |
|  | B1-B2 | | pDE-B2 | | Rec-B2 | | pDE-Rec | |
|  | B1 | B2 | B2* | pDE | Rec | B2 | pDE | Rec |
| mouse 1 | 0.554817 | 0.605072 | 0.536232 | 0.562738 | 0.650407 | 0.57971 | 0.51711 | 0.552846 |
| mouse 2 | 0.53937 | 0.58547 | 0.534188 | 0.512295 | 0.672897 | 0.615385 | 0.483607 | 0.551402 |
| mouse 3 | 0.575472 | 0.583732 | 0.5311 | 0.566327 | 0.565217 | 0.497608 | 0.489796 | 0.521739 |
| mouse 4 | 0.646067 | 0.527523 | 0.422018 | 0.609272 | 0.588608 | 0.426606 | 0.516556 | 0.493671 |
| mouse 5 | 0.544681 | 0.558952 | 0.467249 | 0.648485 | 0.529801 | 0.349345 | 0.412121 | 0.450331 |
| mouse 6 | 0.615741 | 0.527778 | 0.456349 | 0.621622 | 0.537313 | 0.571429 | 0.67027 | 0.462687 |
| Average | 0.58 | 0.56 | 0.49 | 0.59 | 0.59 | 0.51 | 0.51 | 0.51 |
| SEM | 0.02 | 0.01 | 0.02 | 0.02 | 0.02 | 0.04 | 0.03 | 0.02 |
| 1 STD | 0.04 | 0.03 | 0.05 | 0.05 | 0.06 | 0.10 | 0.09 | 0.04 |
| Figure 3F | Fraction of neurons included | | |  |  |  |  |  |
|  | B2 | pDE | Rec |  |  |  |  |  |
| mouse 1 | 0.181159 | 0.144487 | 0.186992 |  |  |  |  |  |
| mouse 2 | 0.316239 | 0.262295 | 0.308411 |  |  |  |  |  |
| mouse 3 | 0.229665 | 0.229592 | 0.23913 |  |  |  |  |  |
| mouse 4 | 0.197248 | 0.278146 | 0.28481 |  |  |  |  |  |
| mouse 5 | 0.113537 | 0.151515 | 0.178808 |  |  |  |  |  |
| mouse 6 | 0.190476 | 0.210811 | 0.201493 |  |  |  |  |  |
| Average | 0.20 | 0.21 | 0.23 |  |  |  |  |  |
| SEM | 0.03 | 0.02 | 0.02 |  |  |  |  |  |
| Figure 3G | Fraction of neurons included | | |  |  |  |  |  |
|  | B2 | pDE | Rec |  |  |  |  |  |
| mouse 1 | 0.333333 | 0.254753 | 0.264228 |  |  |  |  |  |
| mouse 2 | 0.559829 | 0.446721 | 0.490654 |  |  |  |  |  |
| mouse 3 | 0.373206 | 0.372449 | 0.391304 |  |  |  |  |  |
| mouse 4 | 0.334862 | 0.417219 | 0.443038 |  |  |  |  |  |
| mouse 5 | 0.323144 | 0.30303 | 0.238411 |  |  |  |  |  |
| mouse 6 | 0.333333 | 0.308108 | 0.380597 |  |  |  |  |  |
| Average | 0.38 | 0.35 | 0.37 |  |  |  |  |  |
| SEM | 0.04 | 0.03 | 0.04 |  |  |  |  |  |

Supplementary File 1C

| Figure 1-figure supplement 1B | Fraction of neurons included | | |  | | |
| --- | --- | --- | --- | --- | --- | --- |
|  | B1 | B2 | pDE |  |  |  |
| mouse 1 | 0.325581 | 0.333333 | 0.254753 |  |  |  |
| mouse 2 | 0.551181 | 0.559829 | 0.446721 |  |  |  |
| mouse 3 | 0.556604 | 0.373206 | 0.372449 |  |  |  |
| mouse 4 | 0.342697 | 0.334862 | 0.417219 |  |  |  |
| mouse 5 | 0.361702 | 0.323144 | 0.30303 |  |  |  |
| mouse 6 | 0.458333 | 0.333333 | 0.308108 |  |  |  |
| Average | 0.43 | 0.38 | 0.35 |  |  |  |
| SEM | 0.04 | 0.04 | 0.03 |  |  |  |
| Figure 1-figure supplement 1C | Fraction of neurons included | | | |  | |
|  | Baseline | | DE | |  |  |
|  | B1 | B2 | B2 | pDE |  |  |
| mouse 1 | 0.10299 | 0.112319 | 0.086957 | 0.091255 |  |  |
| mouse 2 | 0.224409 | 0.24359 | 0.222222 | 0.213115 |  |  |
| mouse 3 | 0.165094 | 0.167464 | 0.196172 | 0.209184 |  |  |
| mouse 4 | 0.202247 | 0.165138 | 0.165138 | 0.238411 |  |  |
| mouse 5 | 0.140426 | 0.144105 | 0.09607 | 0.133333 |  |  |
| mouse 6 | 0.157407 | 0.134921 | 0.119048 | 0.162162 |  |  |
| Average | 0.17 | 0.16 | 0.15 | 0.17 |  |  |
| SEM | 0.02 | 0.02 | 0.02 | 0.02 |  |  |
| Figure 1-figure supplement 1D | Fraction of neurons included | | | | | |
|  | Baseline | | DE | | LRx | |
|  | B1 | B2 | B2 | pDE | pDE | Rec |
| mouse 1 | 0.13289 | 0.144928 | 0.141304 | 0.148289 | 0.136882 | 0.146341 |
| mouse 2 | 0.26378 | 0.286325 | 0.260684 | 0.25 | 0.213115 | 0.242991 |
| mouse 3 | 0.193396 | 0.196172 | 0.215311 | 0.229592 | 0.19898 | 0.211957 |
| mouse 4 | 0.219101 | 0.178899 | 0.192661 | 0.278146 | 0.271523 | 0.259494 |
| mouse 5 | 0.157447 | 0.161572 | 0.10917 | 0.151515 | 0.109091 | 0.119205 |
| mouse 6 | 0.217593 | 0.186508 | 0.134921 | 0.183784 | 0.194595 | 0.134328 |
| Average | 0.20 | 0.19 | 0.18 | 0.21 | 0.19 | 0.19 |
| SEM | 0.02 | 0.02 | 0.02 | 0.02 | 0.02 | 0.02 |

Supplementary File 1D
