# Supplementary figures and images for: Visual experience has opposing influences on the quality of stimulus representation in adult primary visual cortex (part 2 of 2)
Source: eLife. 2022 Nov 2;11:e80361. doi: 10.7554/eLife.80361 (PMC9629826; doi:10.7554/eLife.80361)

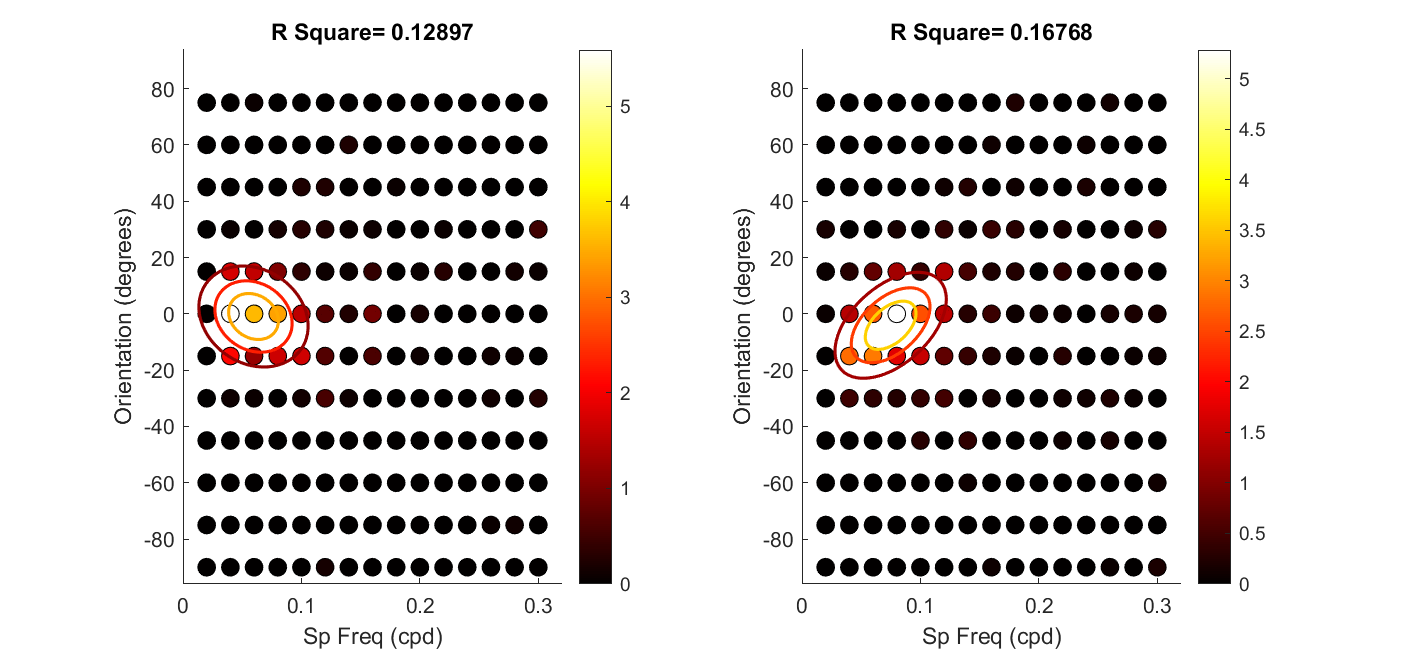

Supplement: Source data 1. — Tuning curves for all neurons scored as significantly tuned and tracked, for each of the three conditions: control, dark exposure (DE), and light reintroduction (LRx). [file elife-80361-data1.zip › SourceData1/b1_b2/2454_1R_cellPairID_13.png]

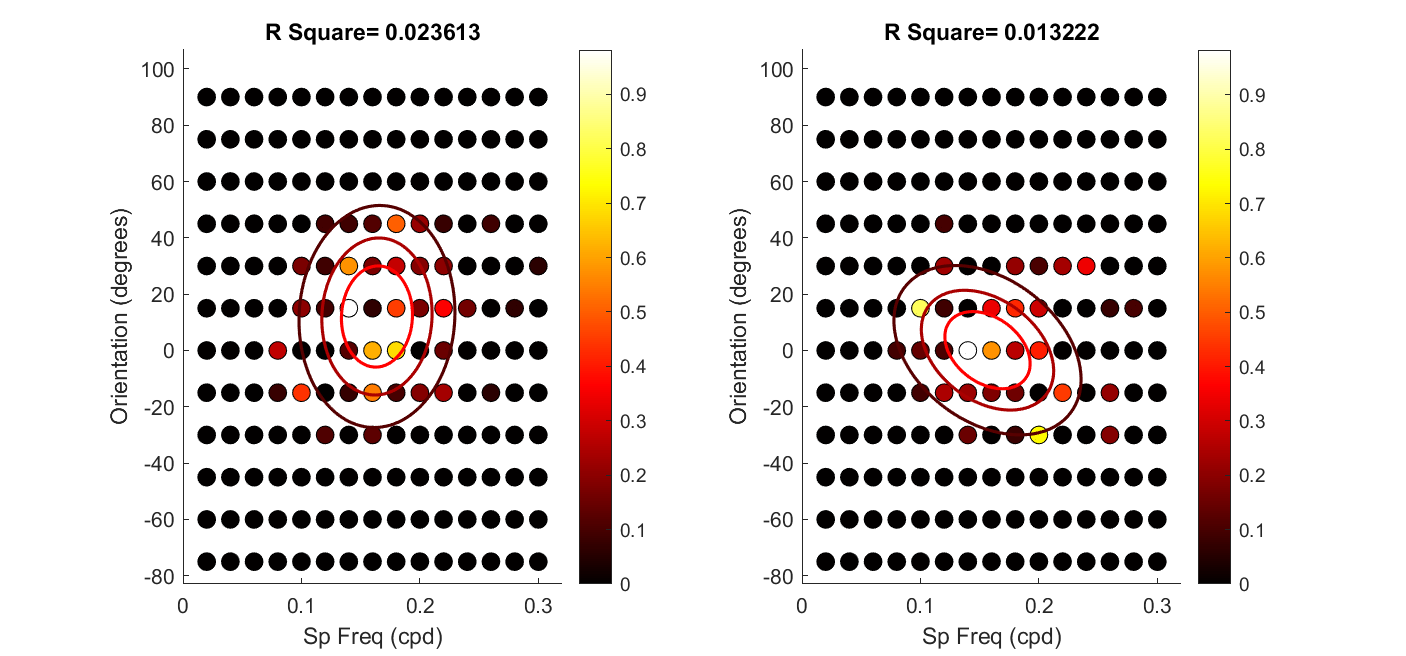

Supplement: Source data 1. — Tuning curves for all neurons scored as significantly tuned and tracked, for each of the three conditions: control, dark exposure (DE), and light reintroduction (LRx). [file elife-80361-data1.zip › SourceData1/b1_b2/2454_1R_cellPairID_14.png]

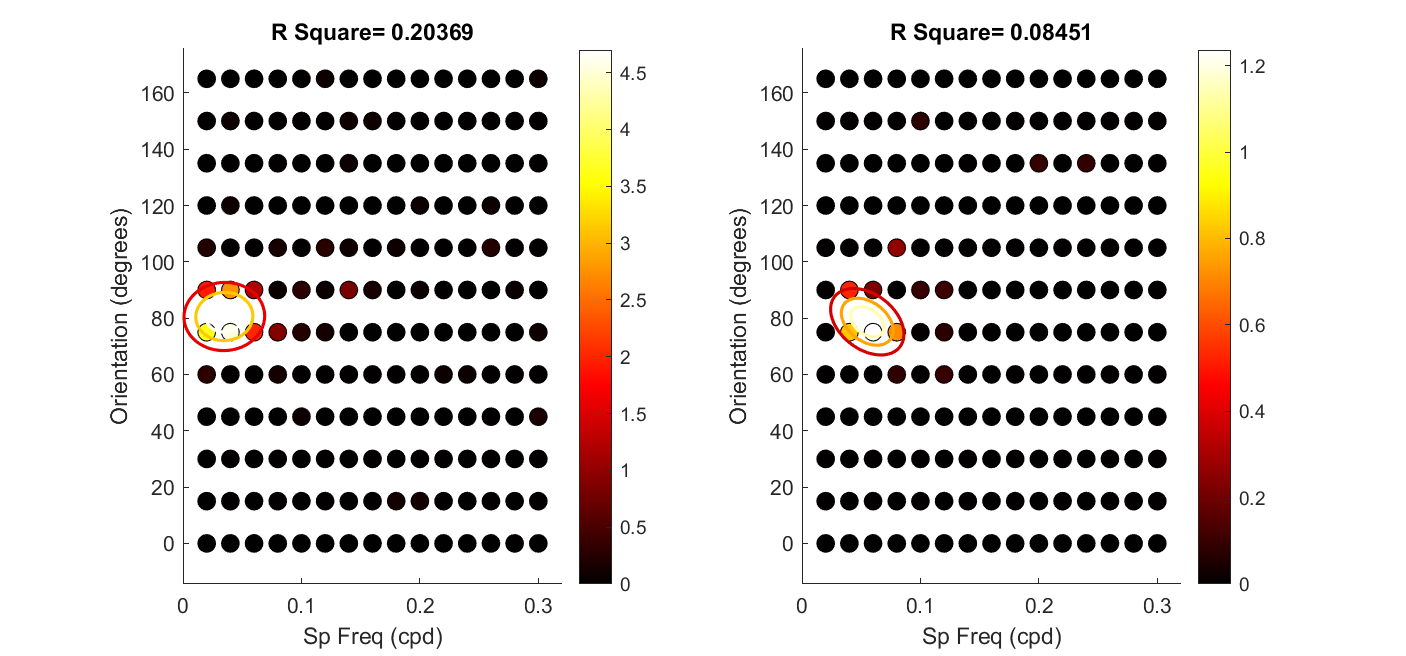

Supplement: Source data 1. — Tuning curves for all neurons scored as significantly tuned and tracked, for each of the three conditions: control, dark exposure (DE), and light reintroduction (LRx). [file elife-80361-data1.zip › SourceData1/b1_b2/2454_1R_cellPairID_15.png]

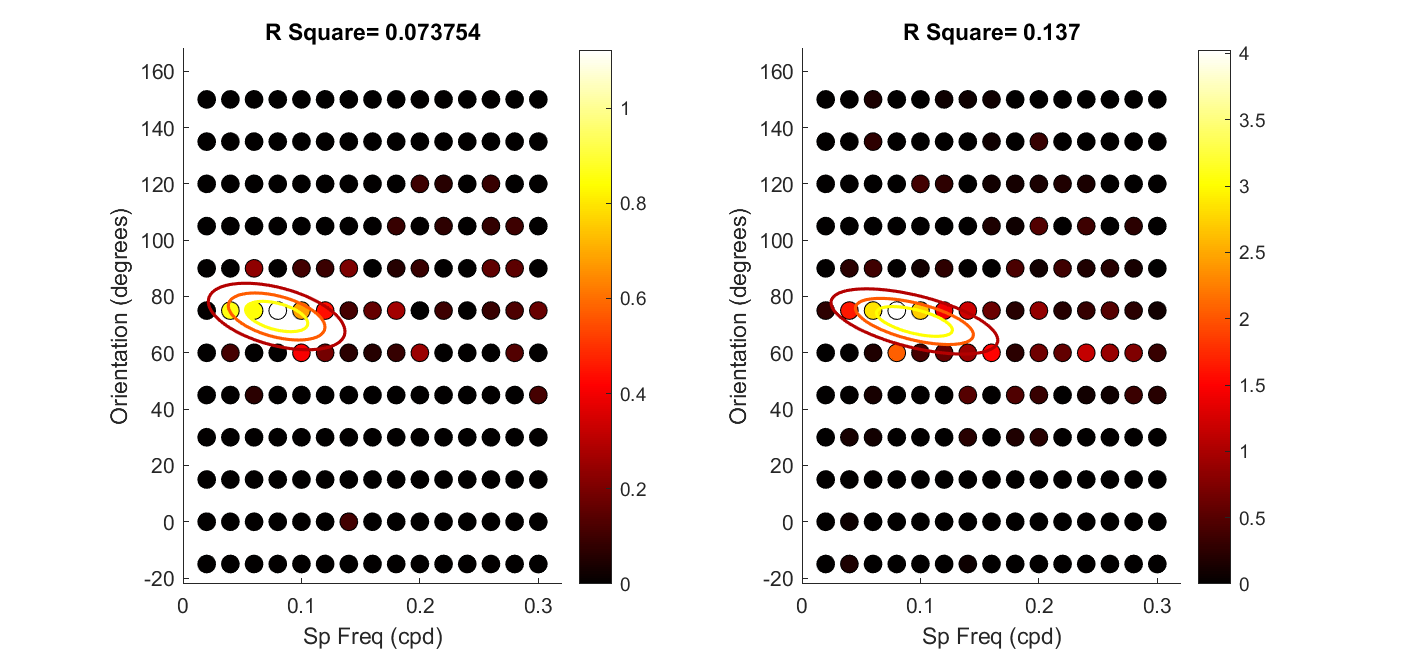

Supplement: Source data 1. — Tuning curves for all neurons scored as significantly tuned and tracked, for each of the three conditions: control, dark exposure (DE), and light reintroduction (LRx). [file elife-80361-data1.zip › SourceData1/b1_b2/2454_1R_cellPairID_16.png]

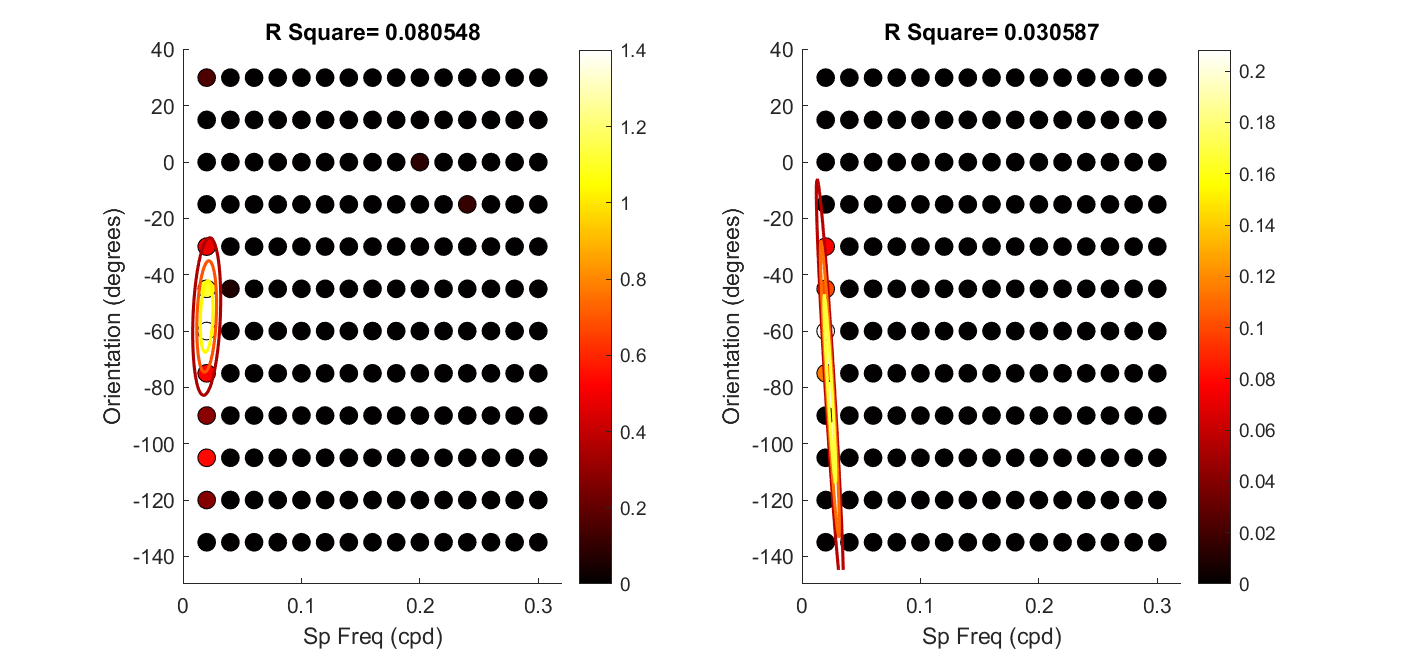

Supplement: Source data 1. — Tuning curves for all neurons scored as significantly tuned and tracked, for each of the three conditions: control, dark exposure (DE), and light reintroduction (LRx). [file elife-80361-data1.zip › SourceData1/b1_b2/2454_1R_cellPairID_17.png]

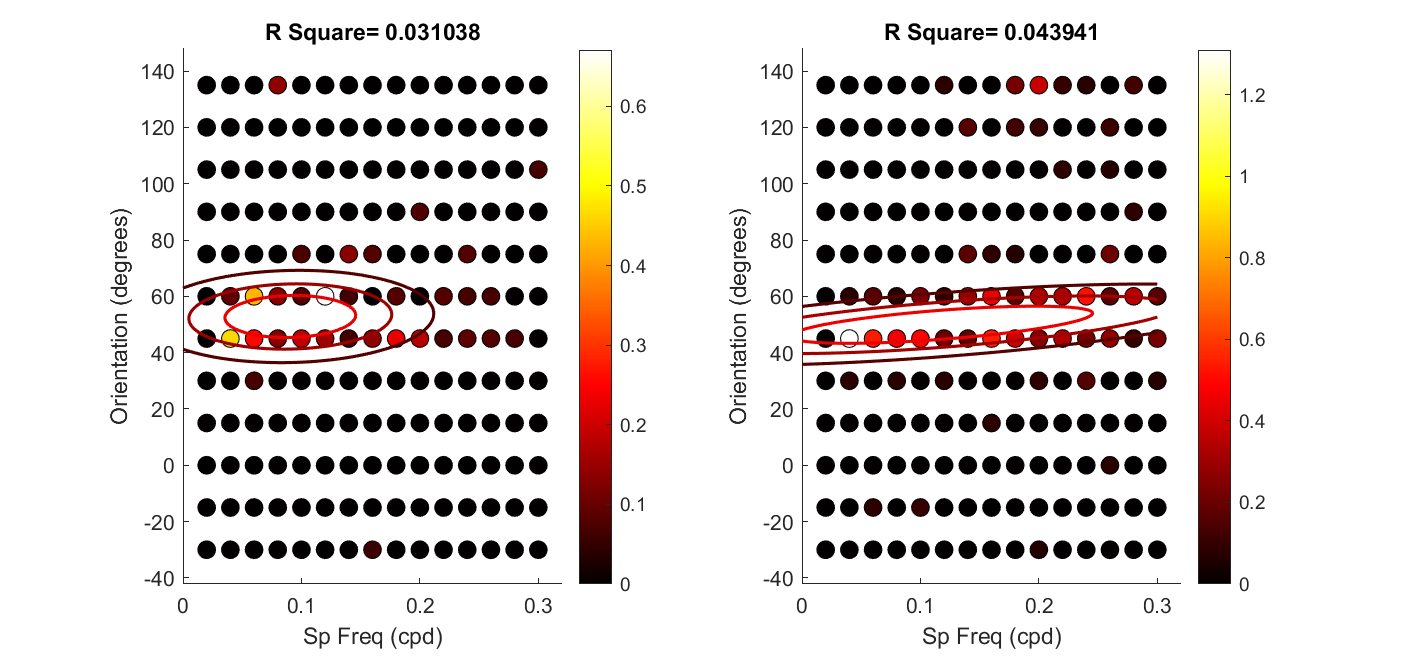

Supplement: Source data 1. — Tuning curves for all neurons scored as significantly tuned and tracked, for each of the three conditions: control, dark exposure (DE), and light reintroduction (LRx). [file elife-80361-data1.zip › SourceData1/b1_b2/2454_1R_cellPairID_18.png]

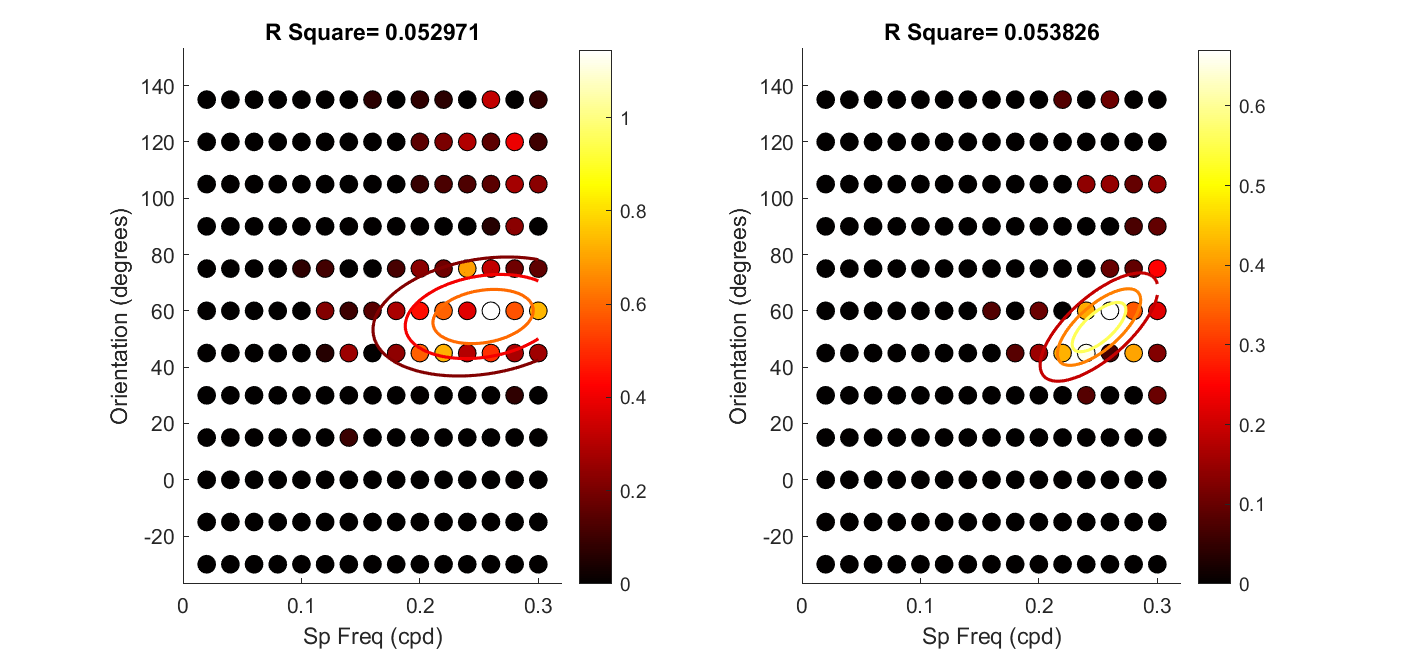

Supplement: Source data 1. — Tuning curves for all neurons scored as significantly tuned and tracked, for each of the three conditions: control, dark exposure (DE), and light reintroduction (LRx). [file elife-80361-data1.zip › SourceData1/b1_b2/2454_1R_cellPairID_19.png]

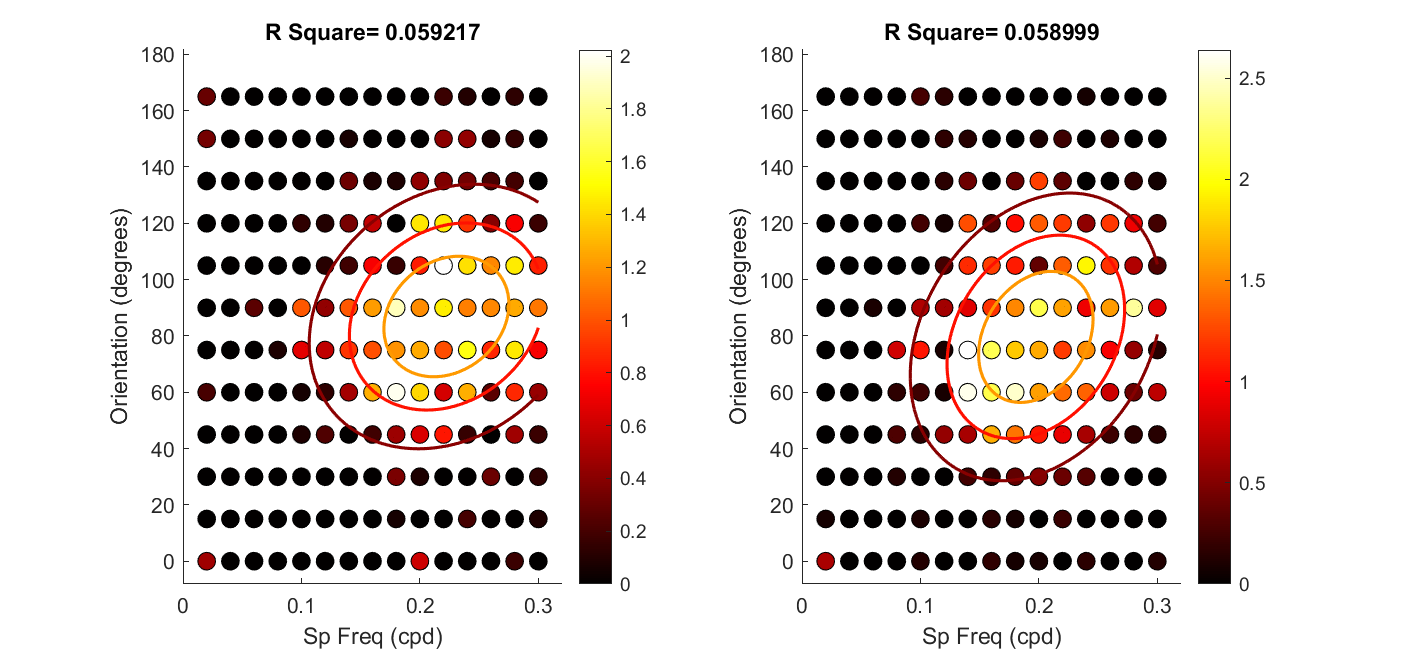

Supplement: Source data 1. — Tuning curves for all neurons scored as significantly tuned and tracked, for each of the three conditions: control, dark exposure (DE), and light reintroduction (LRx). [file elife-80361-data1.zip › SourceData1/b1_b2/2454_1R_cellPairID_2.png]

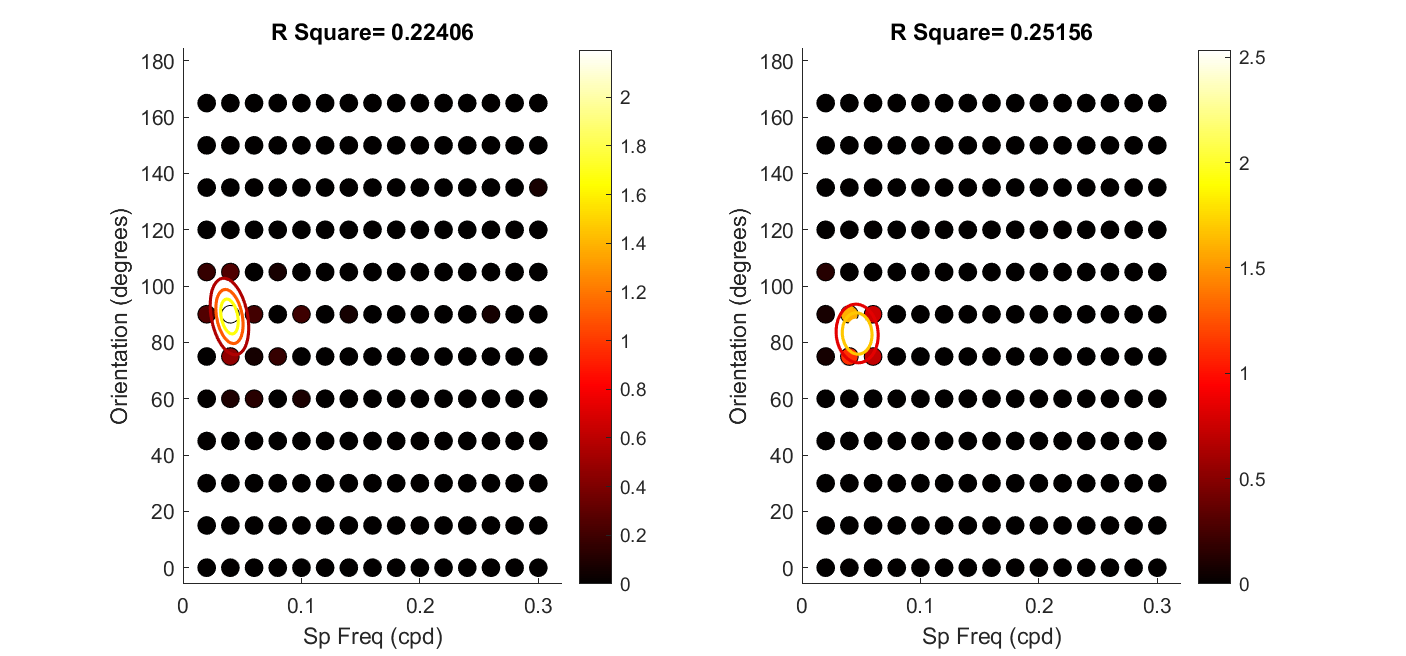

Supplement: Source data 1. — Tuning curves for all neurons scored as significantly tuned and tracked, for each of the three conditions: control, dark exposure (DE), and light reintroduction (LRx). [file elife-80361-data1.zip › SourceData1/b1_b2/2454_1R_cellPairID_20.png]

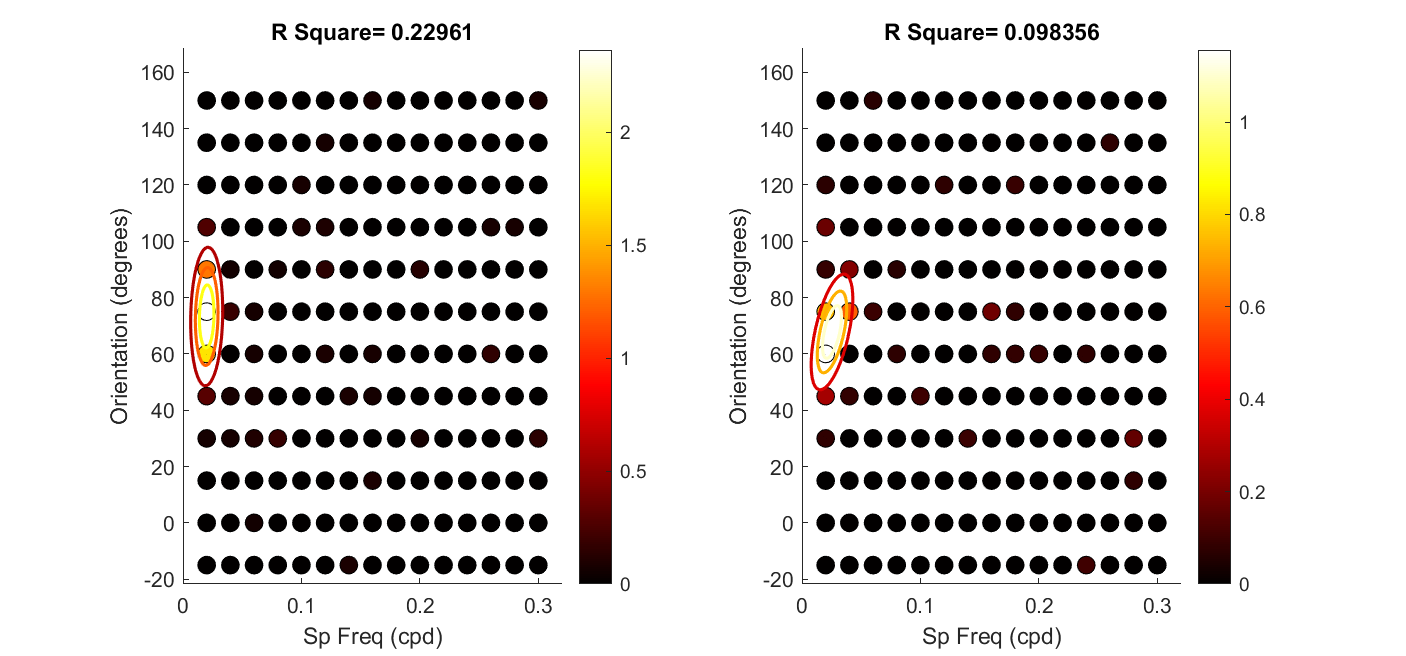

Supplement: Source data 1. — Tuning curves for all neurons scored as significantly tuned and tracked, for each of the three conditions: control, dark exposure (DE), and light reintroduction (LRx). [file elife-80361-data1.zip › SourceData1/b1_b2/2454_1R_cellPairID_21.png]

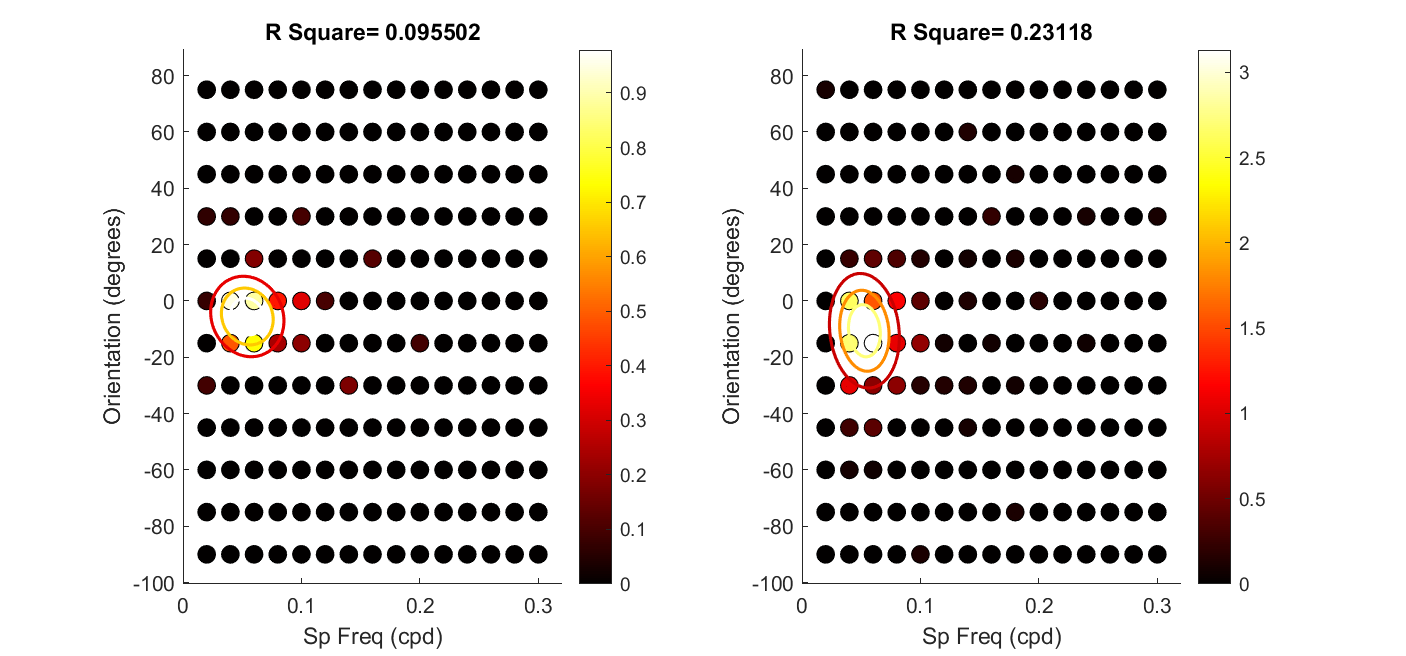

Supplement: Source data 1. — Tuning curves for all neurons scored as significantly tuned and tracked, for each of the three conditions: control, dark exposure (DE), and light reintroduction (LRx). [file elife-80361-data1.zip › SourceData1/b1_b2/2454_1R_cellPairID_22.png]

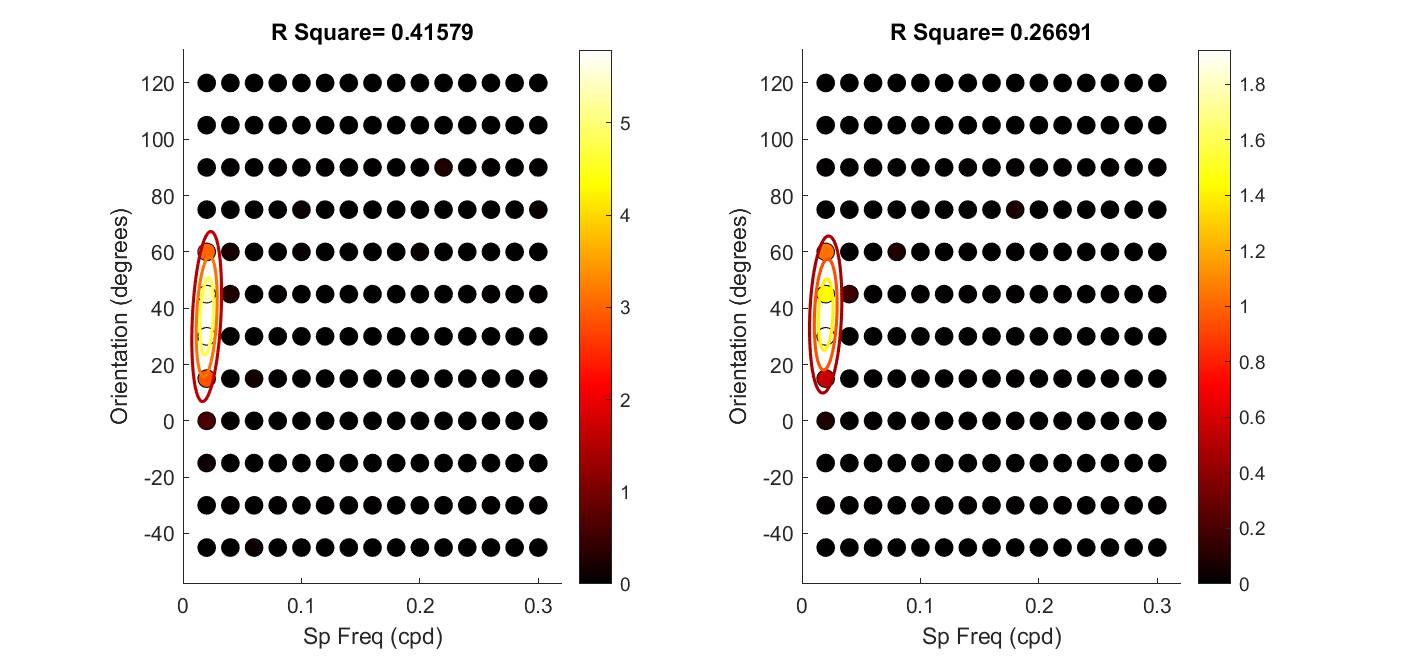

Supplement: Source data 1. — Tuning curves for all neurons scored as significantly tuned and tracked, for each of the three conditions: control, dark exposure (DE), and light reintroduction (LRx). [file elife-80361-data1.zip › SourceData1/b1_b2/2454_1R_cellPairID_23.png]

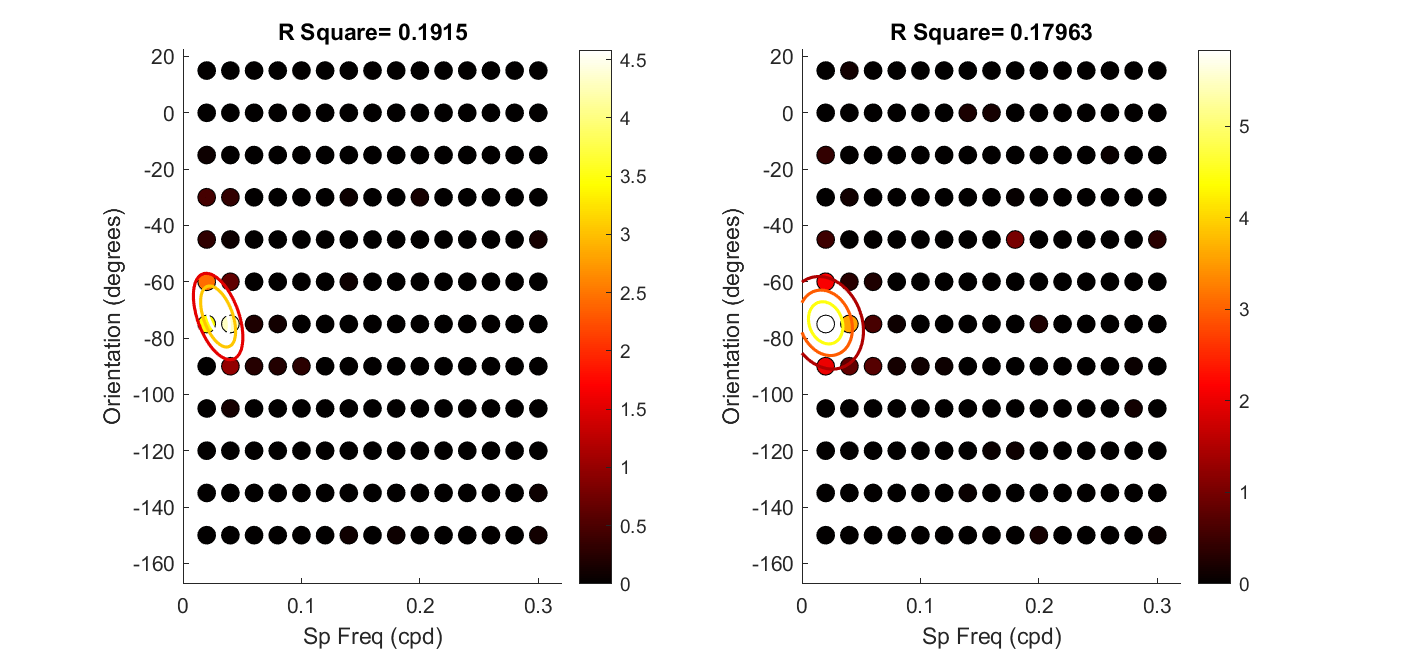

Supplement: Source data 1. — Tuning curves for all neurons scored as significantly tuned and tracked, for each of the three conditions: control, dark exposure (DE), and light reintroduction (LRx). [file elife-80361-data1.zip › SourceData1/b1_b2/2454_1R_cellPairID_24.png]

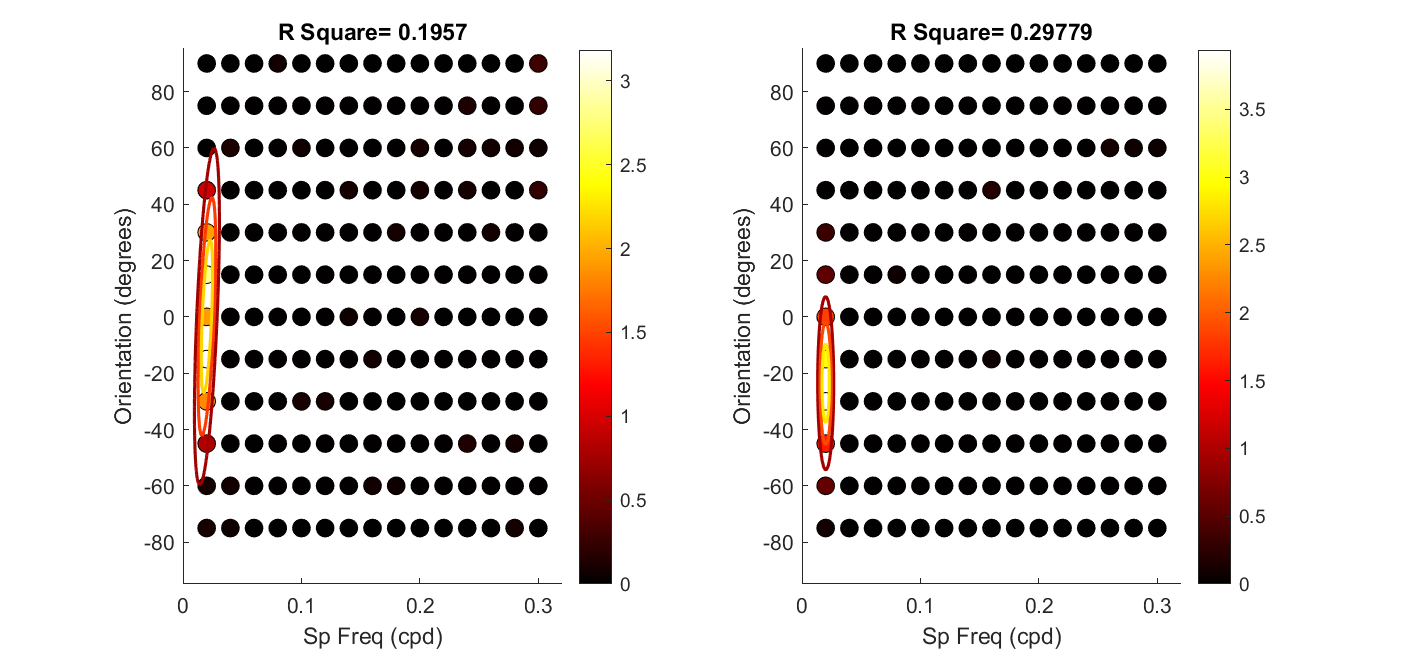

Supplement: Source data 1. — Tuning curves for all neurons scored as significantly tuned and tracked, for each of the three conditions: control, dark exposure (DE), and light reintroduction (LRx). [file elife-80361-data1.zip › SourceData1/b1_b2/2454_1R_cellPairID_25.png]

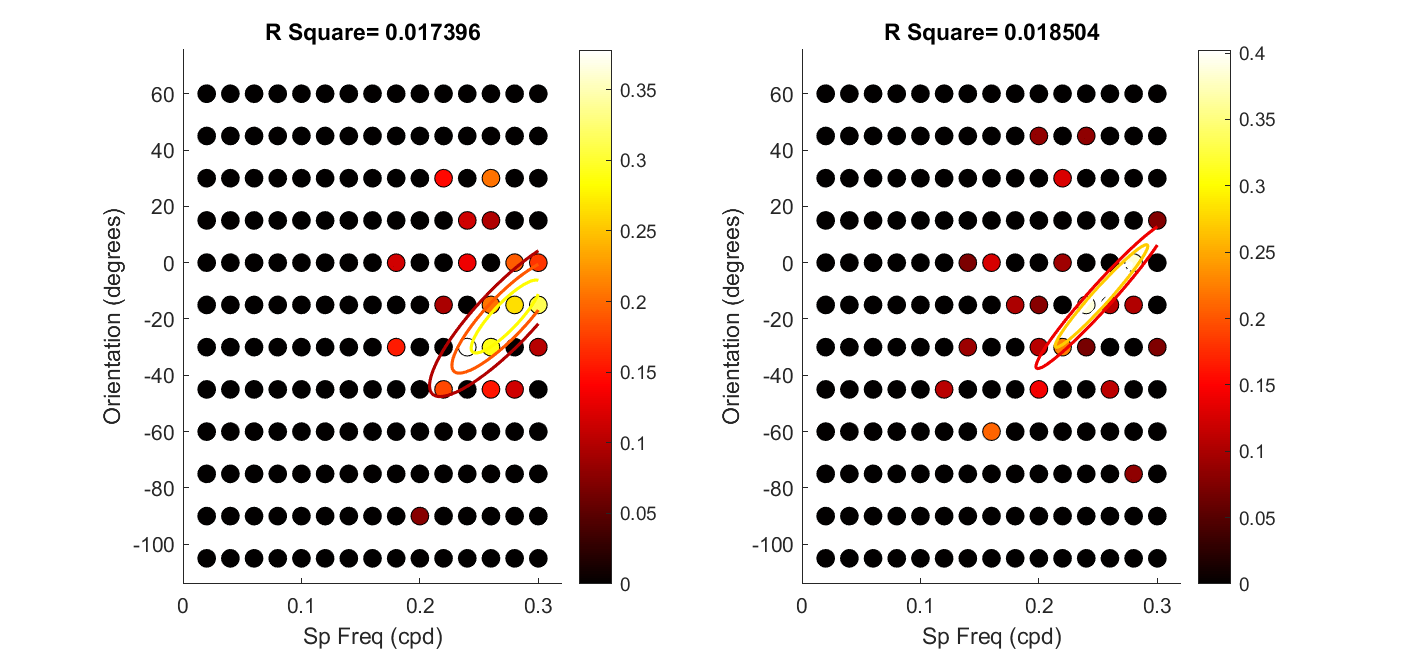

Supplement: Source data 1. — Tuning curves for all neurons scored as significantly tuned and tracked, for each of the three conditions: control, dark exposure (DE), and light reintroduction (LRx). [file elife-80361-data1.zip › SourceData1/b1_b2/2454_1R_cellPairID_26.png]

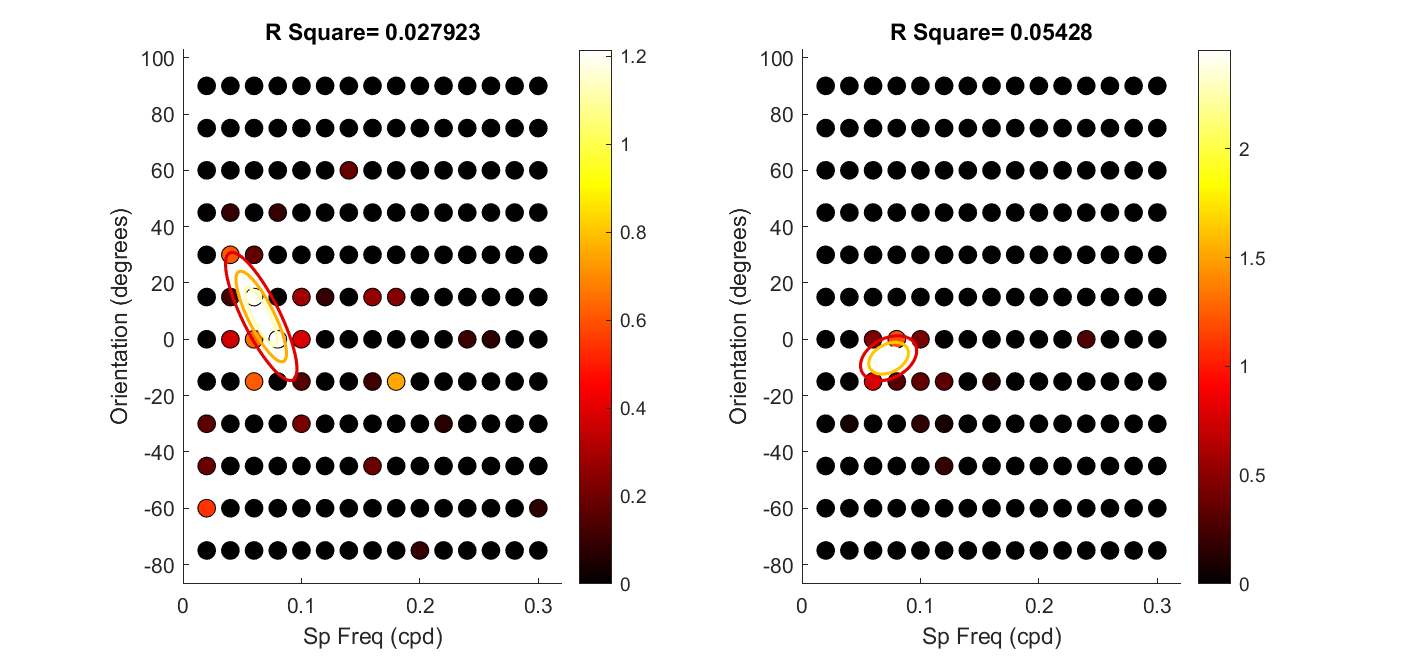

Supplement: Source data 1. — Tuning curves for all neurons scored as significantly tuned and tracked, for each of the three conditions: control, dark exposure (DE), and light reintroduction (LRx). [file elife-80361-data1.zip › SourceData1/b1_b2/2454_1R_cellPairID_27.png]

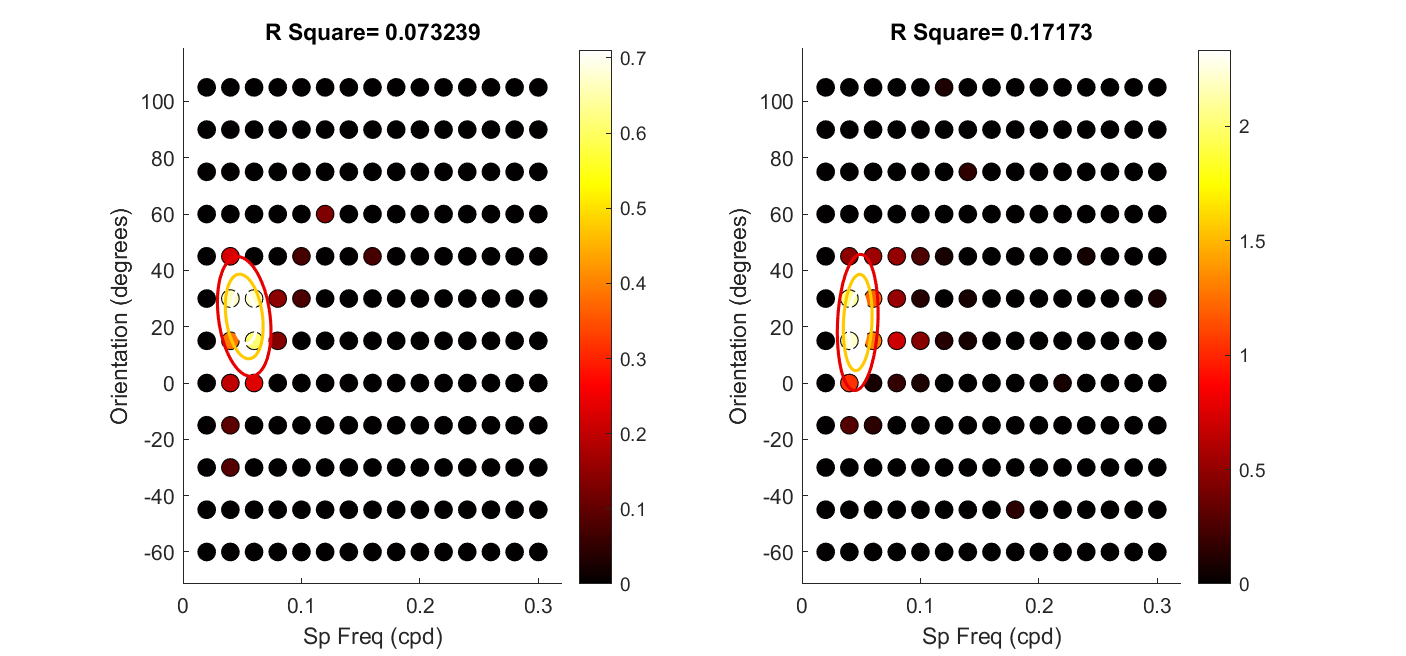

Supplement: Source data 1. — Tuning curves for all neurons scored as significantly tuned and tracked, for each of the three conditions: control, dark exposure (DE), and light reintroduction (LRx). [file elife-80361-data1.zip › SourceData1/b1_b2/2454_1R_cellPairID_28.png]

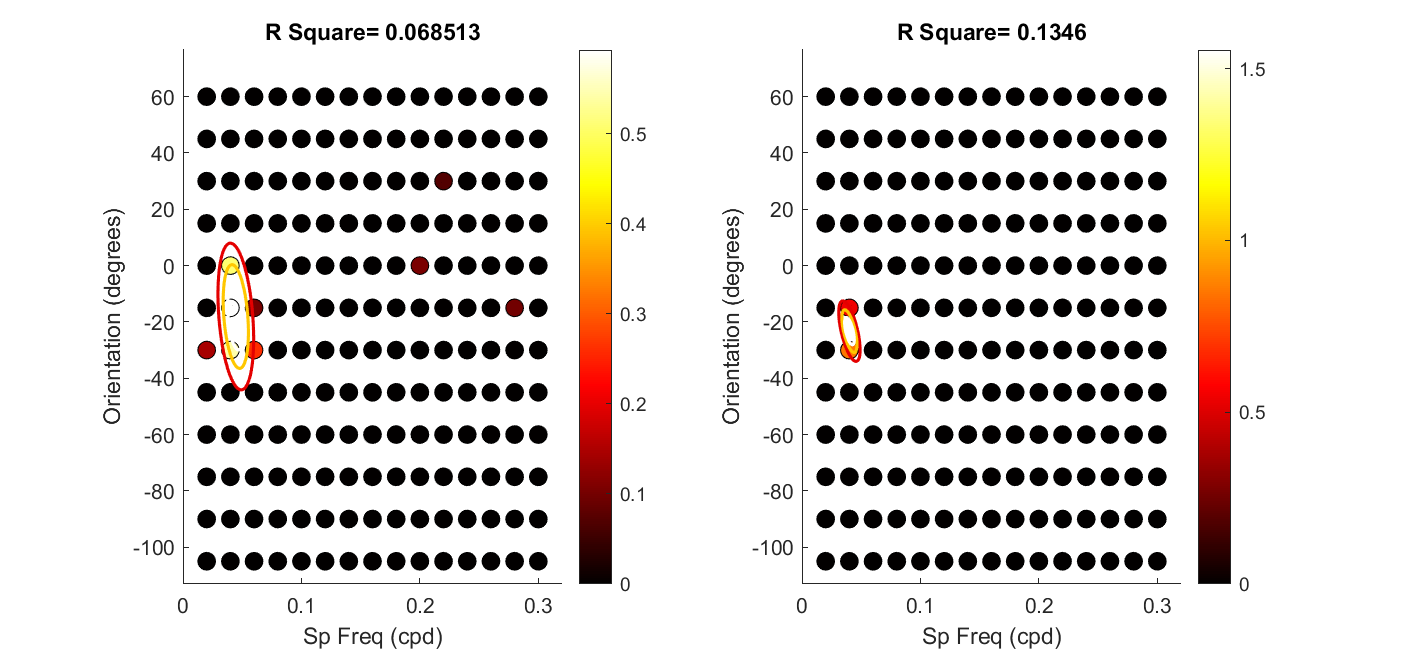

Supplement: Source data 1. — Tuning curves for all neurons scored as significantly tuned and tracked, for each of the three conditions: control, dark exposure (DE), and light reintroduction (LRx). [file elife-80361-data1.zip › SourceData1/b1_b2/2454_1R_cellPairID_29.png]

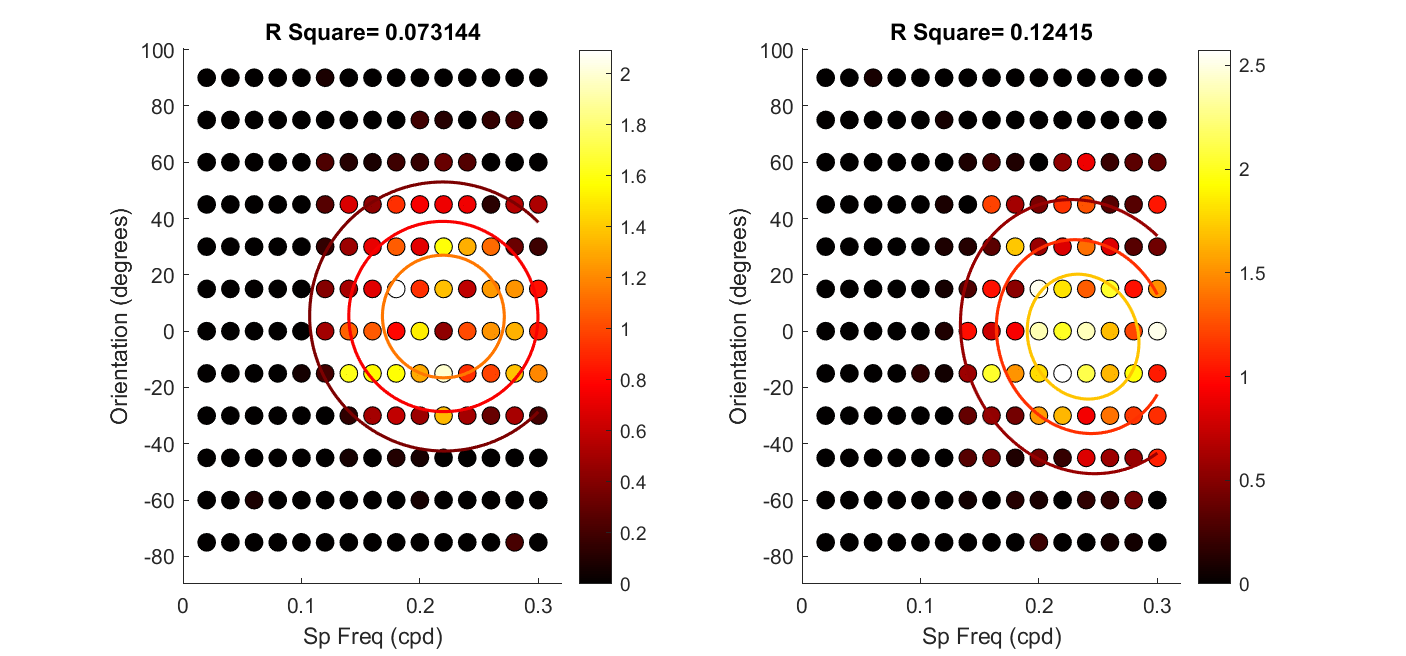

Supplement: Source data 1. — Tuning curves for all neurons scored as significantly tuned and tracked, for each of the three conditions: control, dark exposure (DE), and light reintroduction (LRx). [file elife-80361-data1.zip › SourceData1/b1_b2/2454_1R_cellPairID_3.png]

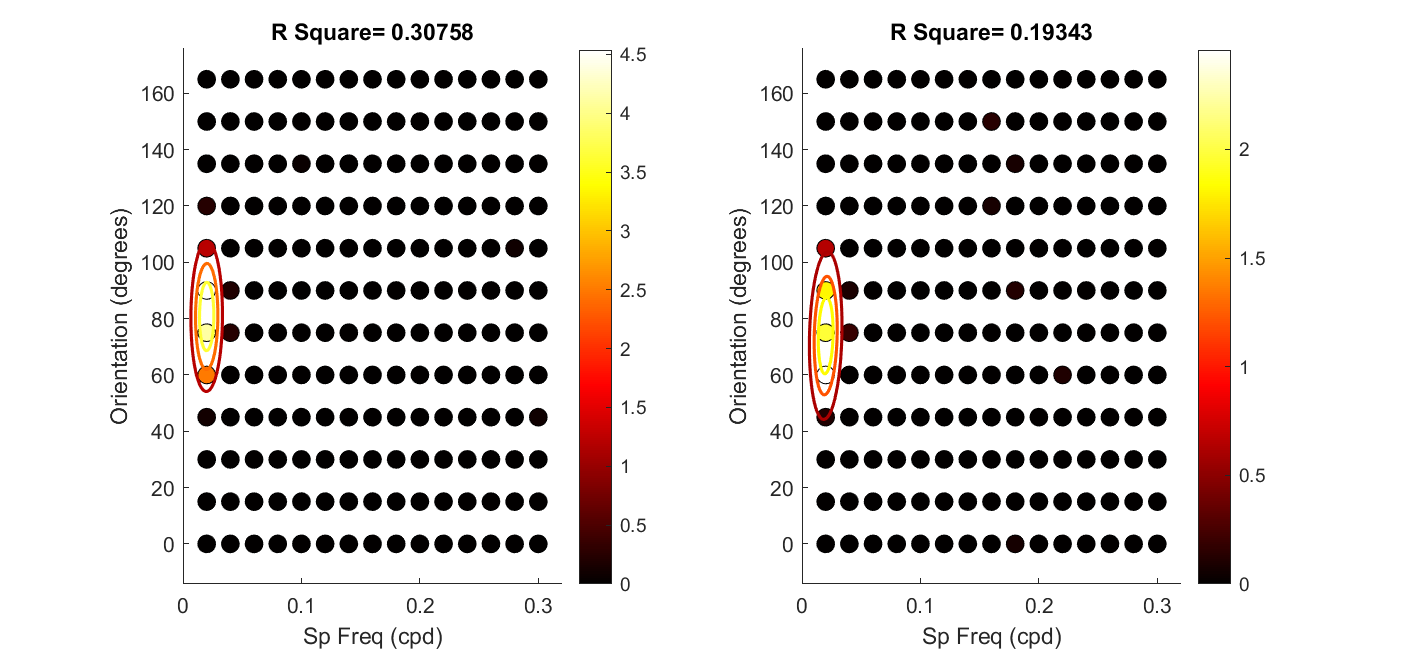

Supplement: Source data 1. — Tuning curves for all neurons scored as significantly tuned and tracked, for each of the three conditions: control, dark exposure (DE), and light reintroduction (LRx). [file elife-80361-data1.zip › SourceData1/b1_b2/2454_1R_cellPairID_30.png]

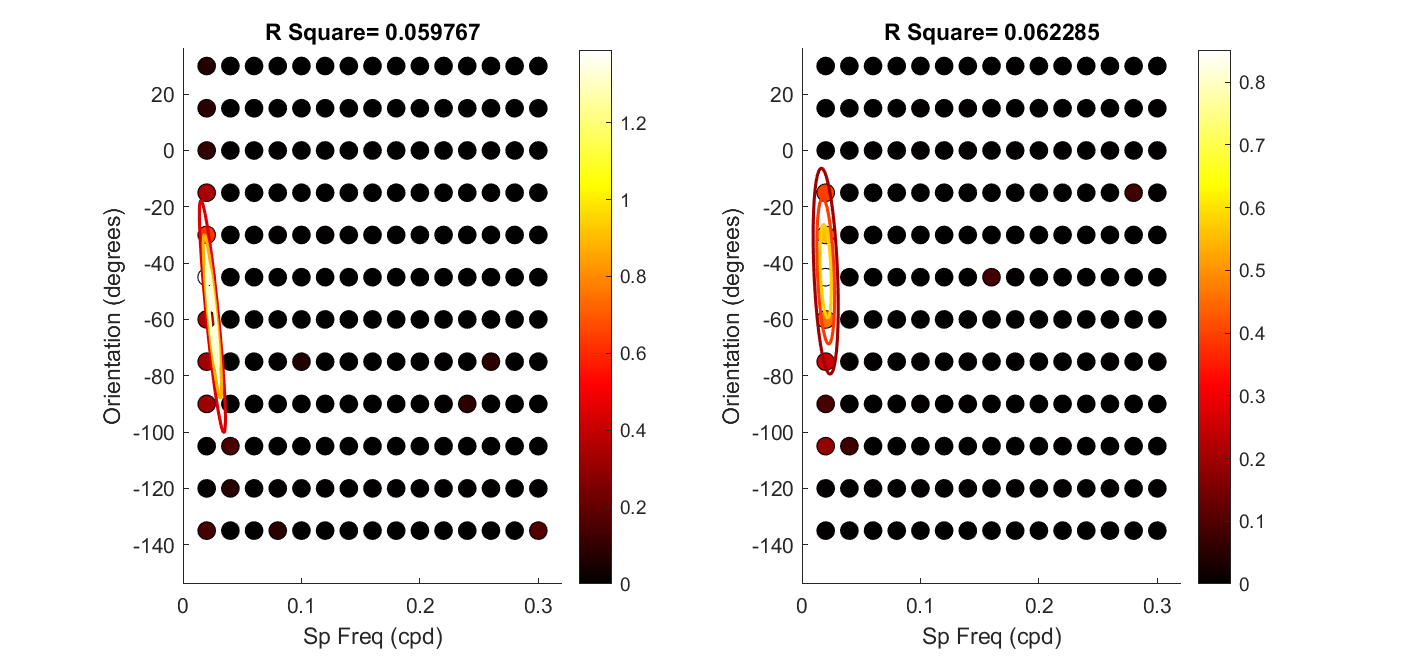

Supplement: Source data 1. — Tuning curves for all neurons scored as significantly tuned and tracked, for each of the three conditions: control, dark exposure (DE), and light reintroduction (LRx). [file elife-80361-data1.zip › SourceData1/b1_b2/2454_1R_cellPairID_31.png]

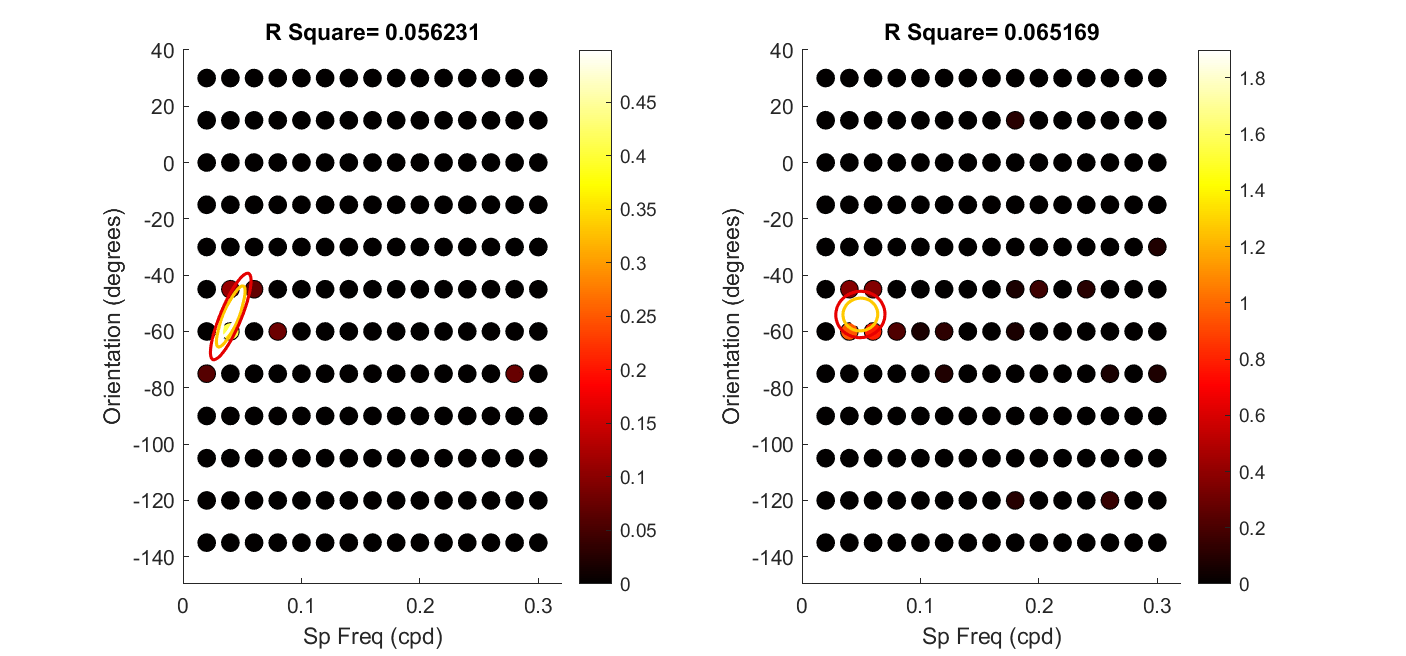

Supplement: Source data 1. — Tuning curves for all neurons scored as significantly tuned and tracked, for each of the three conditions: control, dark exposure (DE), and light reintroduction (LRx). [file elife-80361-data1.zip › SourceData1/b1_b2/2454_1R_cellPairID_32.png]

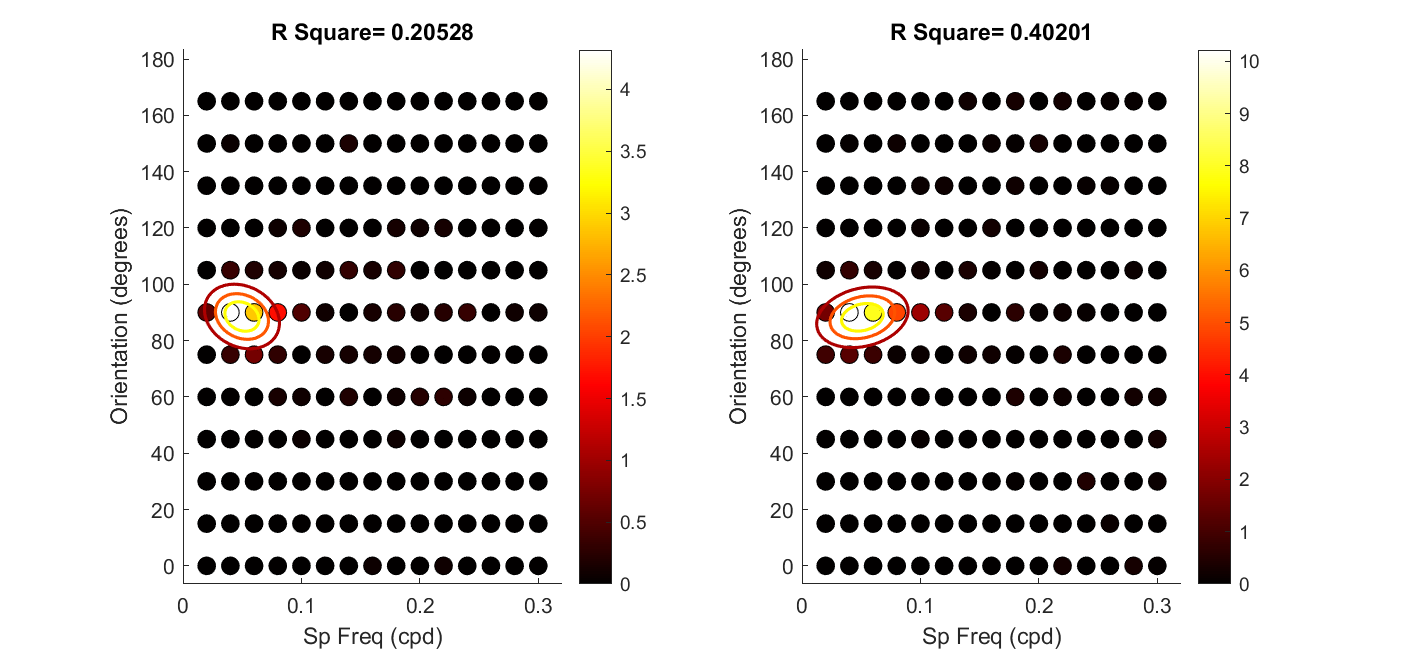

Supplement: Source data 1. — Tuning curves for all neurons scored as significantly tuned and tracked, for each of the three conditions: control, dark exposure (DE), and light reintroduction (LRx). [file elife-80361-data1.zip › SourceData1/b1_b2/2454_1R_cellPairID_33.png]

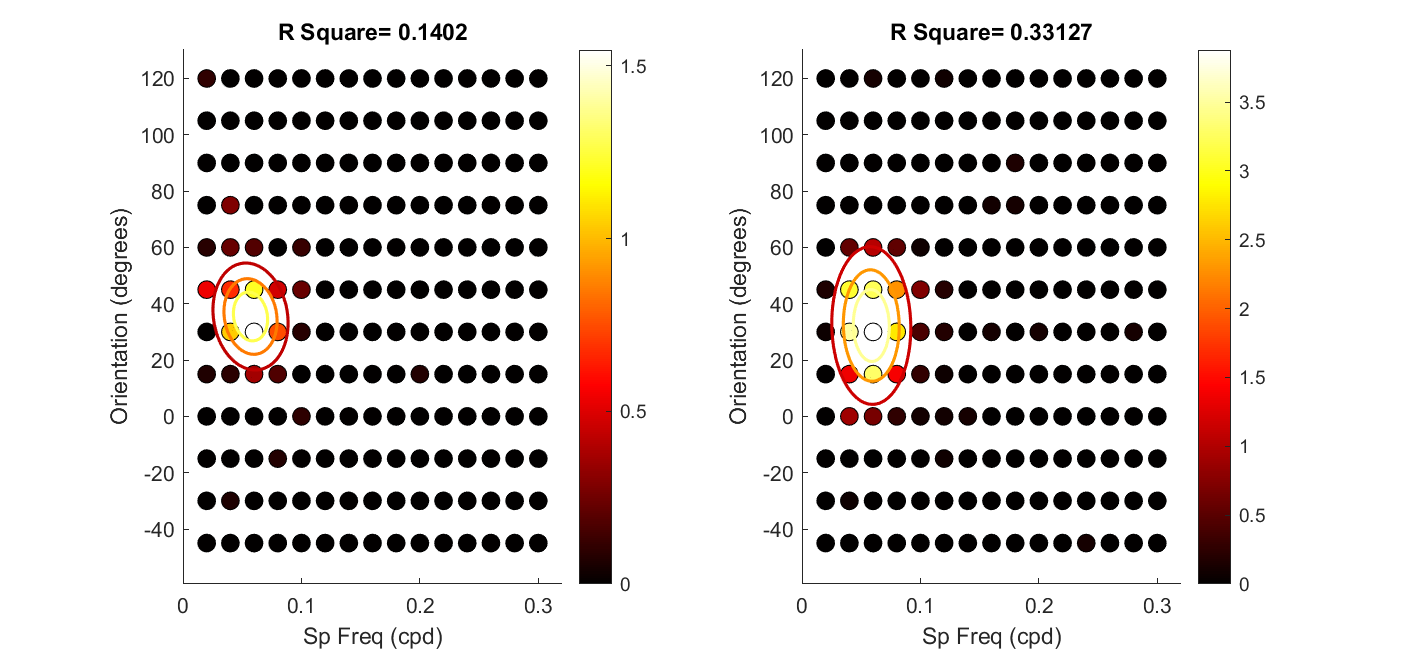

Supplement: Source data 1. — Tuning curves for all neurons scored as significantly tuned and tracked, for each of the three conditions: control, dark exposure (DE), and light reintroduction (LRx). [file elife-80361-data1.zip › SourceData1/b1_b2/2454_1R_cellPairID_34.png]

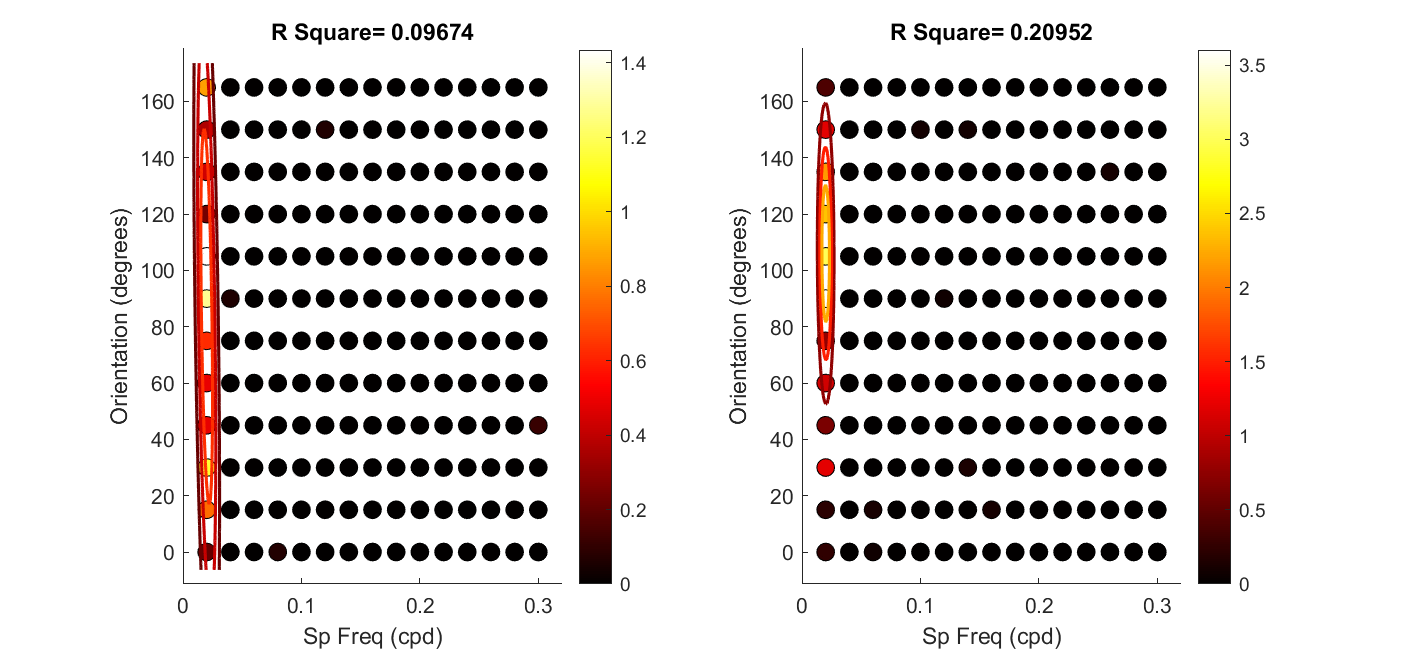

Supplement: Source data 1. — Tuning curves for all neurons scored as significantly tuned and tracked, for each of the three conditions: control, dark exposure (DE), and light reintroduction (LRx). [file elife-80361-data1.zip › SourceData1/b1_b2/2454_1R_cellPairID_35.png]

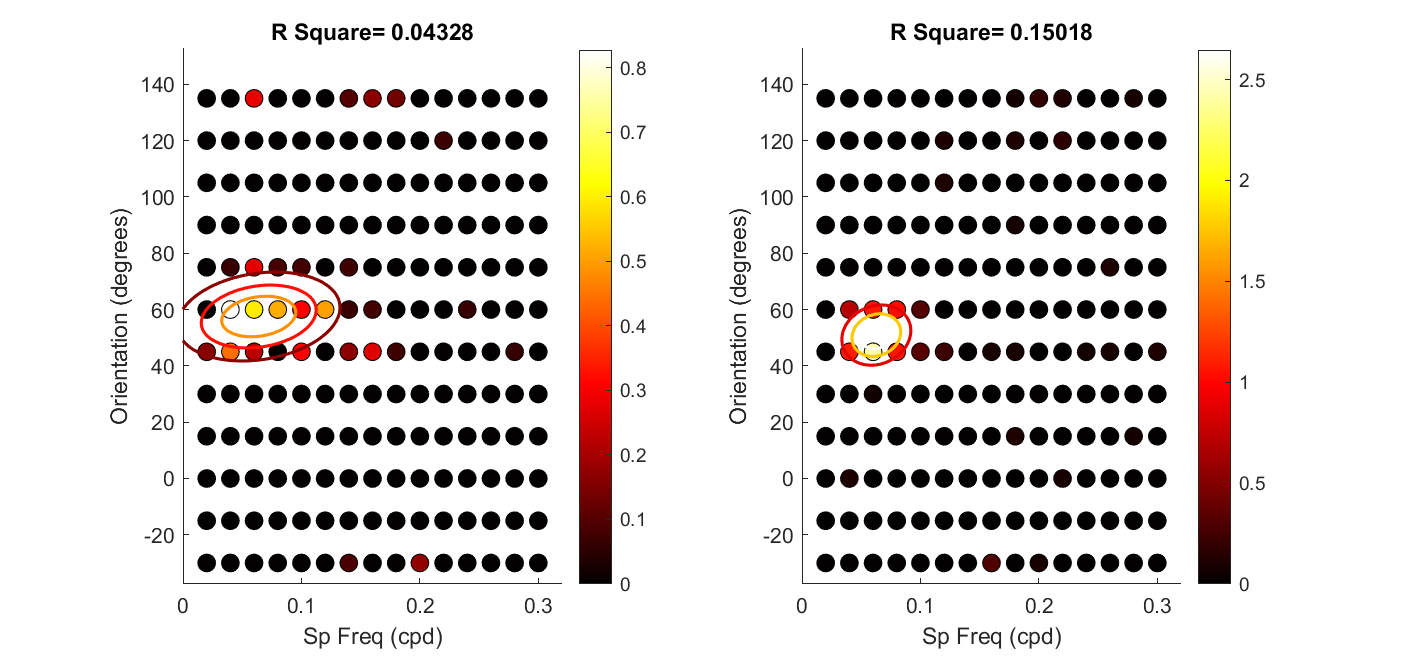

Supplement: Source data 1. — Tuning curves for all neurons scored as significantly tuned and tracked, for each of the three conditions: control, dark exposure (DE), and light reintroduction (LRx). [file elife-80361-data1.zip › SourceData1/b1_b2/2454_1R_cellPairID_36.png]

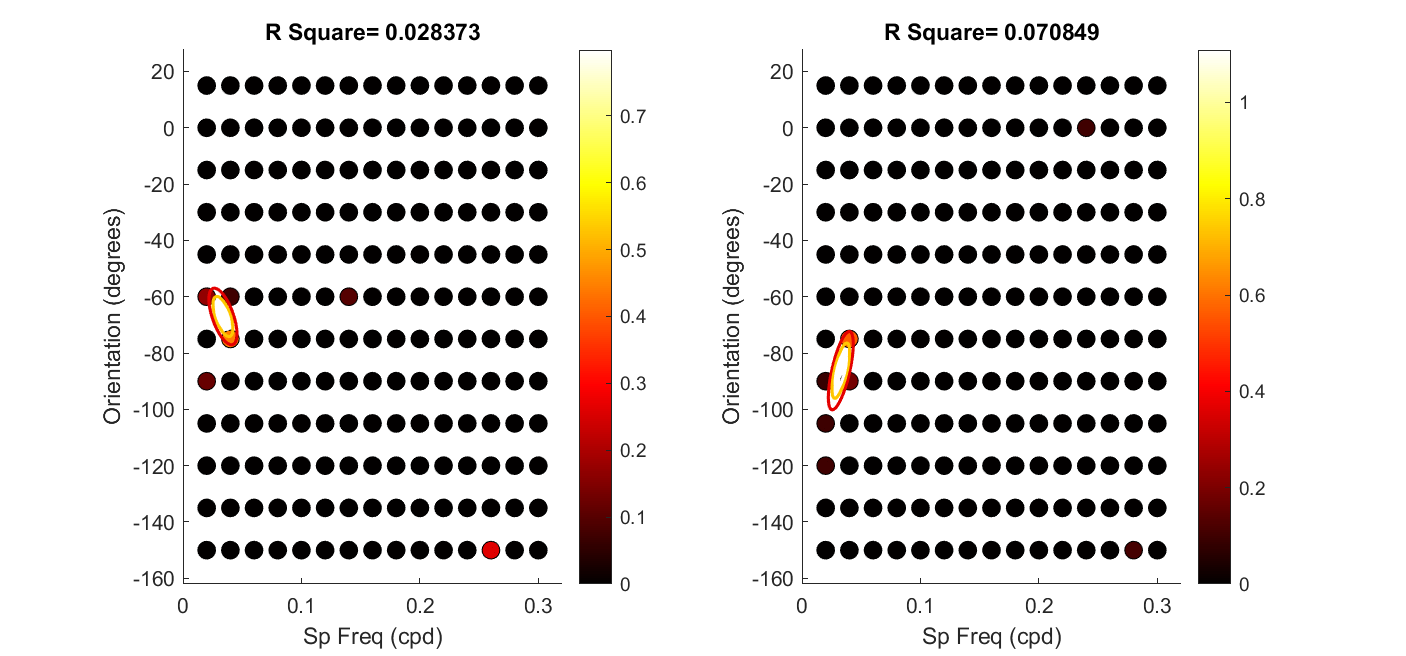

Supplement: Source data 1. — Tuning curves for all neurons scored as significantly tuned and tracked, for each of the three conditions: control, dark exposure (DE), and light reintroduction (LRx). [file elife-80361-data1.zip › SourceData1/b1_b2/2454_1R_cellPairID_37.png]

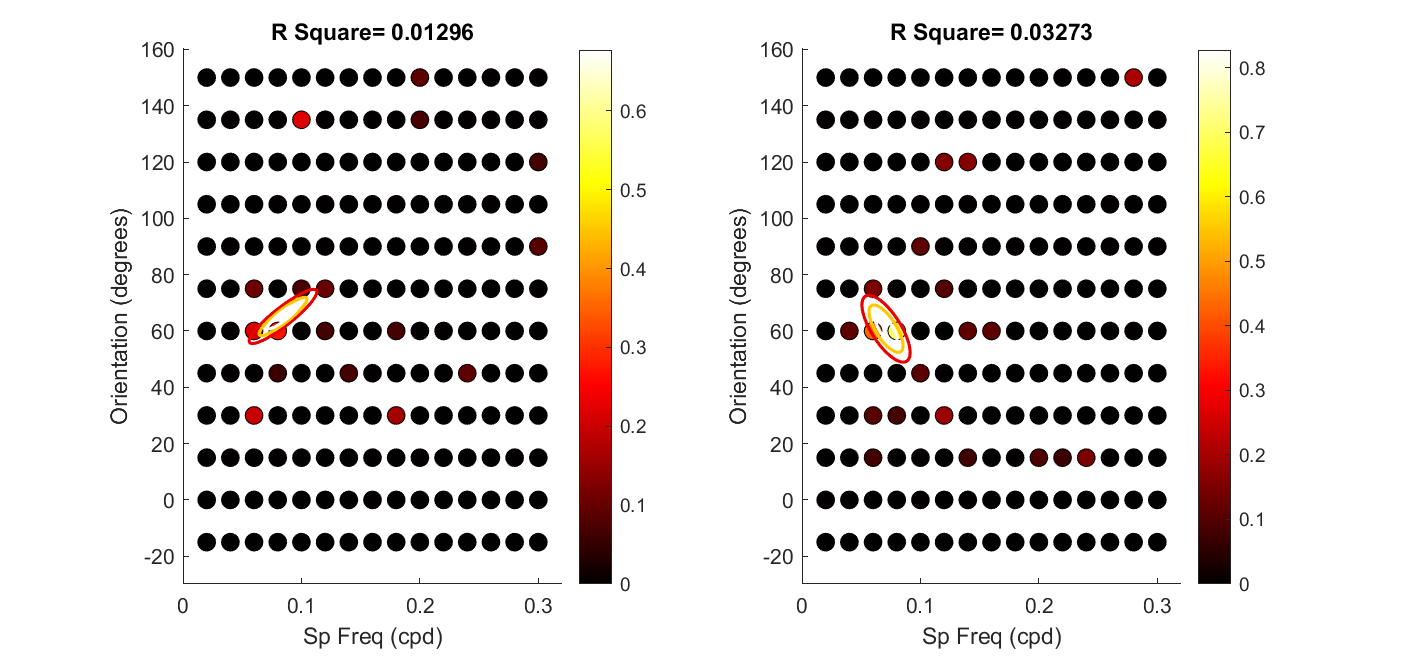

Supplement: Source data 1. — Tuning curves for all neurons scored as significantly tuned and tracked, for each of the three conditions: control, dark exposure (DE), and light reintroduction (LRx). [file elife-80361-data1.zip › SourceData1/b1_b2/2454_1R_cellPairID_38.png]

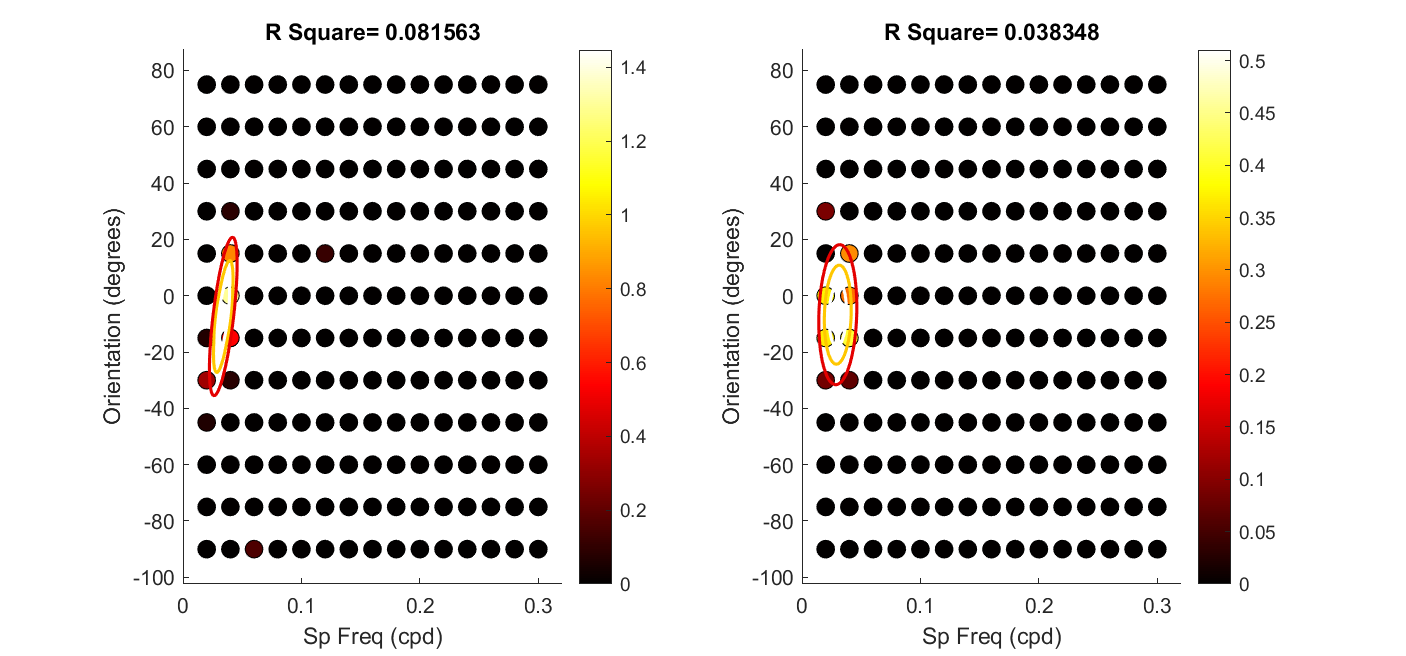

Supplement: Source data 1. — Tuning curves for all neurons scored as significantly tuned and tracked, for each of the three conditions: control, dark exposure (DE), and light reintroduction (LRx). [file elife-80361-data1.zip › SourceData1/b1_b2/2454_1R_cellPairID_39.png]

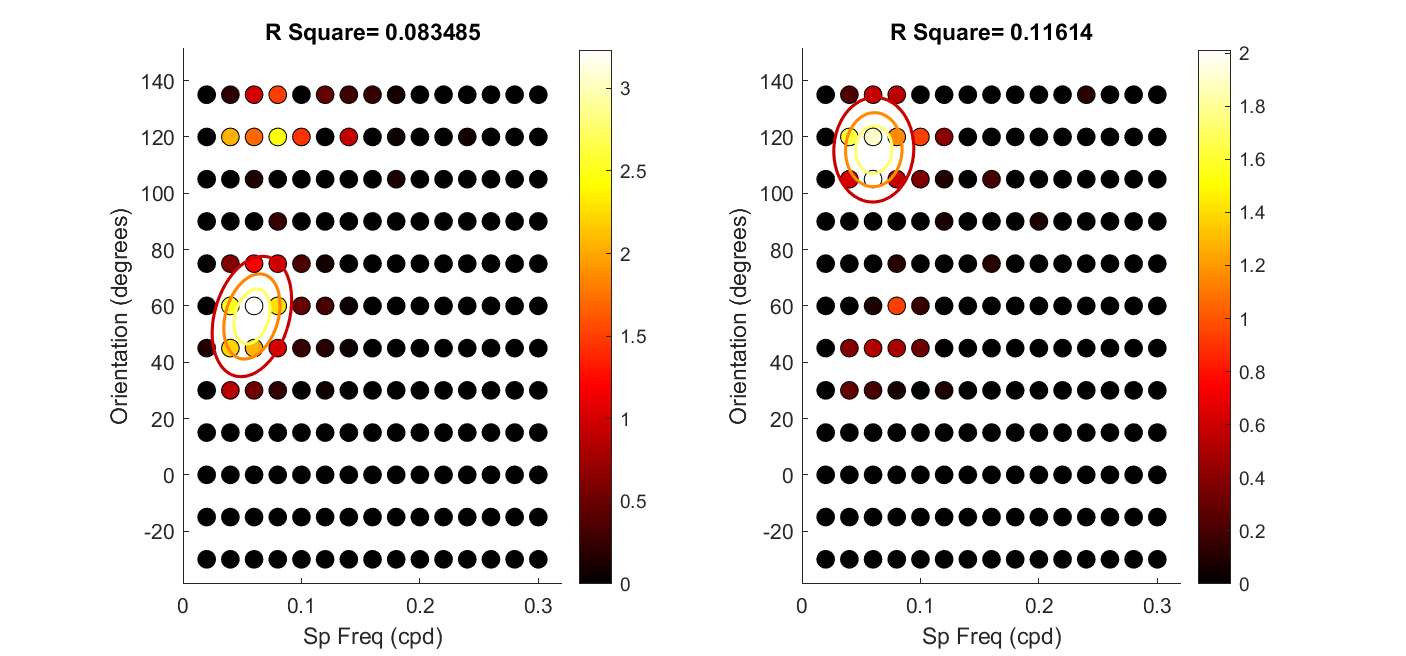

Supplement: Source data 1. — Tuning curves for all neurons scored as significantly tuned and tracked, for each of the three conditions: control, dark exposure (DE), and light reintroduction (LRx). [file elife-80361-data1.zip › SourceData1/b1_b2/2454_1R_cellPairID_4.png]

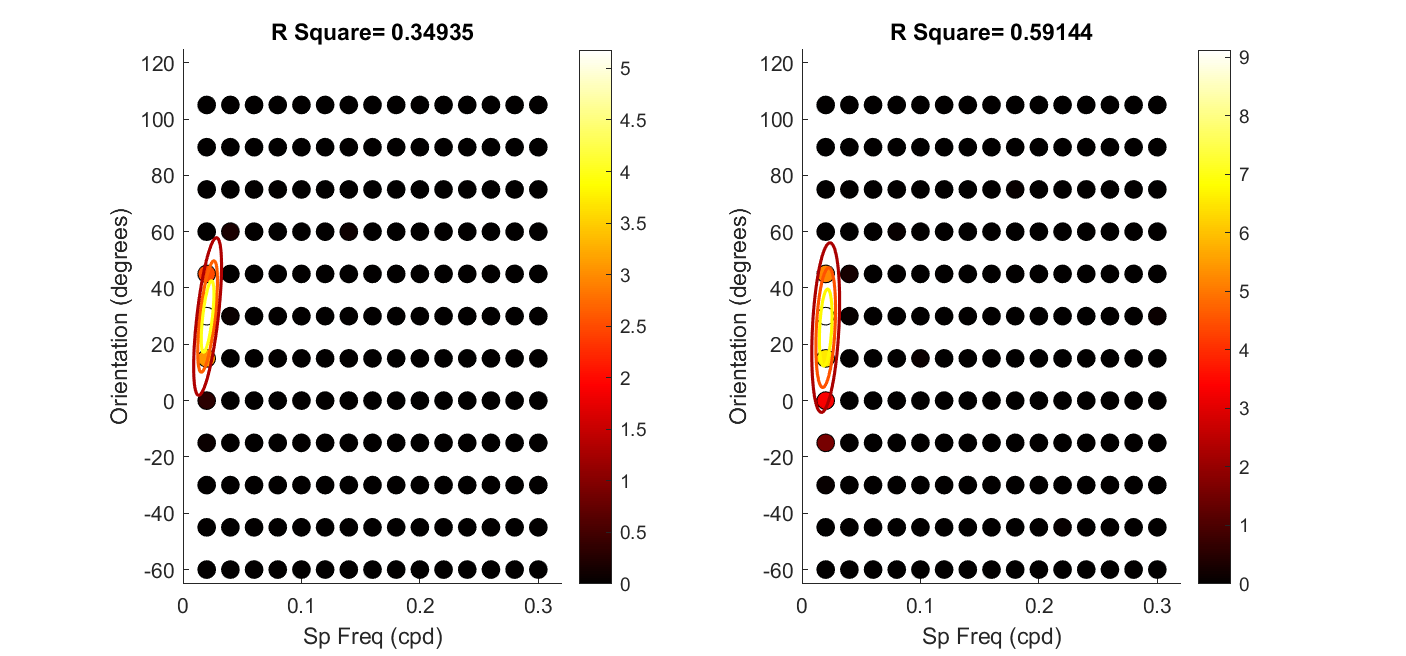

Supplement: Source data 1. — Tuning curves for all neurons scored as significantly tuned and tracked, for each of the three conditions: control, dark exposure (DE), and light reintroduction (LRx). [file elife-80361-data1.zip › SourceData1/b1_b2/2454_1R_cellPairID_5.png]

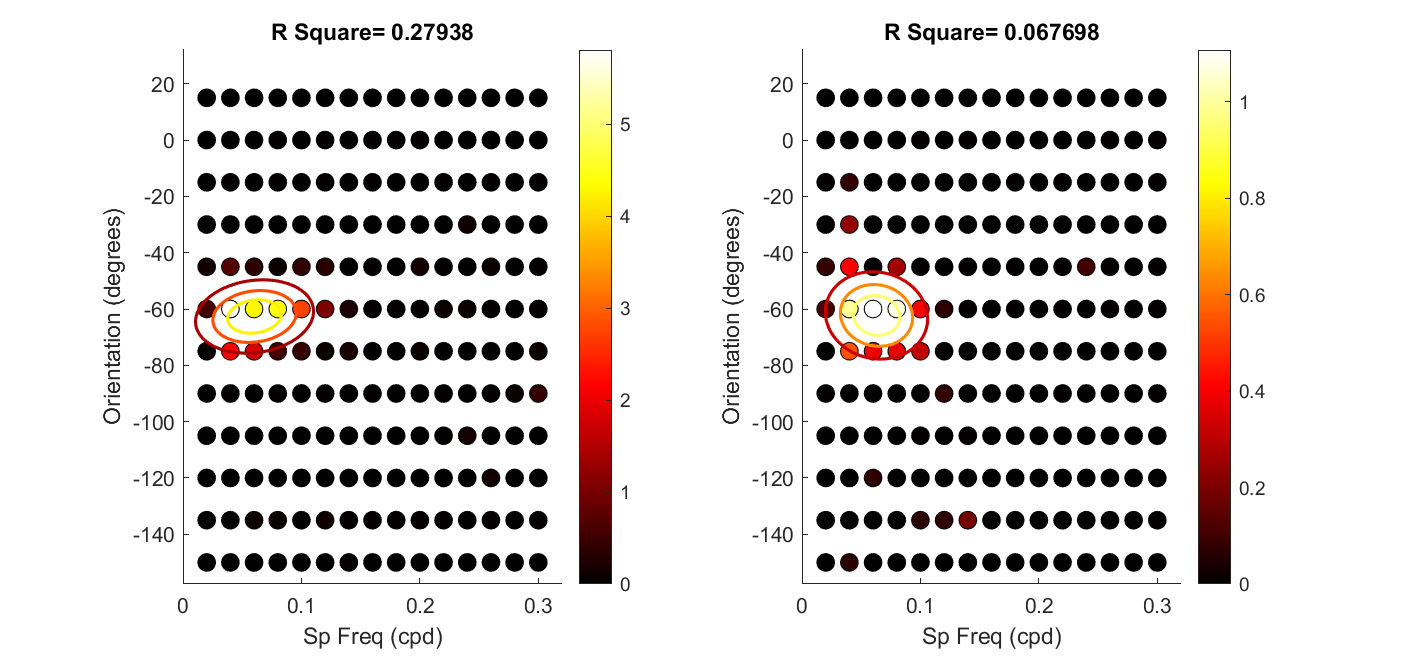

Supplement: Source data 1. — Tuning curves for all neurons scored as significantly tuned and tracked, for each of the three conditions: control, dark exposure (DE), and light reintroduction (LRx). [file elife-80361-data1.zip › SourceData1/b1_b2/2454_1R_cellPairID_6.png]

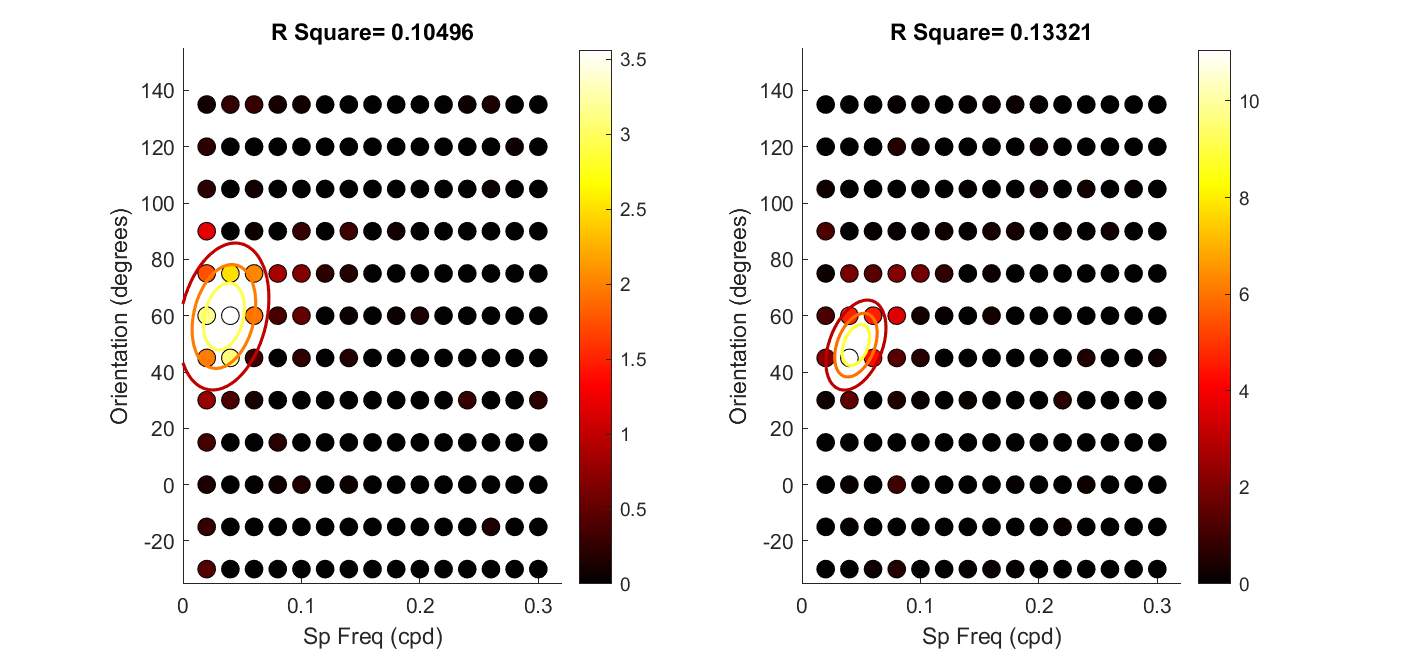

Supplement: Source data 1. — Tuning curves for all neurons scored as significantly tuned and tracked, for each of the three conditions: control, dark exposure (DE), and light reintroduction (LRx). [file elife-80361-data1.zip › SourceData1/b1_b2/2454_1R_cellPairID_7.png]

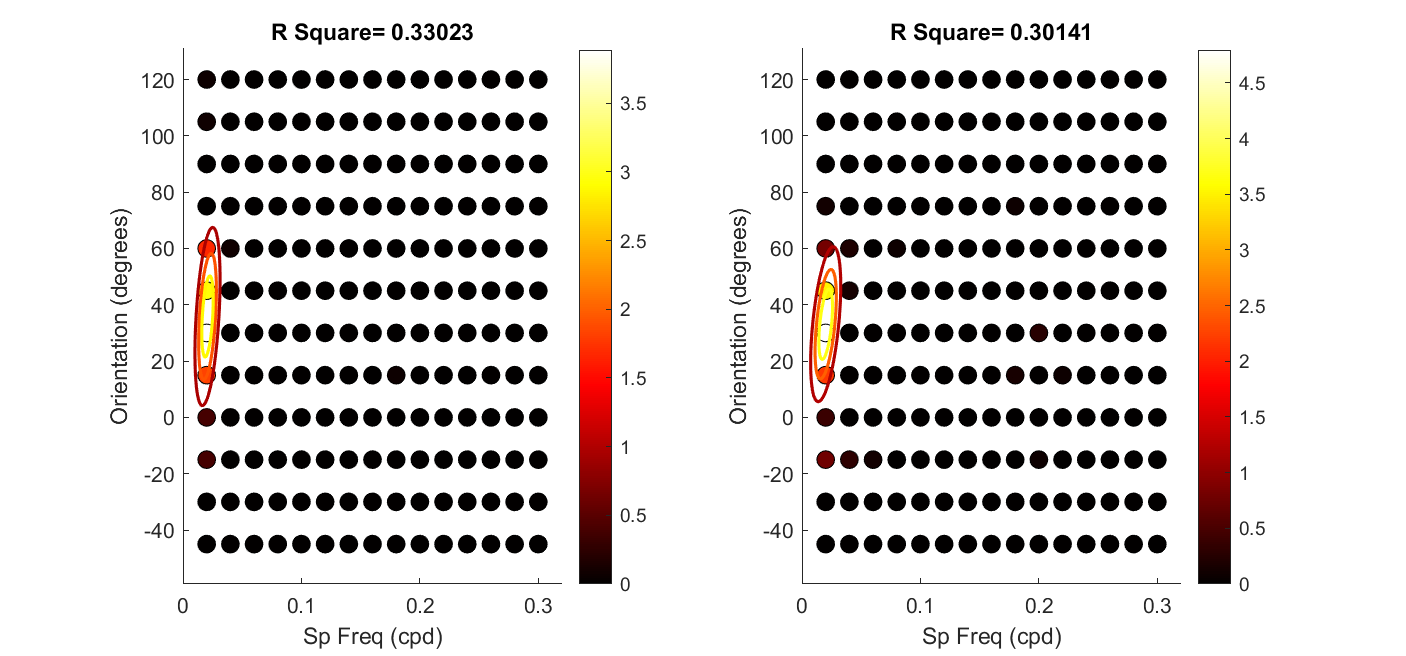

Supplement: Source data 1. — Tuning curves for all neurons scored as significantly tuned and tracked, for each of the three conditions: control, dark exposure (DE), and light reintroduction (LRx). [file elife-80361-data1.zip › SourceData1/b1_b2/2454_1R_cellPairID_8.png]

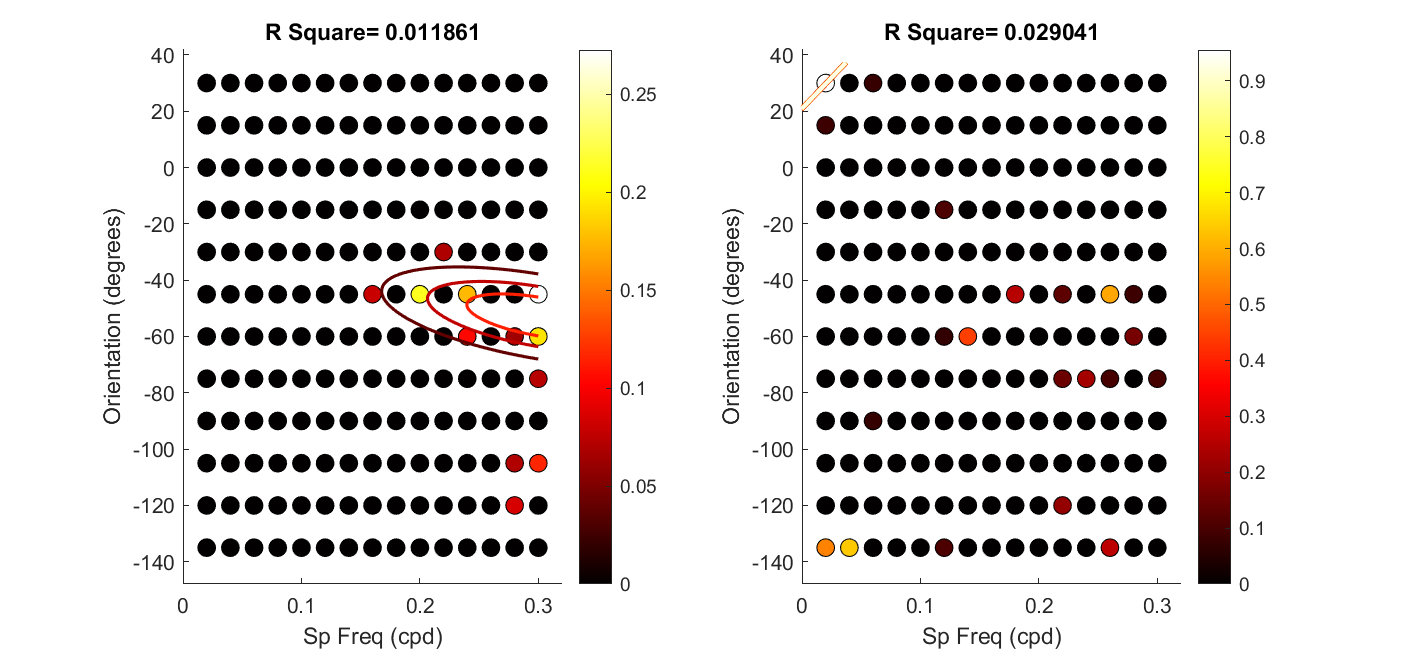

Supplement: Source data 1. — Tuning curves for all neurons scored as significantly tuned and tracked, for each of the three conditions: control, dark exposure (DE), and light reintroduction (LRx). [file elife-80361-data1.zip › SourceData1/b1_b2/2454_1R_cellPairID_9.png]

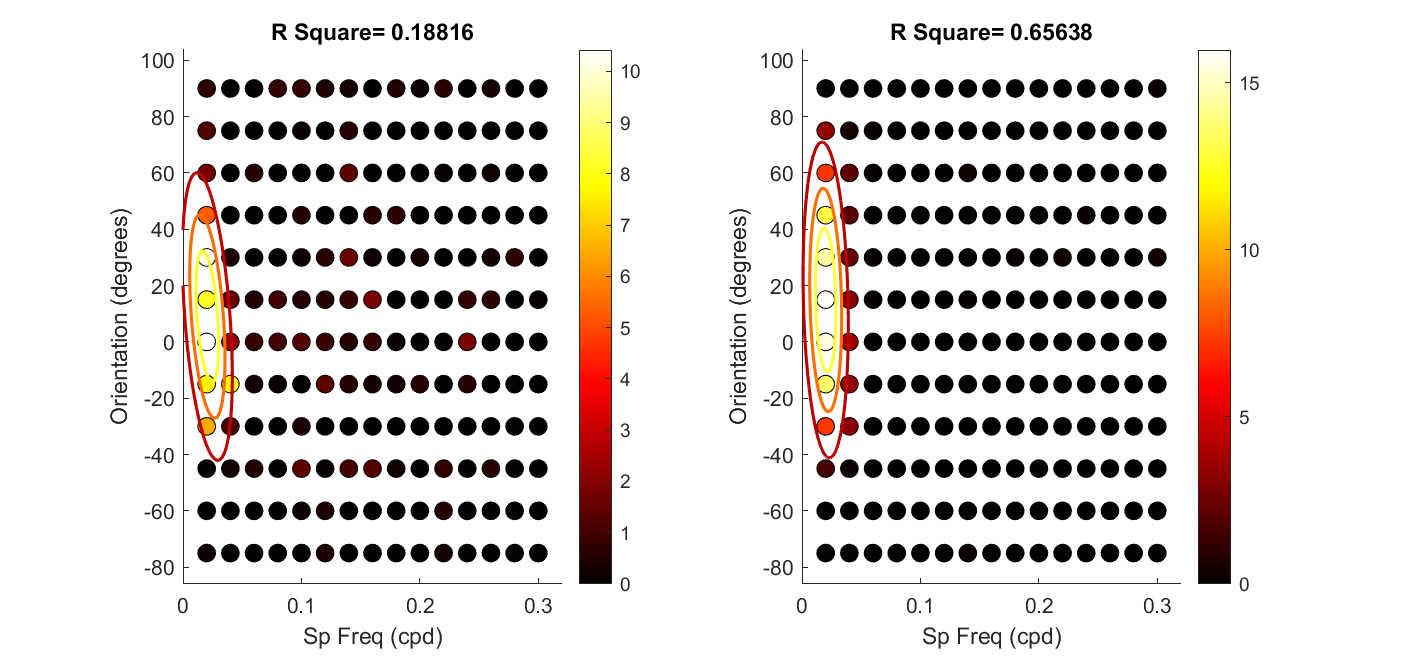

Supplement: Source data 1. — Tuning curves for all neurons scored as significantly tuned and tracked, for each of the three conditions: control, dark exposure (DE), and light reintroduction (LRx). [file elife-80361-data1.zip › SourceData1/b1_b2/2472_1L_cellPairID_1.png]

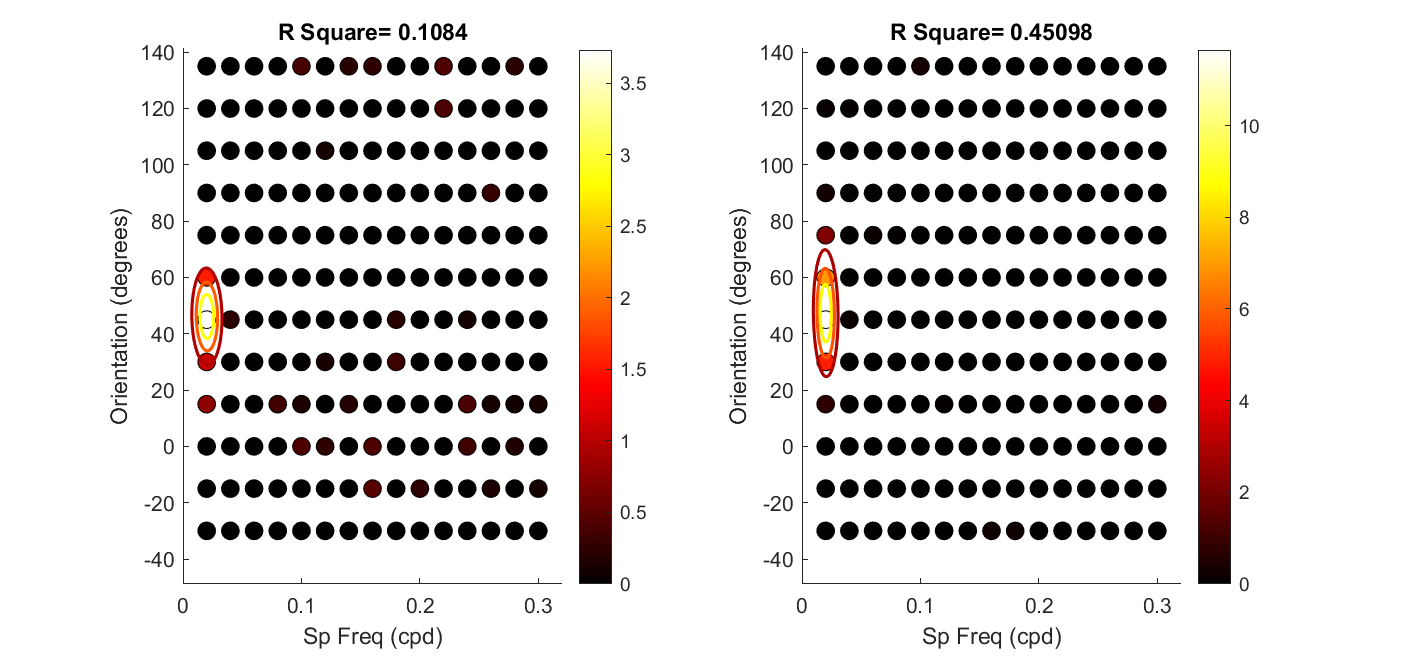

Supplement: Source data 1. — Tuning curves for all neurons scored as significantly tuned and tracked, for each of the three conditions: control, dark exposure (DE), and light reintroduction (LRx). [file elife-80361-data1.zip › SourceData1/b1_b2/2472_1L_cellPairID_10.png]

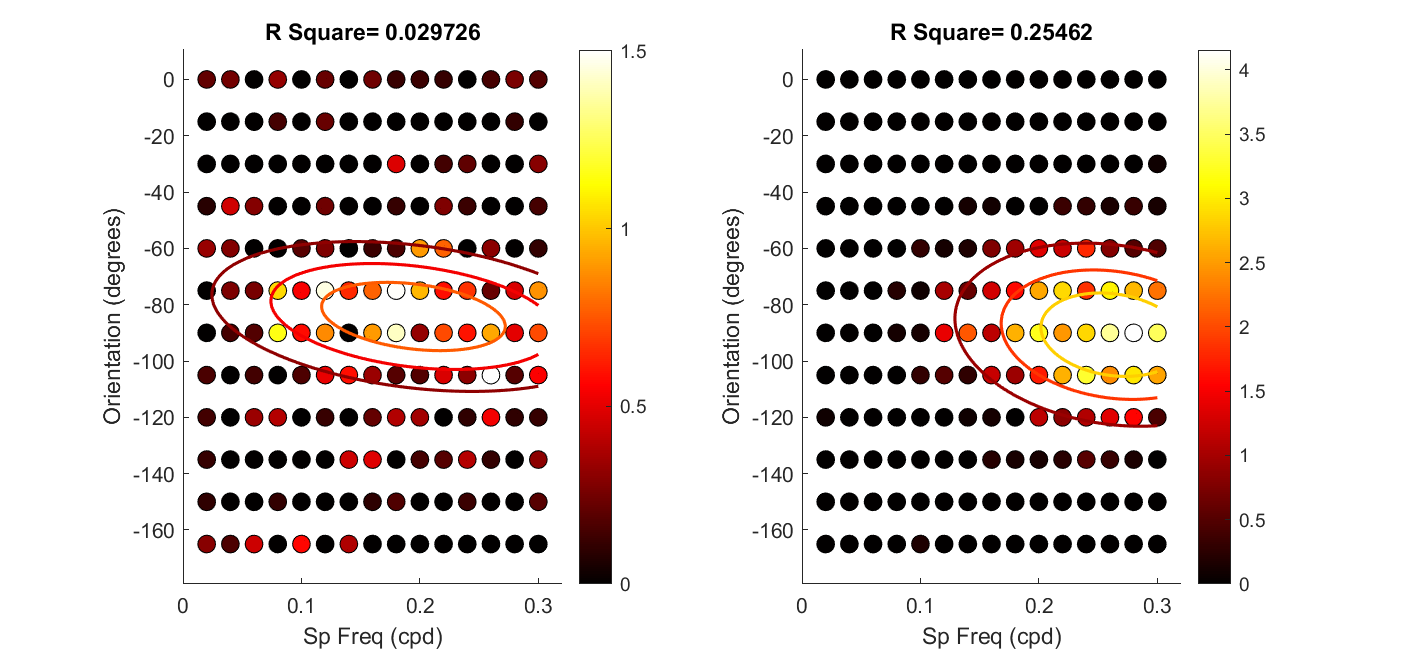

Supplement: Source data 1. — Tuning curves for all neurons scored as significantly tuned and tracked, for each of the three conditions: control, dark exposure (DE), and light reintroduction (LRx). [file elife-80361-data1.zip › SourceData1/b1_b2/2472_1L_cellPairID_11.png]

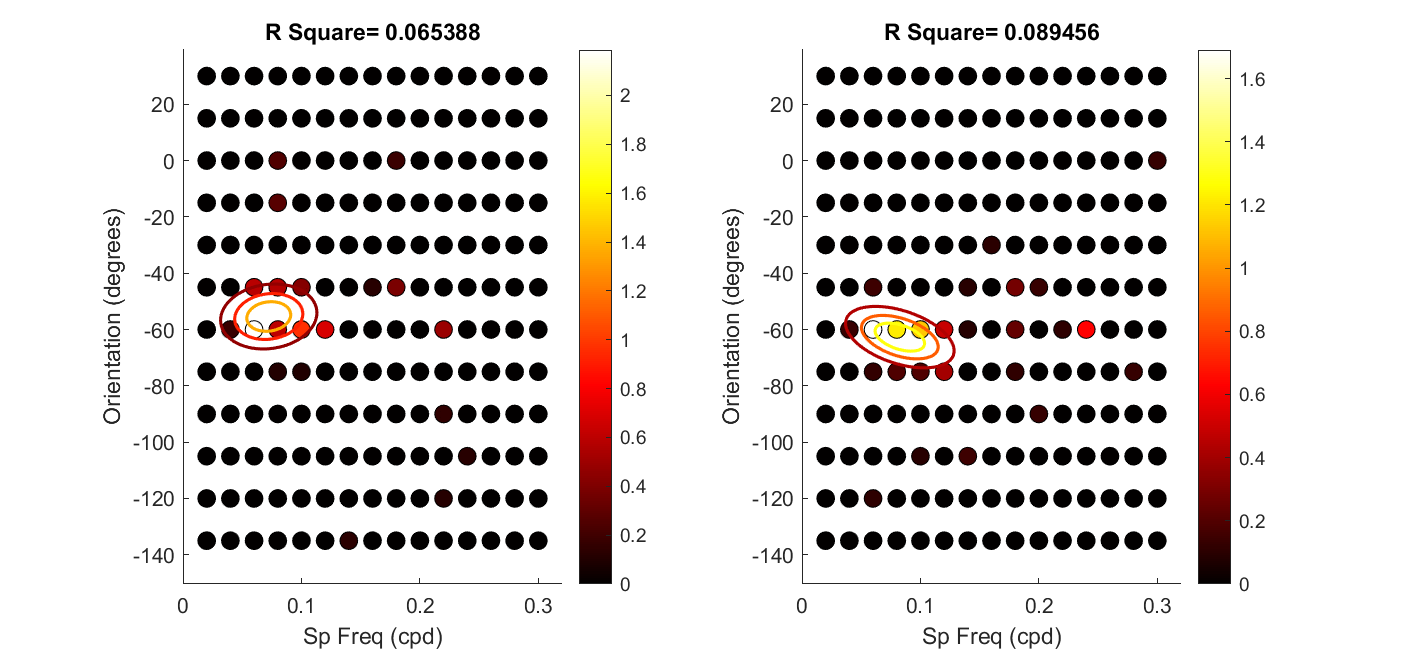

Supplement: Source data 1. — Tuning curves for all neurons scored as significantly tuned and tracked, for each of the three conditions: control, dark exposure (DE), and light reintroduction (LRx). [file elife-80361-data1.zip › SourceData1/b1_b2/2472_1L_cellPairID_12.png]

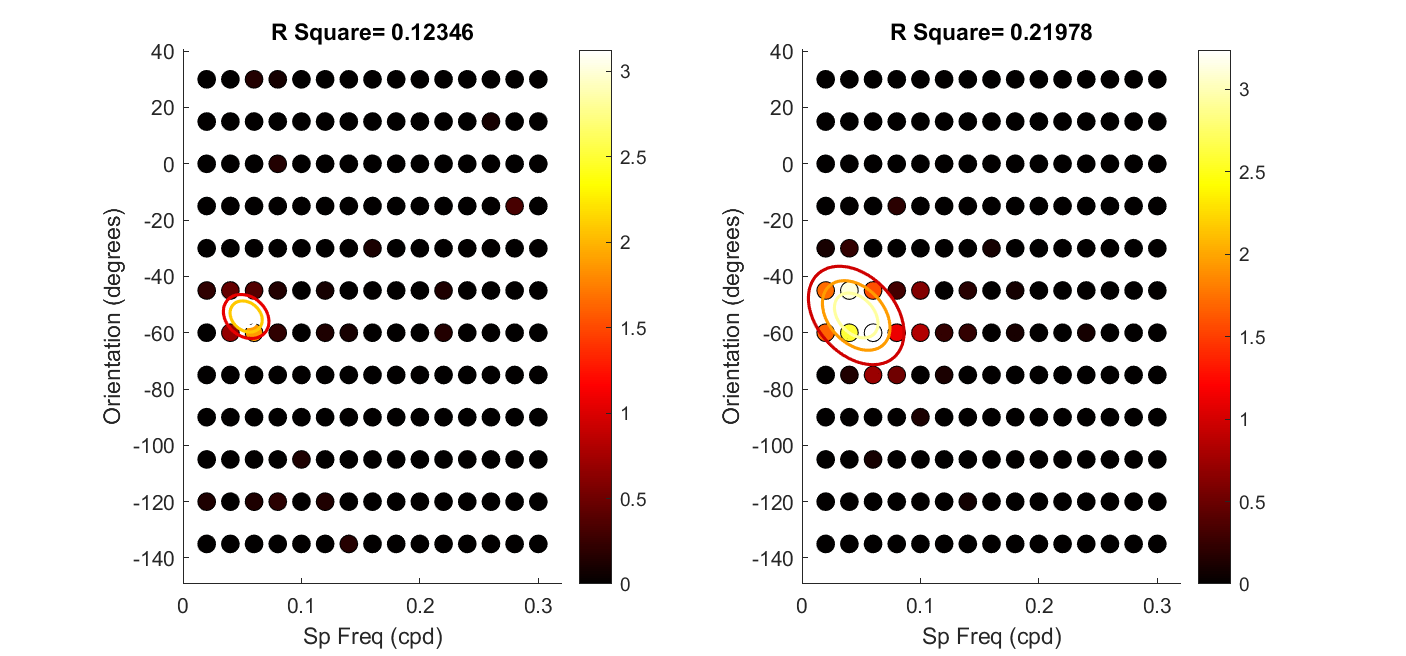

Supplement: Source data 1. — Tuning curves for all neurons scored as significantly tuned and tracked, for each of the three conditions: control, dark exposure (DE), and light reintroduction (LRx). [file elife-80361-data1.zip › SourceData1/b1_b2/2472_1L_cellPairID_13.png]

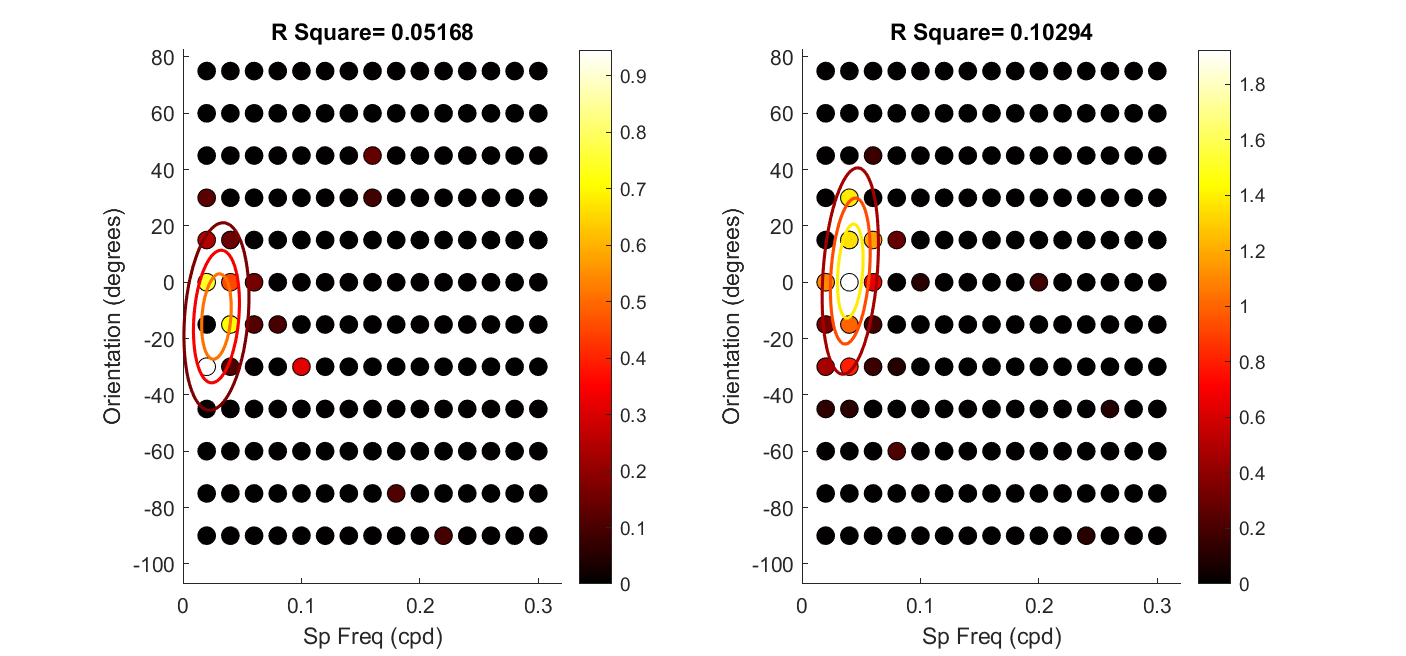

Supplement: Source data 1. — Tuning curves for all neurons scored as significantly tuned and tracked, for each of the three conditions: control, dark exposure (DE), and light reintroduction (LRx). [file elife-80361-data1.zip › SourceData1/b1_b2/2472_1L_cellPairID_14.png]

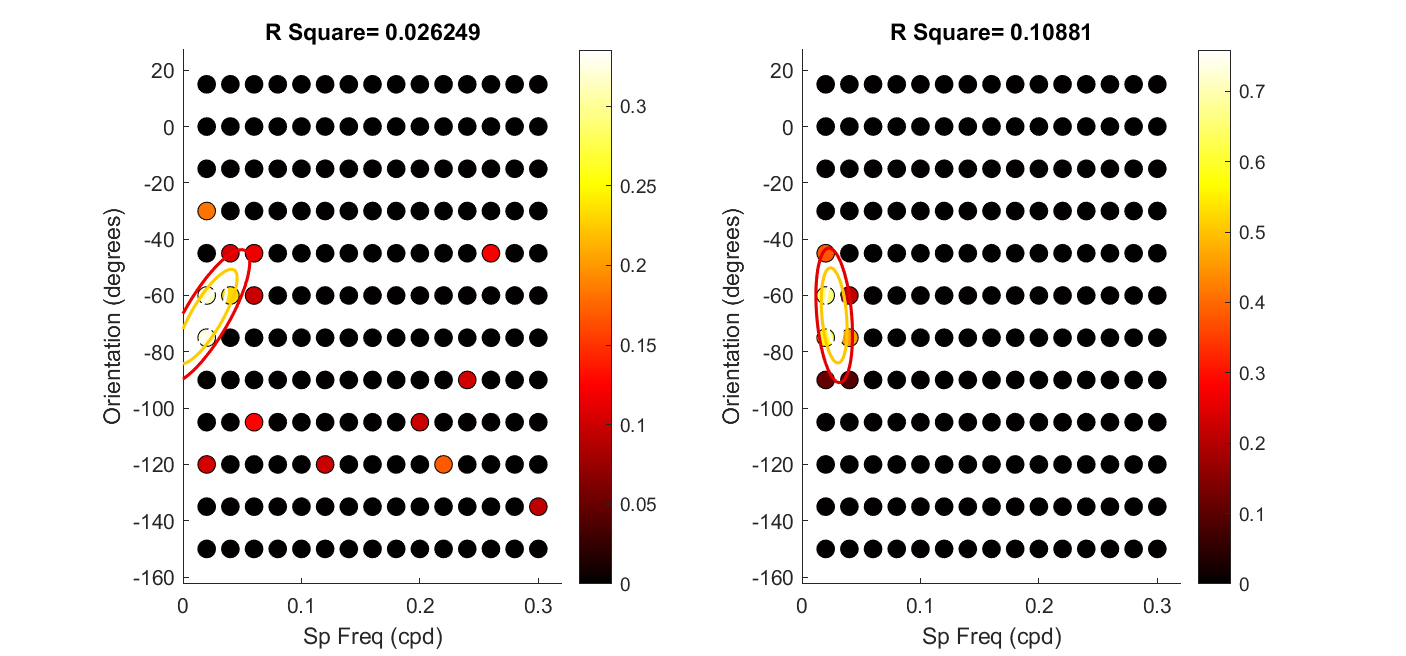

Supplement: Source data 1. — Tuning curves for all neurons scored as significantly tuned and tracked, for each of the three conditions: control, dark exposure (DE), and light reintroduction (LRx). [file elife-80361-data1.zip › SourceData1/b1_b2/2472_1L_cellPairID_15.png]

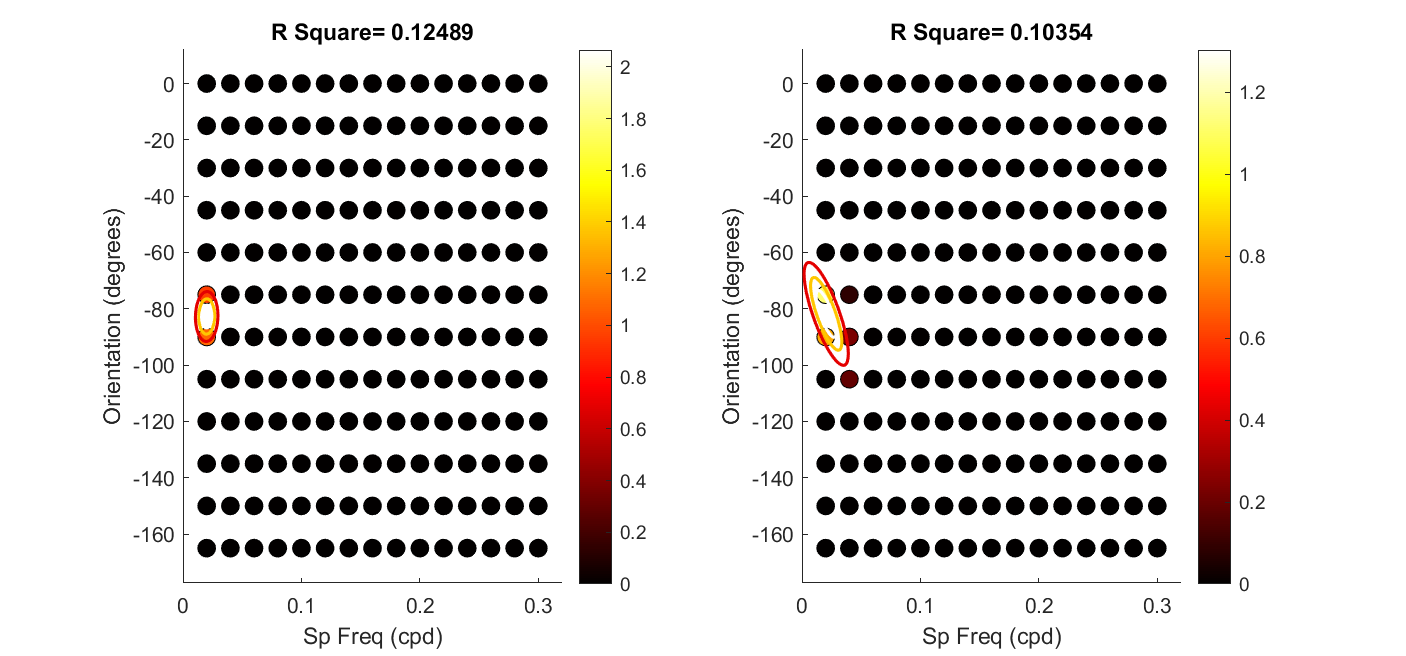

Supplement: Source data 1. — Tuning curves for all neurons scored as significantly tuned and tracked, for each of the three conditions: control, dark exposure (DE), and light reintroduction (LRx). [file elife-80361-data1.zip › SourceData1/b1_b2/2472_1L_cellPairID_16.png]

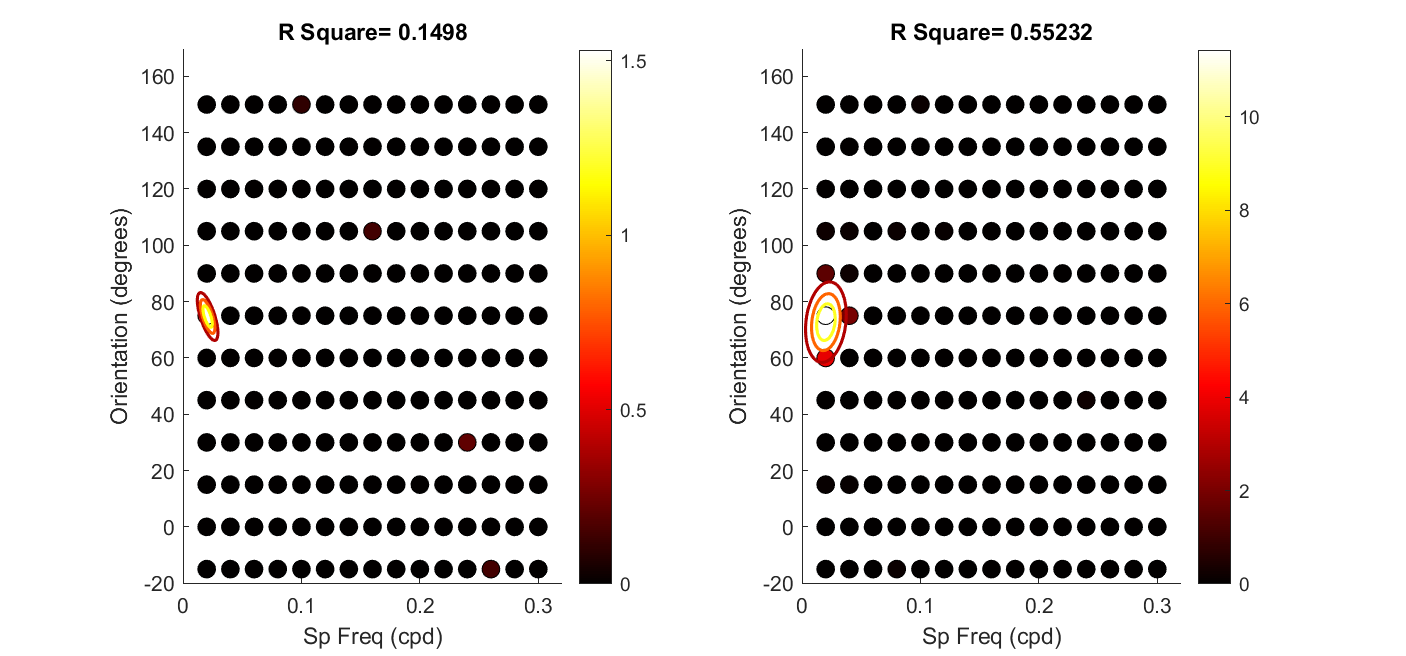

Supplement: Source data 1. — Tuning curves for all neurons scored as significantly tuned and tracked, for each of the three conditions: control, dark exposure (DE), and light reintroduction (LRx). [file elife-80361-data1.zip › SourceData1/b1_b2/2472_1L_cellPairID_17.png]

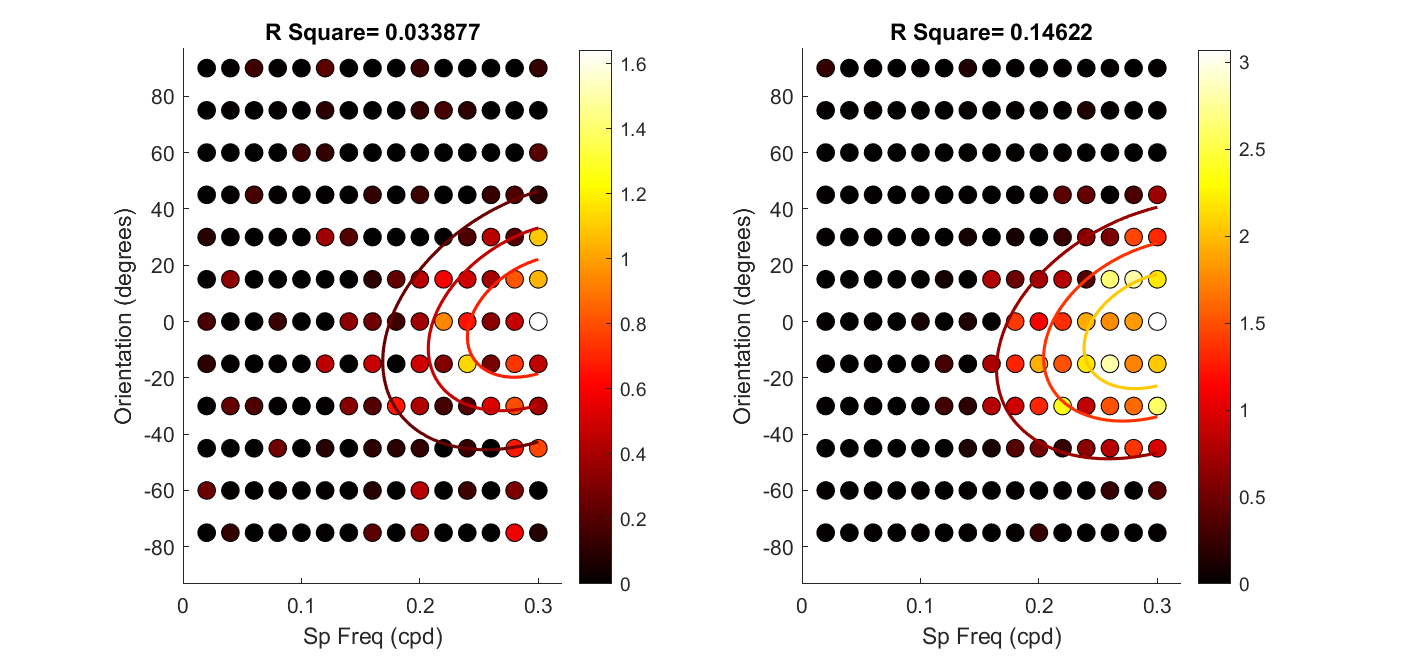

Supplement: Source data 1. — Tuning curves for all neurons scored as significantly tuned and tracked, for each of the three conditions: control, dark exposure (DE), and light reintroduction (LRx). [file elife-80361-data1.zip › SourceData1/b1_b2/2472_1L_cellPairID_18.png]

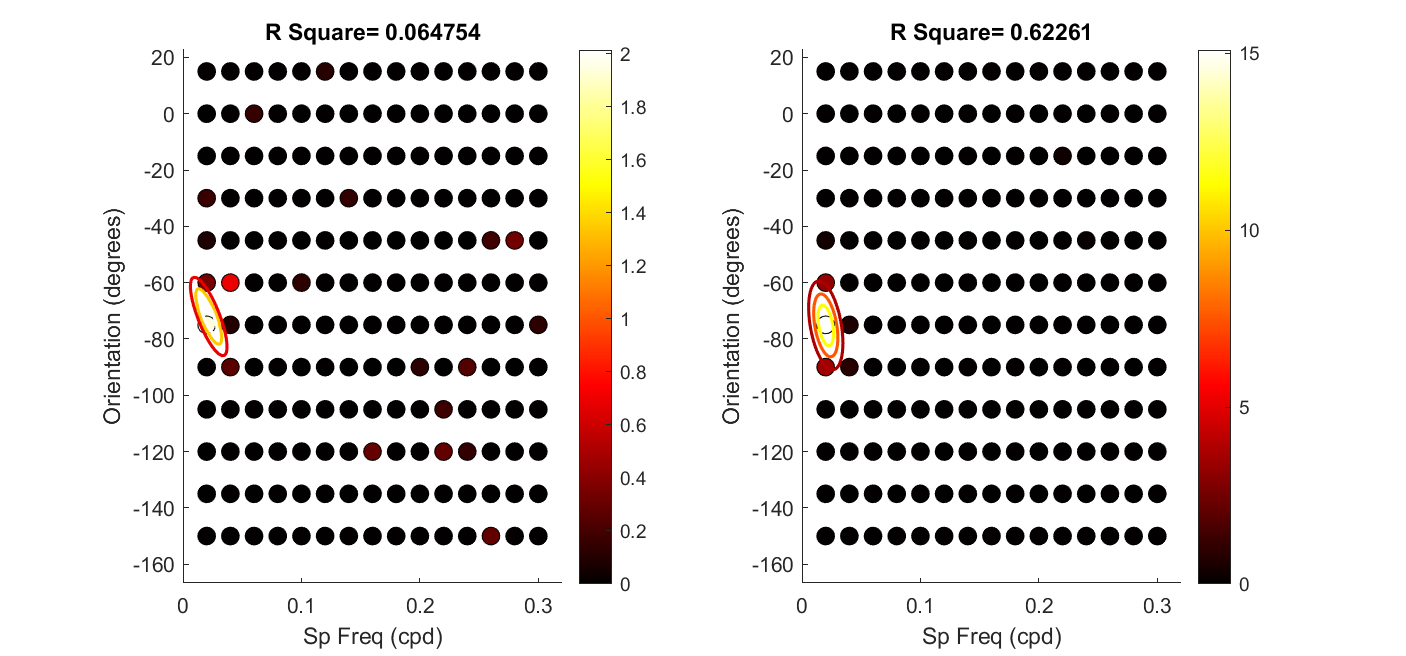

Supplement: Source data 1. — Tuning curves for all neurons scored as significantly tuned and tracked, for each of the three conditions: control, dark exposure (DE), and light reintroduction (LRx). [file elife-80361-data1.zip › SourceData1/b1_b2/2472_1L_cellPairID_19.png]

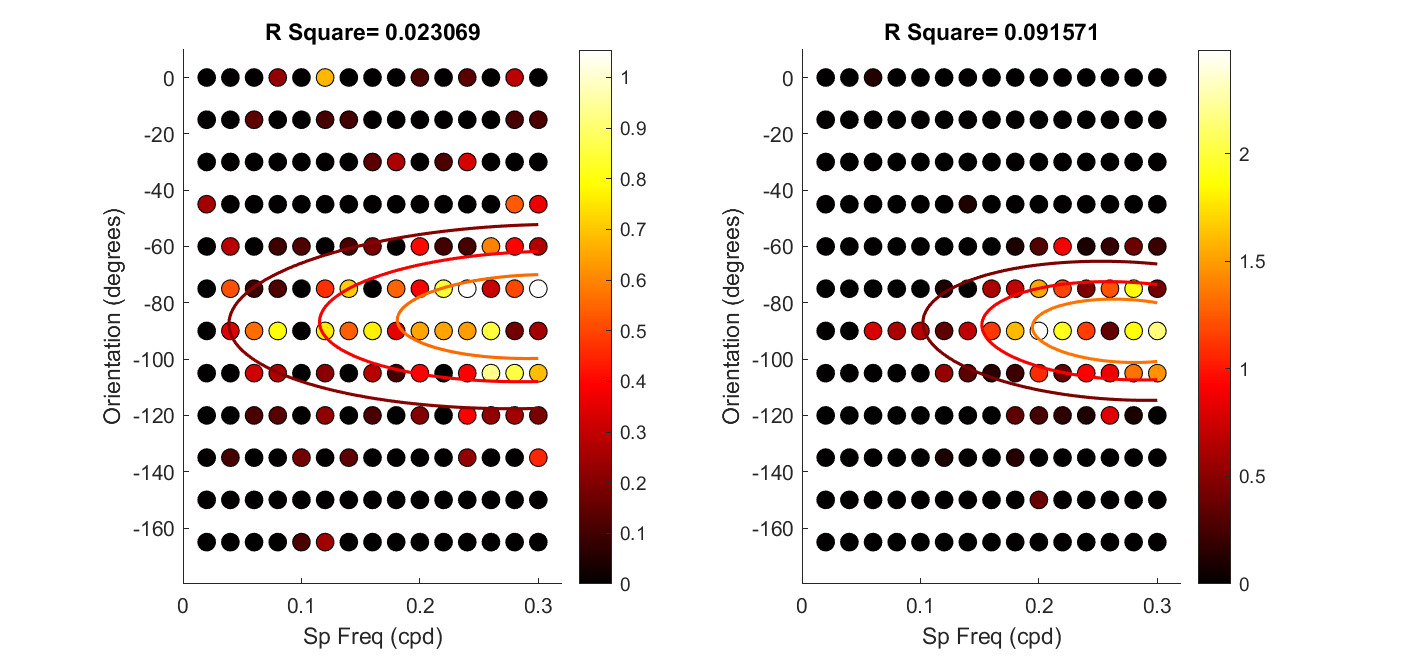

Supplement: Source data 1. — Tuning curves for all neurons scored as significantly tuned and tracked, for each of the three conditions: control, dark exposure (DE), and light reintroduction (LRx). [file elife-80361-data1.zip › SourceData1/b1_b2/2472_1L_cellPairID_2.png]

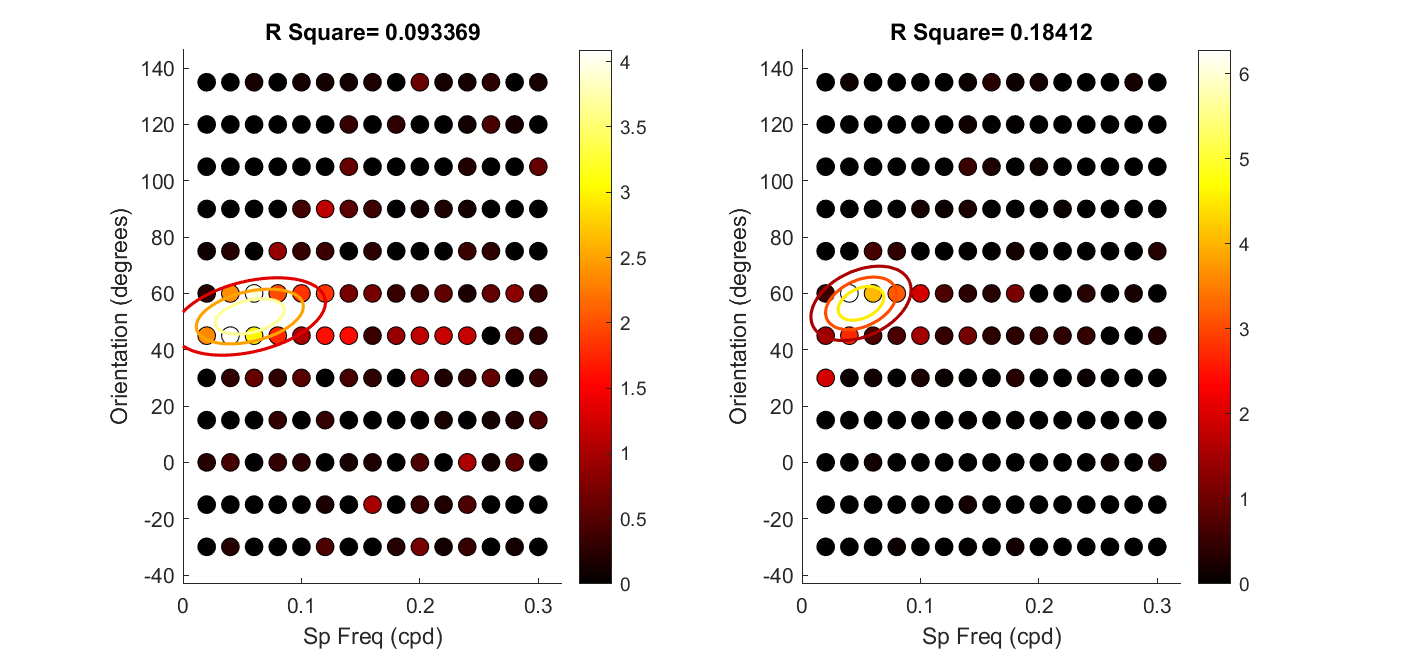

Supplement: Source data 1. — Tuning curves for all neurons scored as significantly tuned and tracked, for each of the three conditions: control, dark exposure (DE), and light reintroduction (LRx). [file elife-80361-data1.zip › SourceData1/b1_b2/2472_1L_cellPairID_20.png]

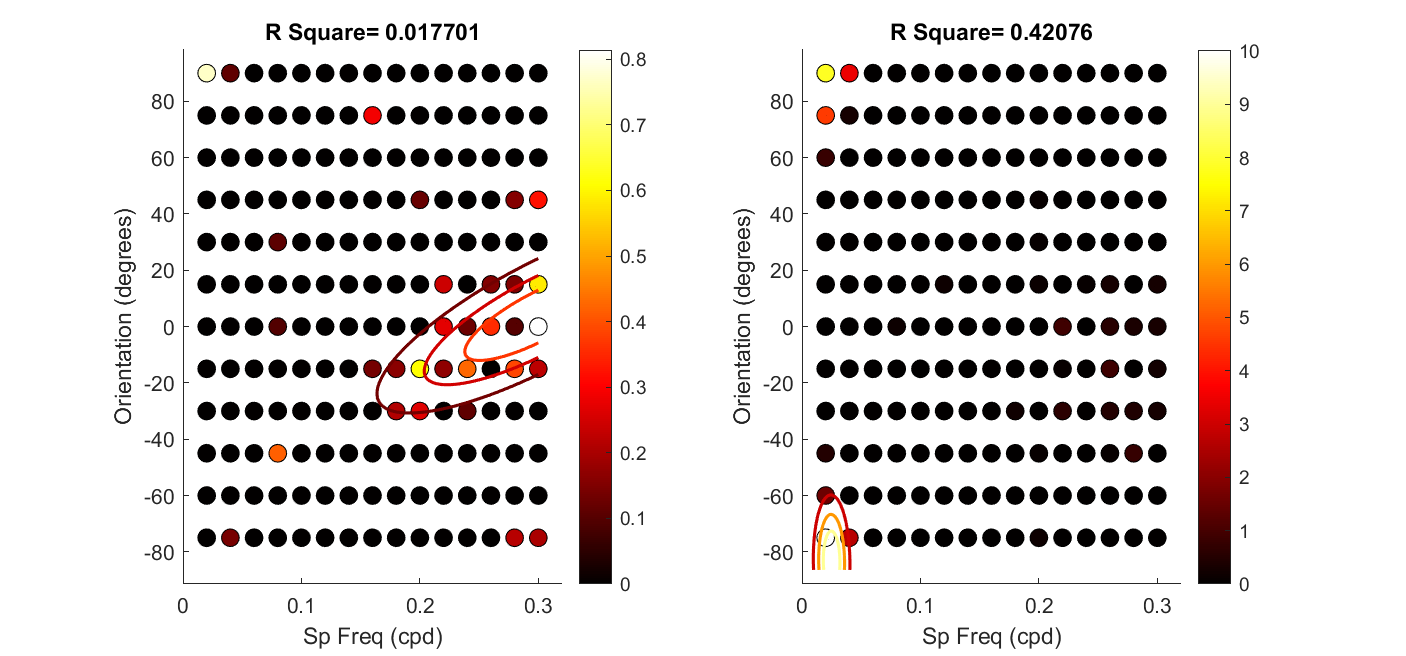

Supplement: Source data 1. — Tuning curves for all neurons scored as significantly tuned and tracked, for each of the three conditions: control, dark exposure (DE), and light reintroduction (LRx). [file elife-80361-data1.zip › SourceData1/b1_b2/2472_1L_cellPairID_21.png]

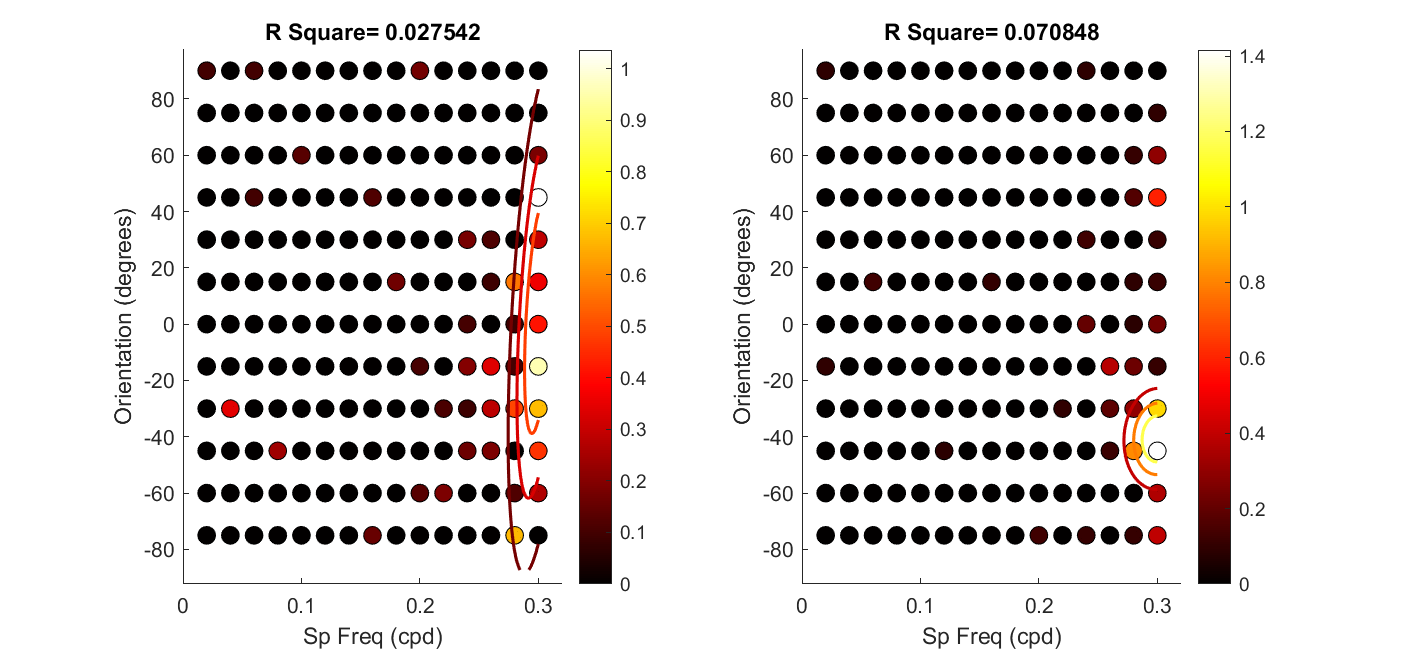

Supplement: Source data 1. — Tuning curves for all neurons scored as significantly tuned and tracked, for each of the three conditions: control, dark exposure (DE), and light reintroduction (LRx). [file elife-80361-data1.zip › SourceData1/b1_b2/2472_1L_cellPairID_22.png]

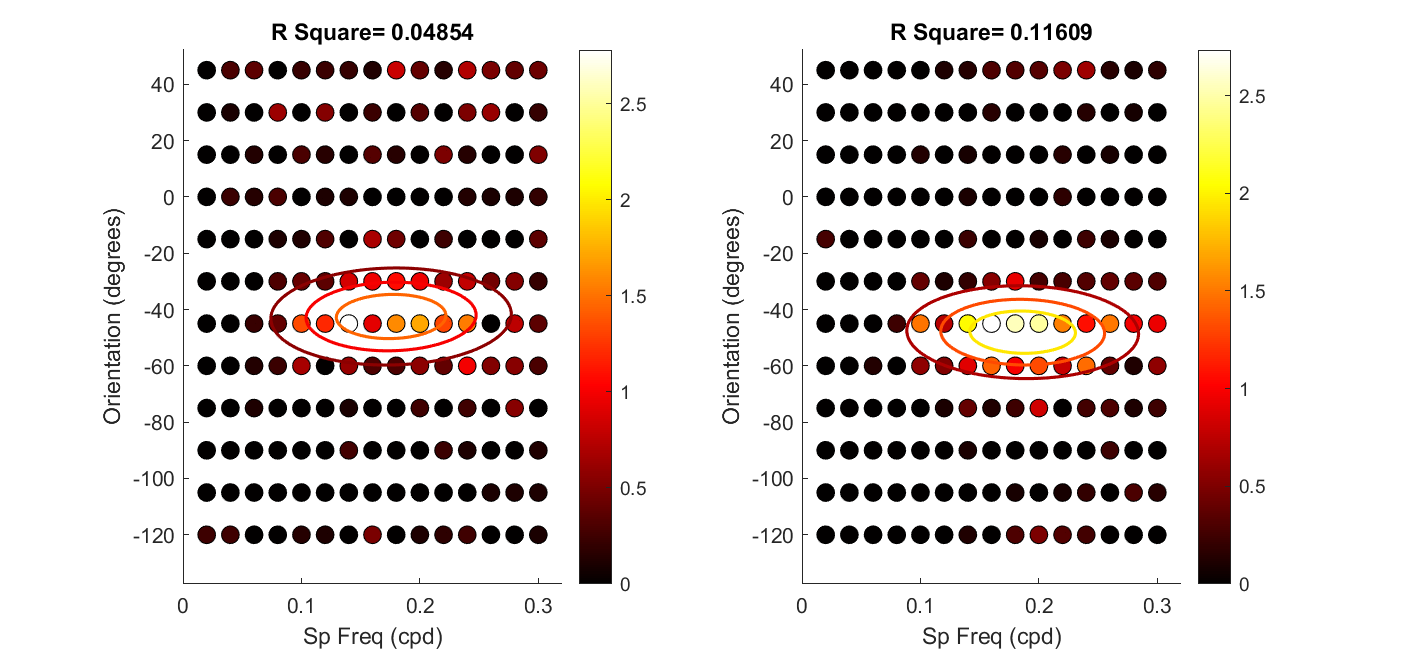

Supplement: Source data 1. — Tuning curves for all neurons scored as significantly tuned and tracked, for each of the three conditions: control, dark exposure (DE), and light reintroduction (LRx). [file elife-80361-data1.zip › SourceData1/b1_b2/2472_1L_cellPairID_23.png]

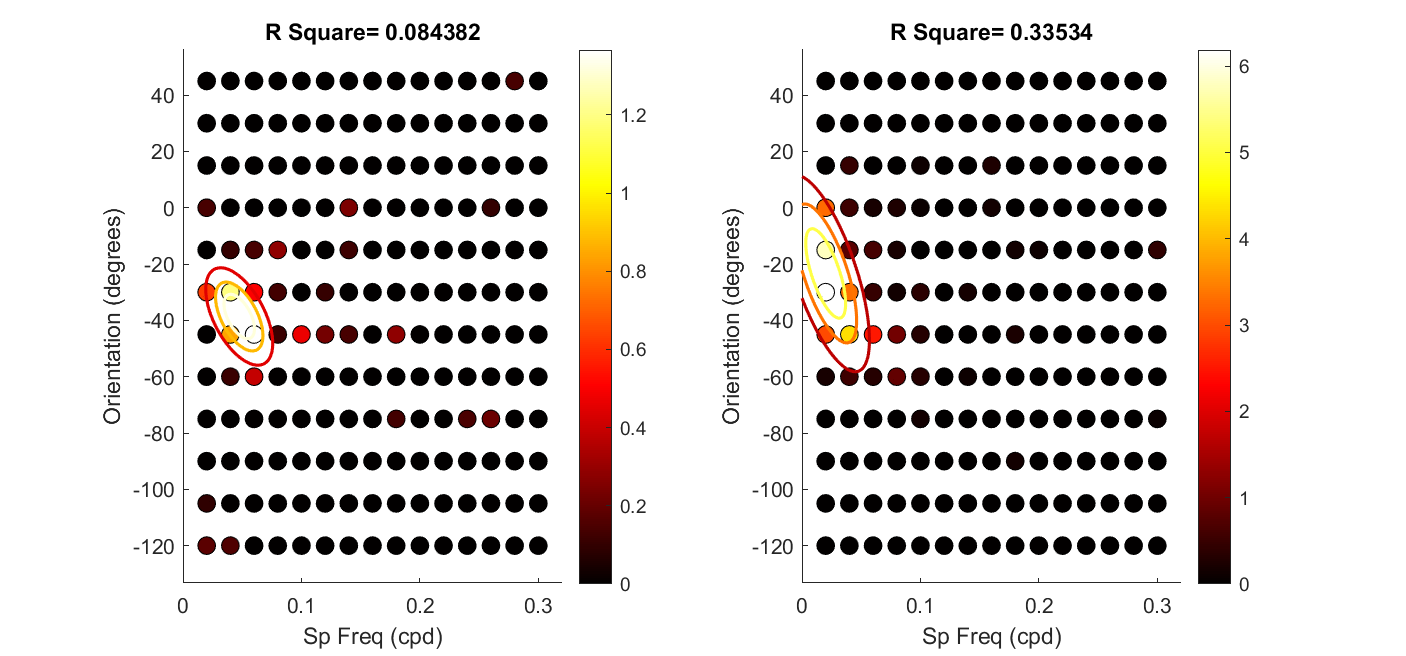

Supplement: Source data 1. — Tuning curves for all neurons scored as significantly tuned and tracked, for each of the three conditions: control, dark exposure (DE), and light reintroduction (LRx). [file elife-80361-data1.zip › SourceData1/b1_b2/2472_1L_cellPairID_24.png]

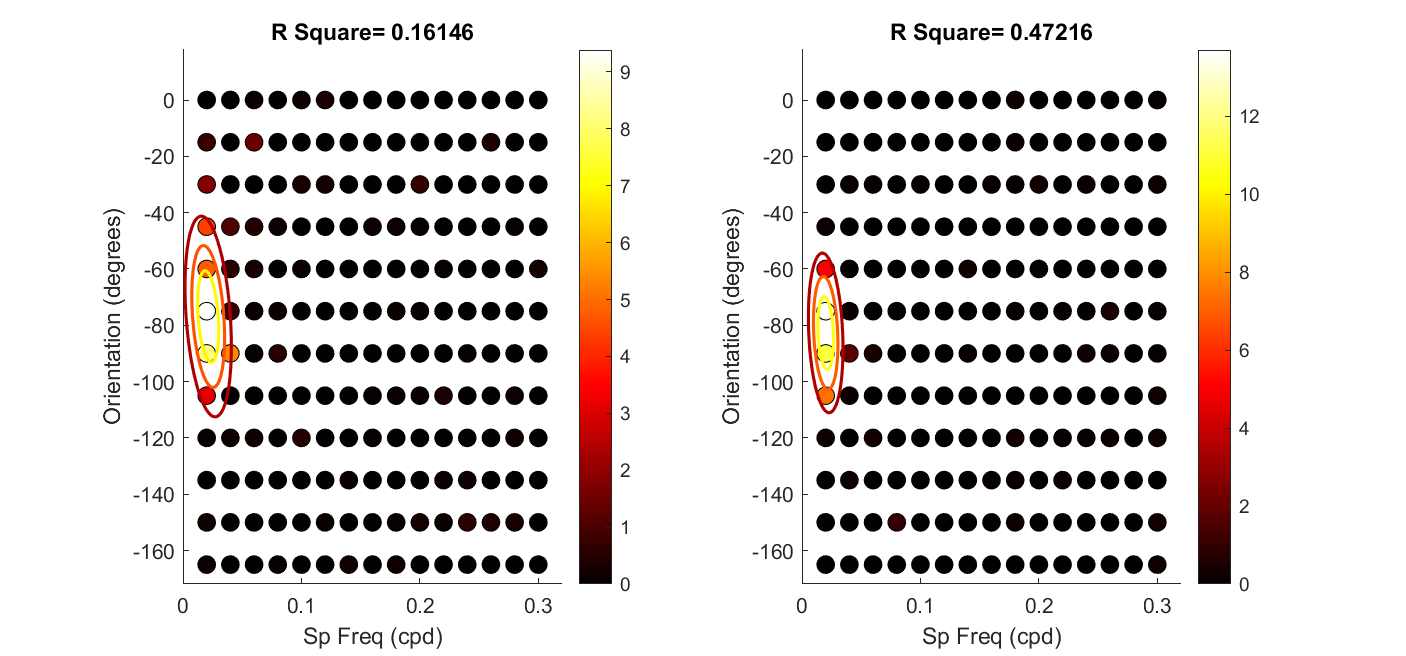

Supplement: Source data 1. — Tuning curves for all neurons scored as significantly tuned and tracked, for each of the three conditions: control, dark exposure (DE), and light reintroduction (LRx). [file elife-80361-data1.zip › SourceData1/b1_b2/2472_1L_cellPairID_25.png]

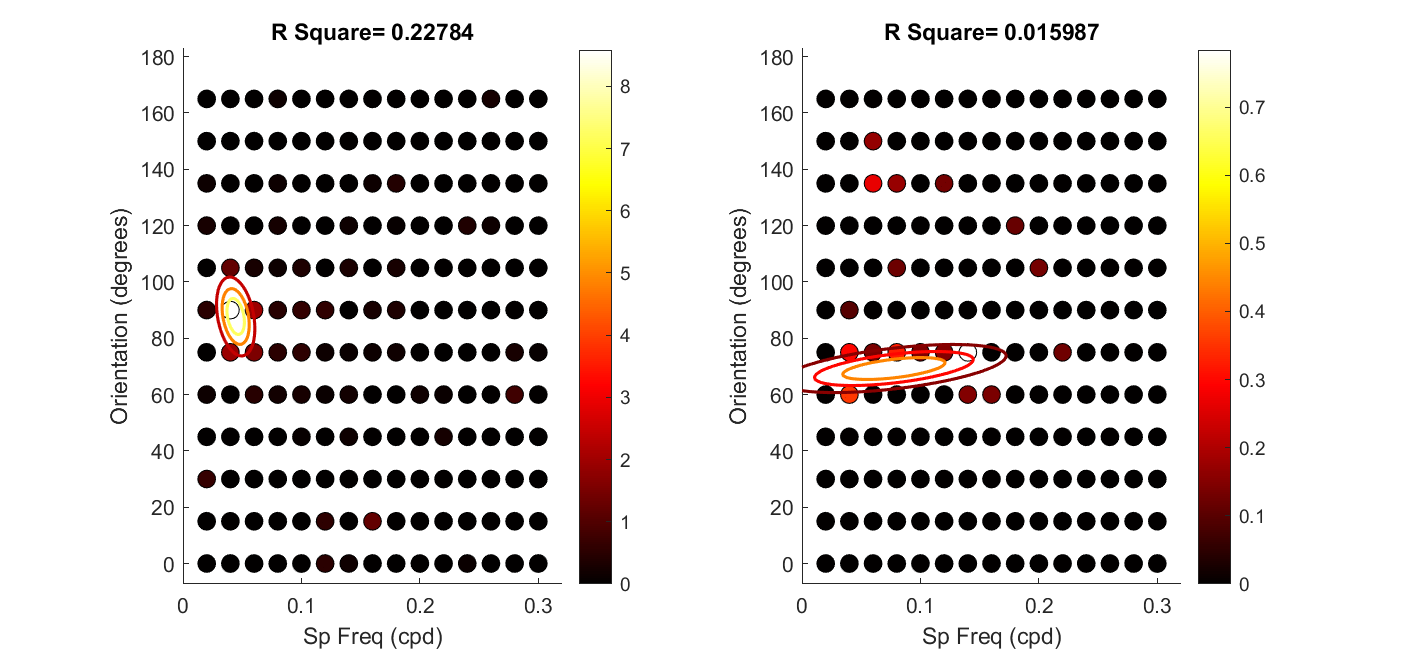

Supplement: Source data 1. — Tuning curves for all neurons scored as significantly tuned and tracked, for each of the three conditions: control, dark exposure (DE), and light reintroduction (LRx). [file elife-80361-data1.zip › SourceData1/b1_b2/2472_1L_cellPairID_26.png]

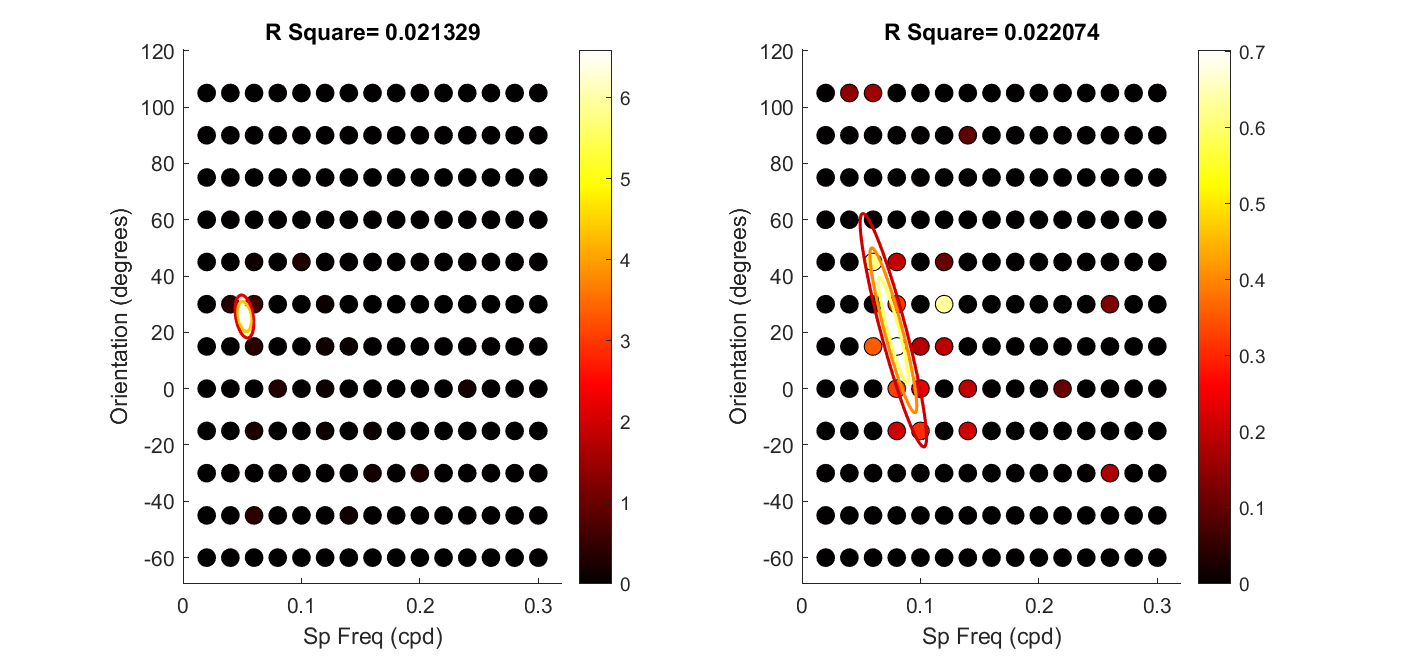

Supplement: Source data 1. — Tuning curves for all neurons scored as significantly tuned and tracked, for each of the three conditions: control, dark exposure (DE), and light reintroduction (LRx). [file elife-80361-data1.zip › SourceData1/b1_b2/2472_1L_cellPairID_27.png]

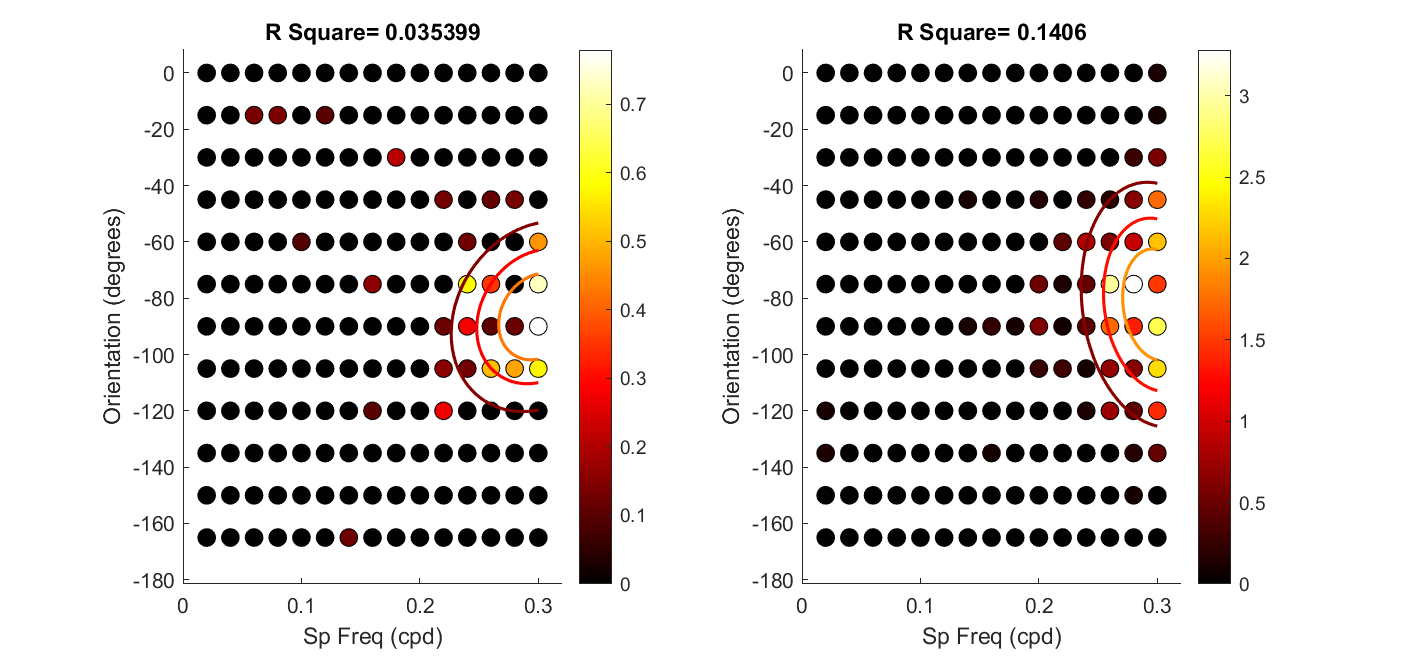

Supplement: Source data 1. — Tuning curves for all neurons scored as significantly tuned and tracked, for each of the three conditions: control, dark exposure (DE), and light reintroduction (LRx). [file elife-80361-data1.zip › SourceData1/b1_b2/2472_1L_cellPairID_28.png]

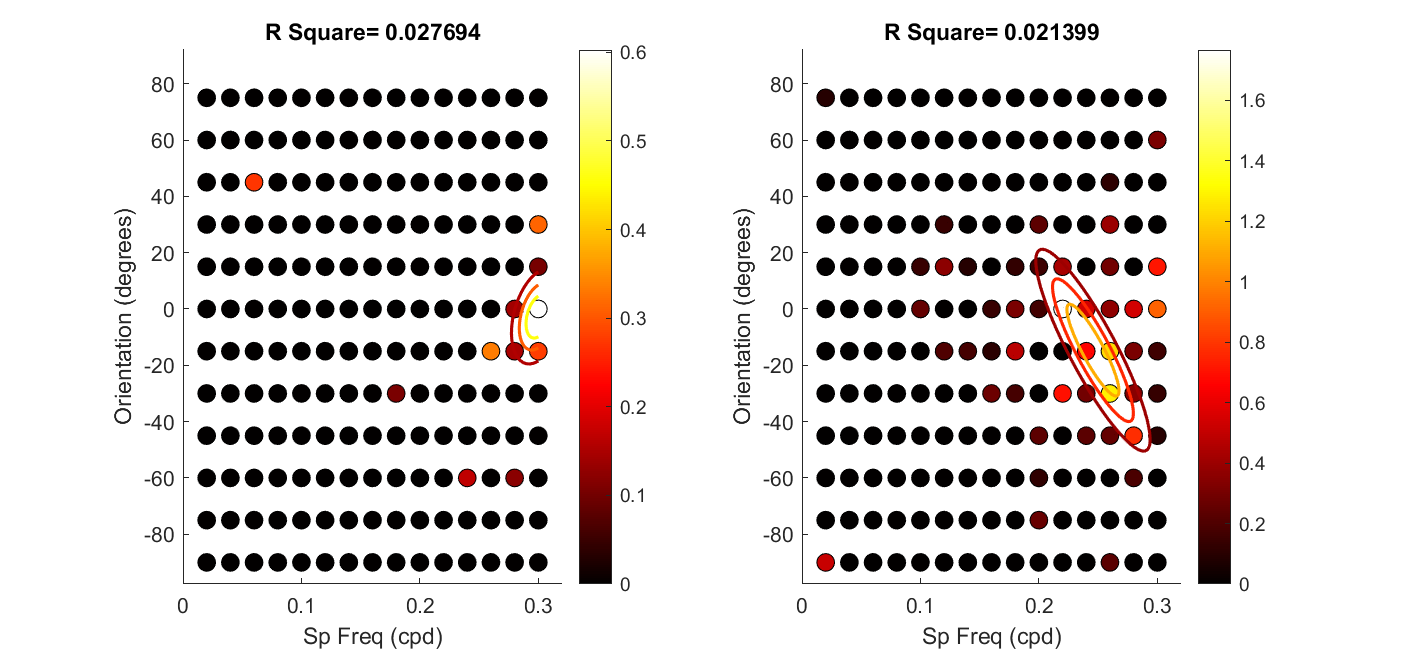

Supplement: Source data 1. — Tuning curves for all neurons scored as significantly tuned and tracked, for each of the three conditions: control, dark exposure (DE), and light reintroduction (LRx). [file elife-80361-data1.zip › SourceData1/b1_b2/2472_1L_cellPairID_29.png]

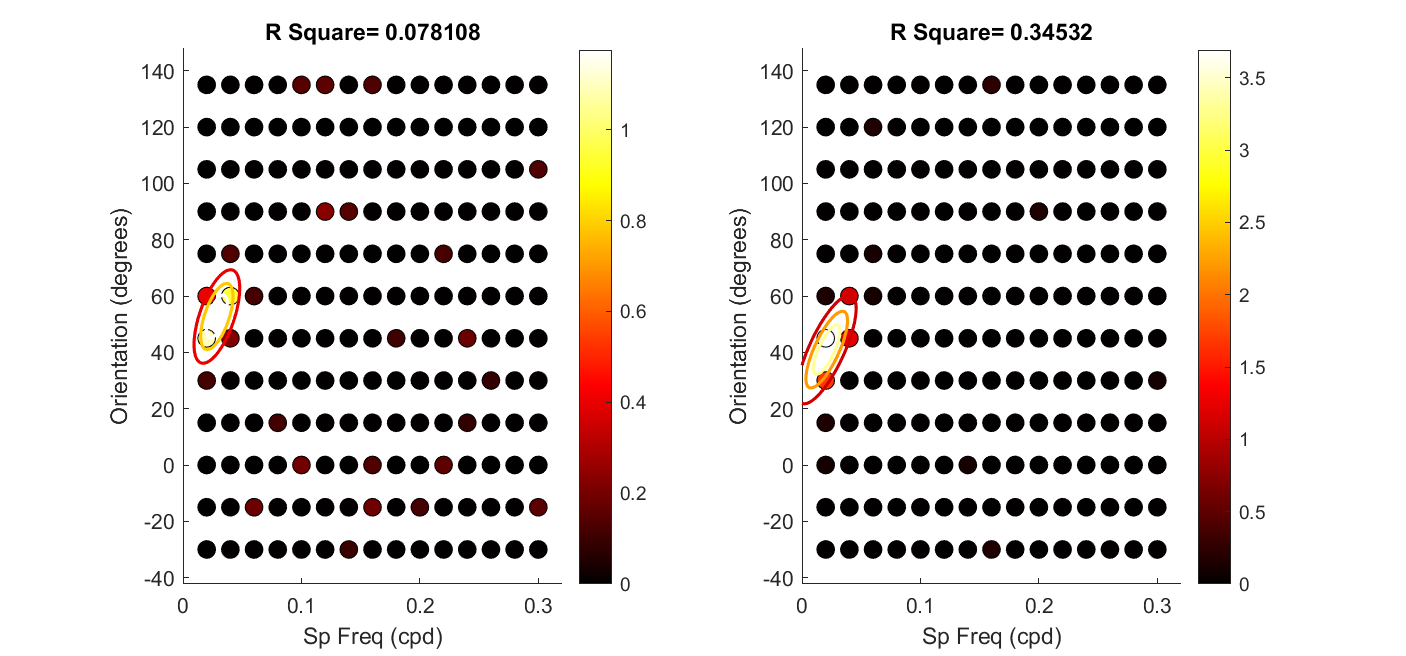

Supplement: Source data 1. — Tuning curves for all neurons scored as significantly tuned and tracked, for each of the three conditions: control, dark exposure (DE), and light reintroduction (LRx). [file elife-80361-data1.zip › SourceData1/b1_b2/2472_1L_cellPairID_3.png]

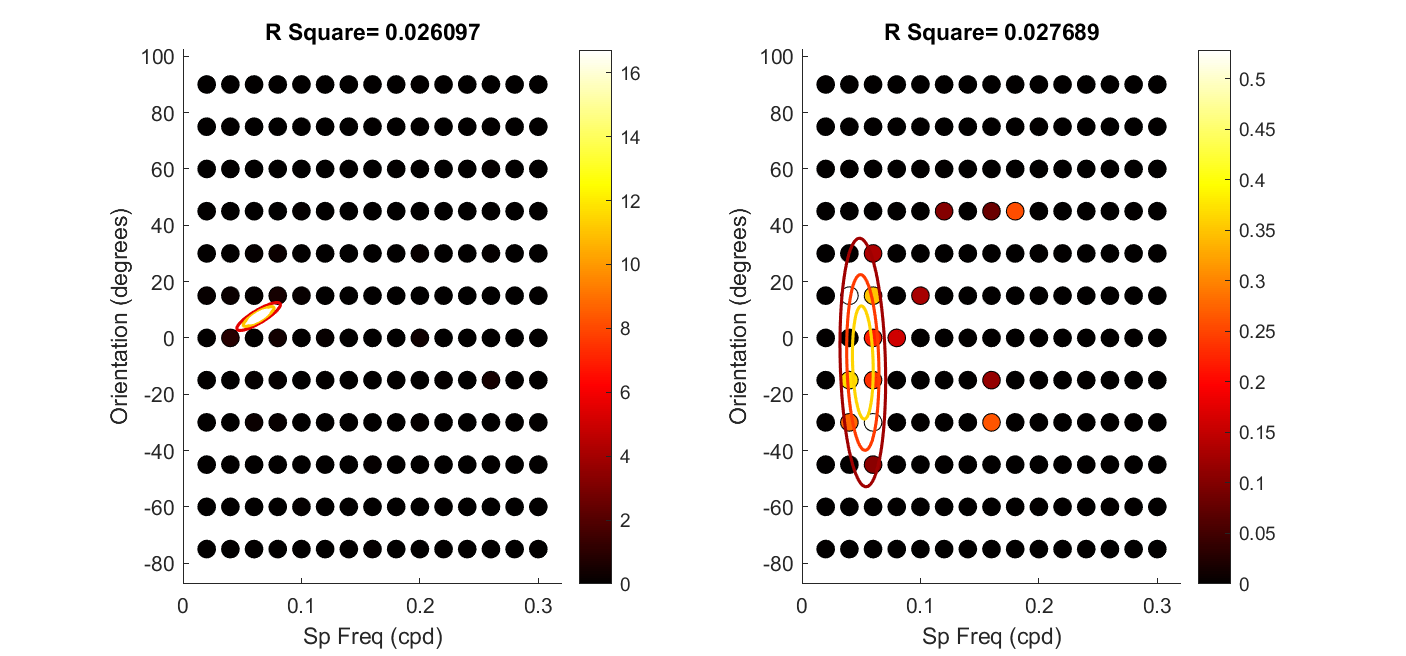

Supplement: Source data 1. — Tuning curves for all neurons scored as significantly tuned and tracked, for each of the three conditions: control, dark exposure (DE), and light reintroduction (LRx). [file elife-80361-data1.zip › SourceData1/b1_b2/2472_1L_cellPairID_30.png]

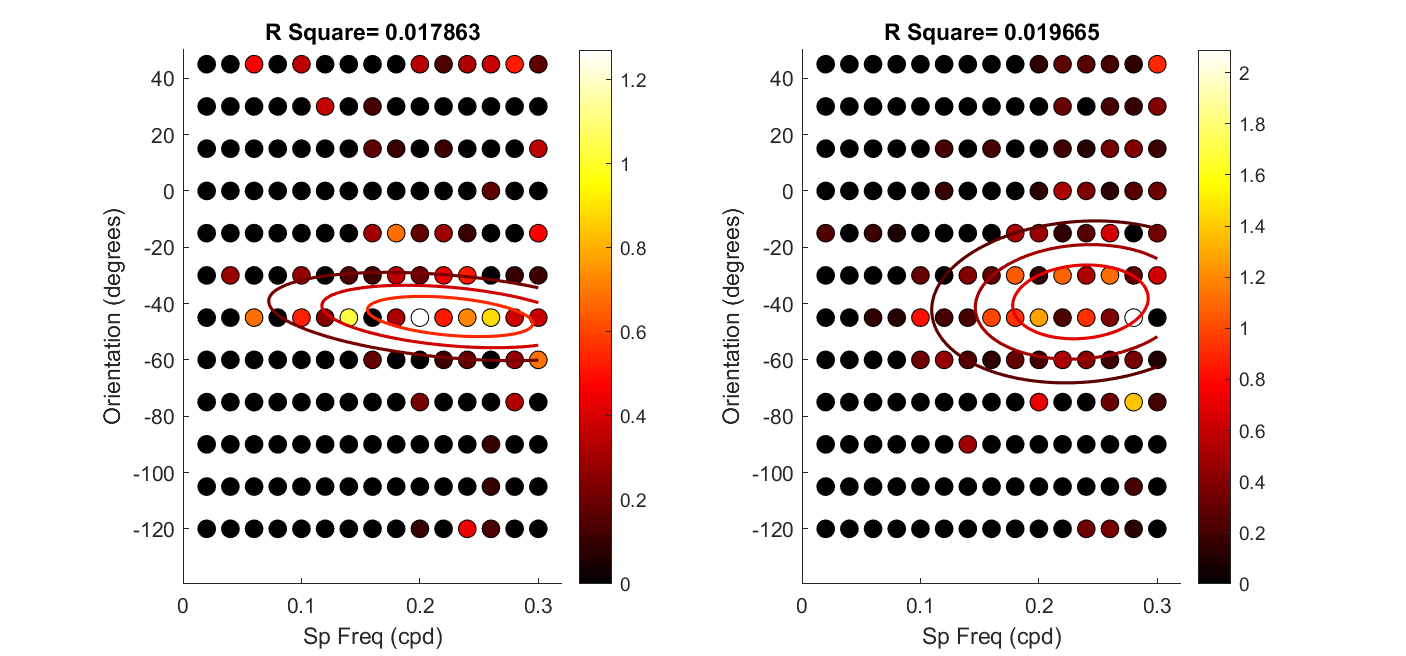

Supplement: Source data 1. — Tuning curves for all neurons scored as significantly tuned and tracked, for each of the three conditions: control, dark exposure (DE), and light reintroduction (LRx). [file elife-80361-data1.zip › SourceData1/b1_b2/2472_1L_cellPairID_31.png]

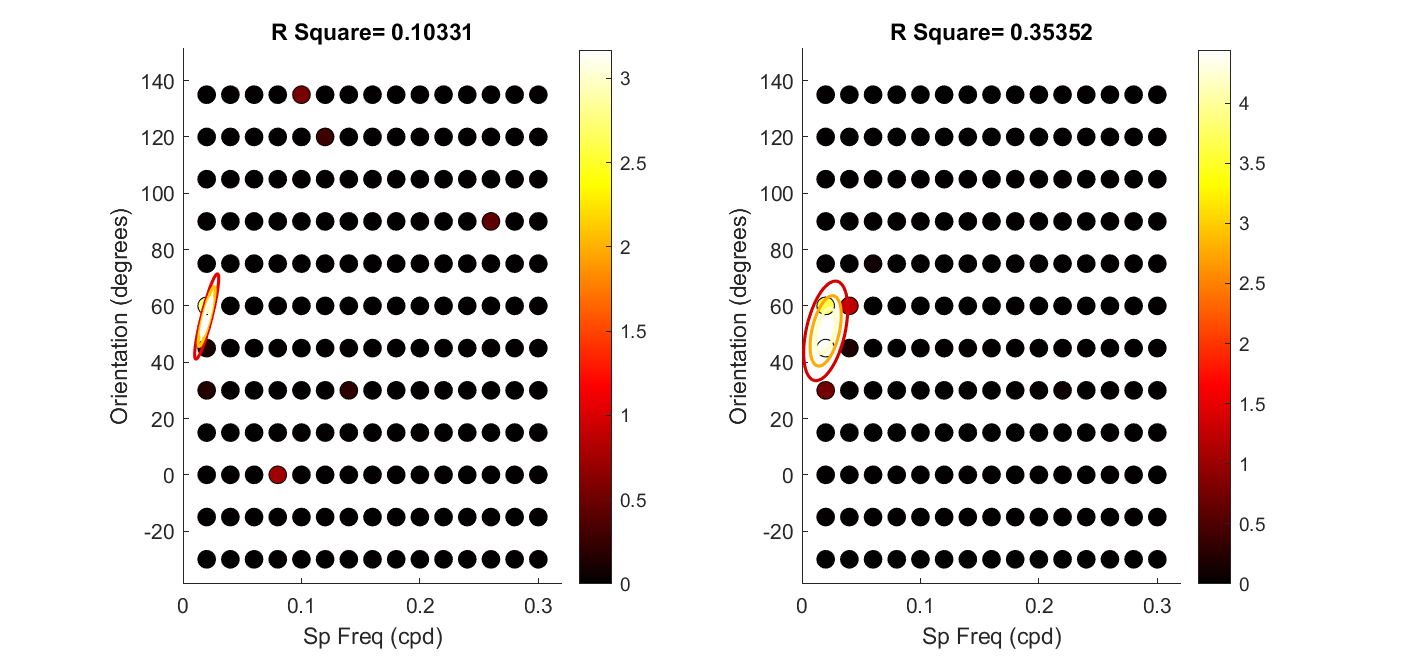

Supplement: Source data 1. — Tuning curves for all neurons scored as significantly tuned and tracked, for each of the three conditions: control, dark exposure (DE), and light reintroduction (LRx). [file elife-80361-data1.zip › SourceData1/b1_b2/2472_1L_cellPairID_32.png]

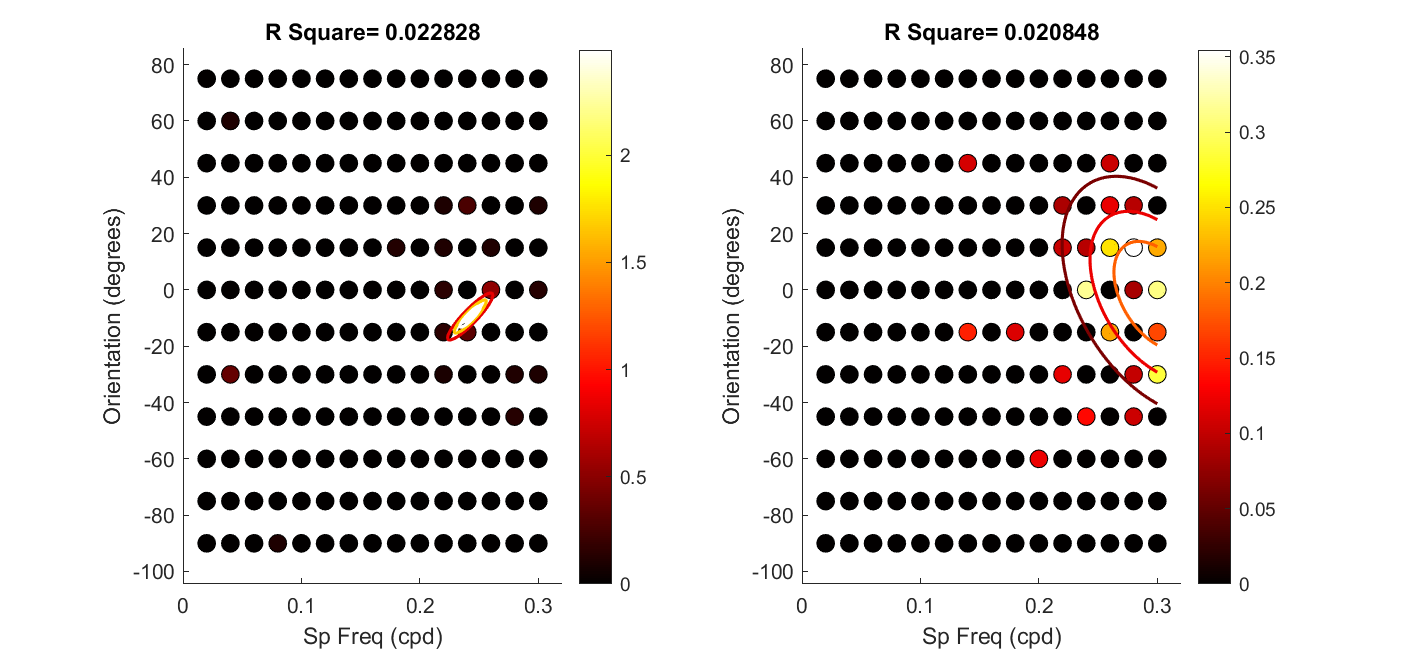

Supplement: Source data 1. — Tuning curves for all neurons scored as significantly tuned and tracked, for each of the three conditions: control, dark exposure (DE), and light reintroduction (LRx). [file elife-80361-data1.zip › SourceData1/b1_b2/2472_1L_cellPairID_33.png]

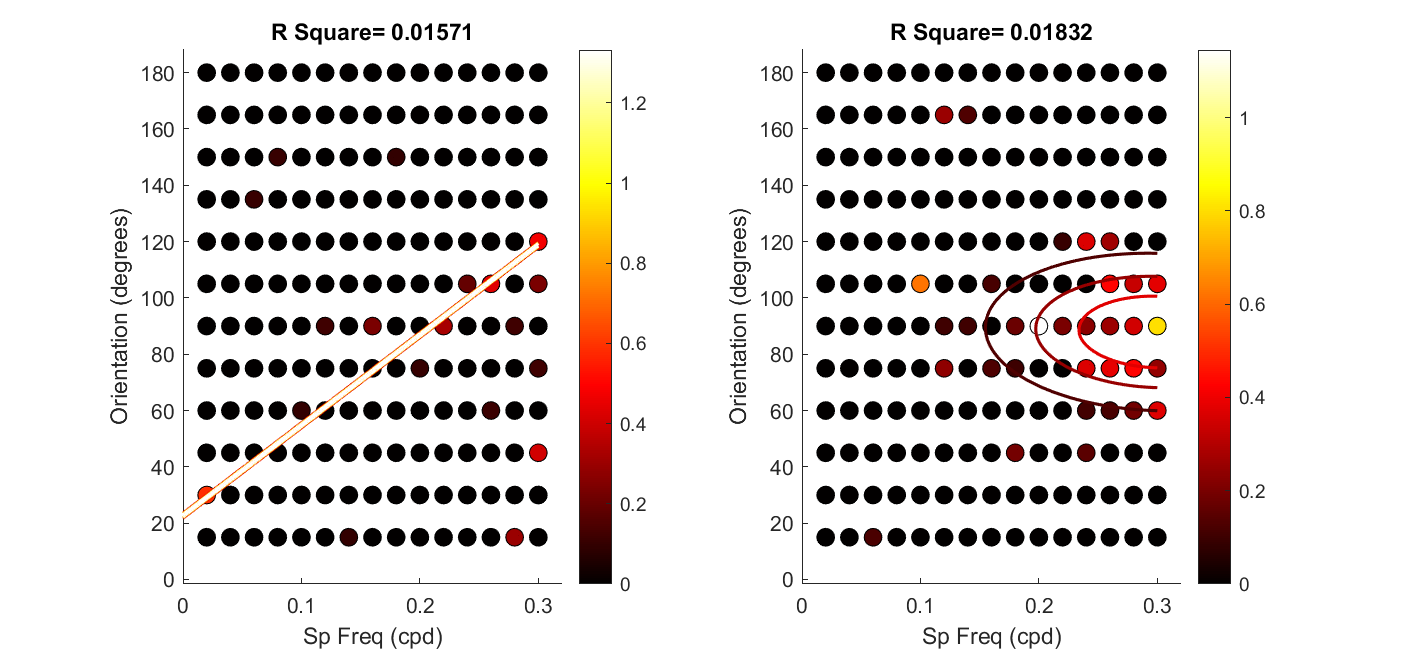

Supplement: Source data 1. — Tuning curves for all neurons scored as significantly tuned and tracked, for each of the three conditions: control, dark exposure (DE), and light reintroduction (LRx). [file elife-80361-data1.zip › SourceData1/b1_b2/2472_1L_cellPairID_34.png]

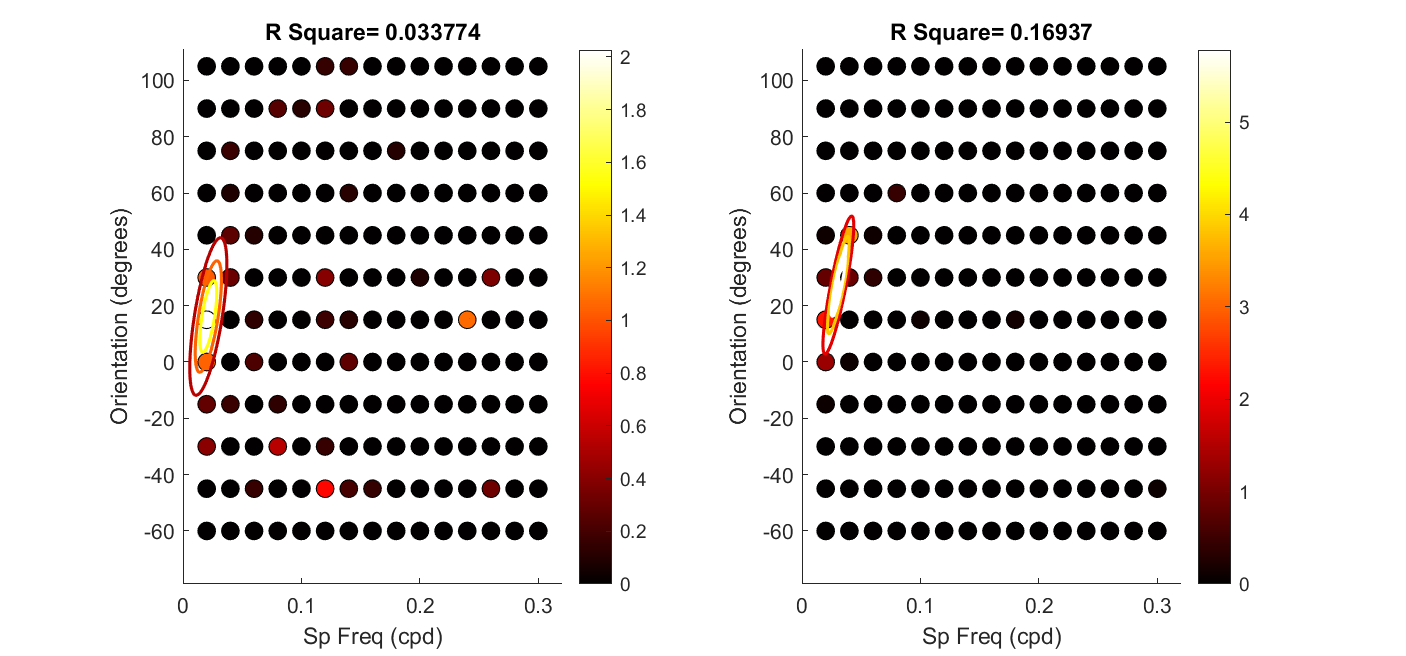

Supplement: Source data 1. — Tuning curves for all neurons scored as significantly tuned and tracked, for each of the three conditions: control, dark exposure (DE), and light reintroduction (LRx). [file elife-80361-data1.zip › SourceData1/b1_b2/2472_1L_cellPairID_35.png]

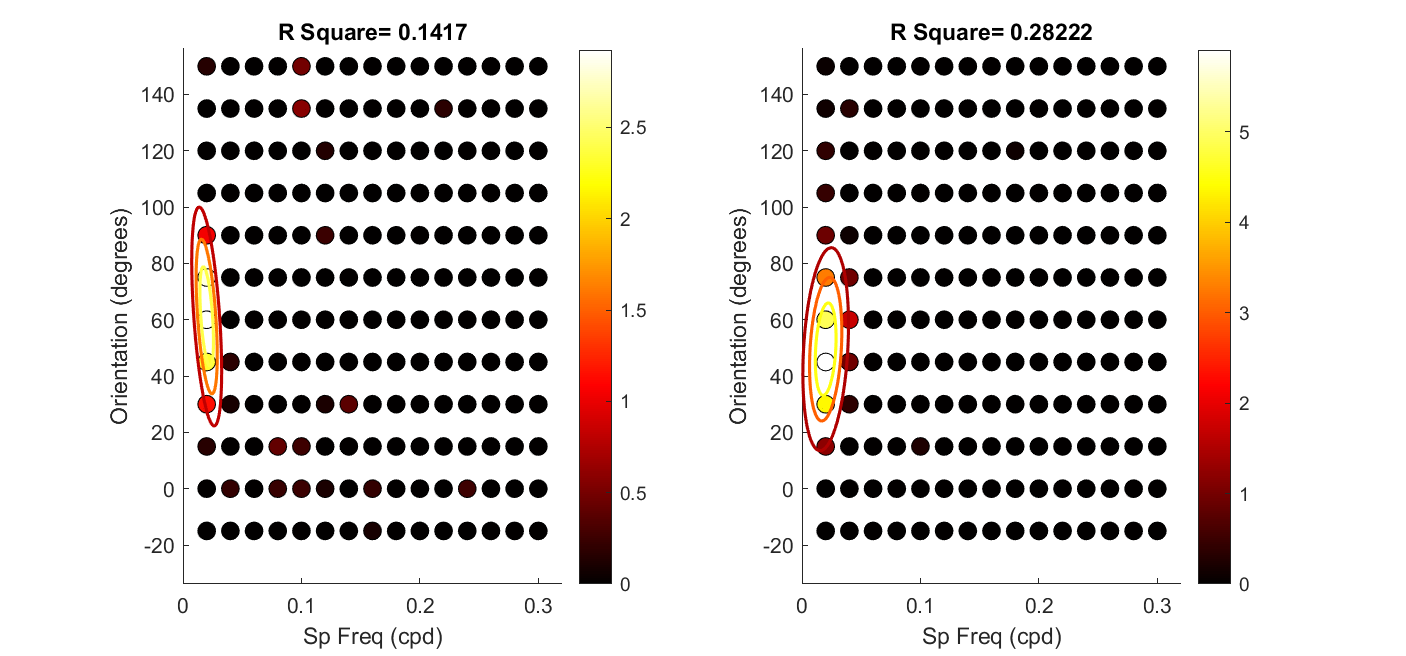

Supplement: Source data 1. — Tuning curves for all neurons scored as significantly tuned and tracked, for each of the three conditions: control, dark exposure (DE), and light reintroduction (LRx). [file elife-80361-data1.zip › SourceData1/b1_b2/2472_1L_cellPairID_36.png]

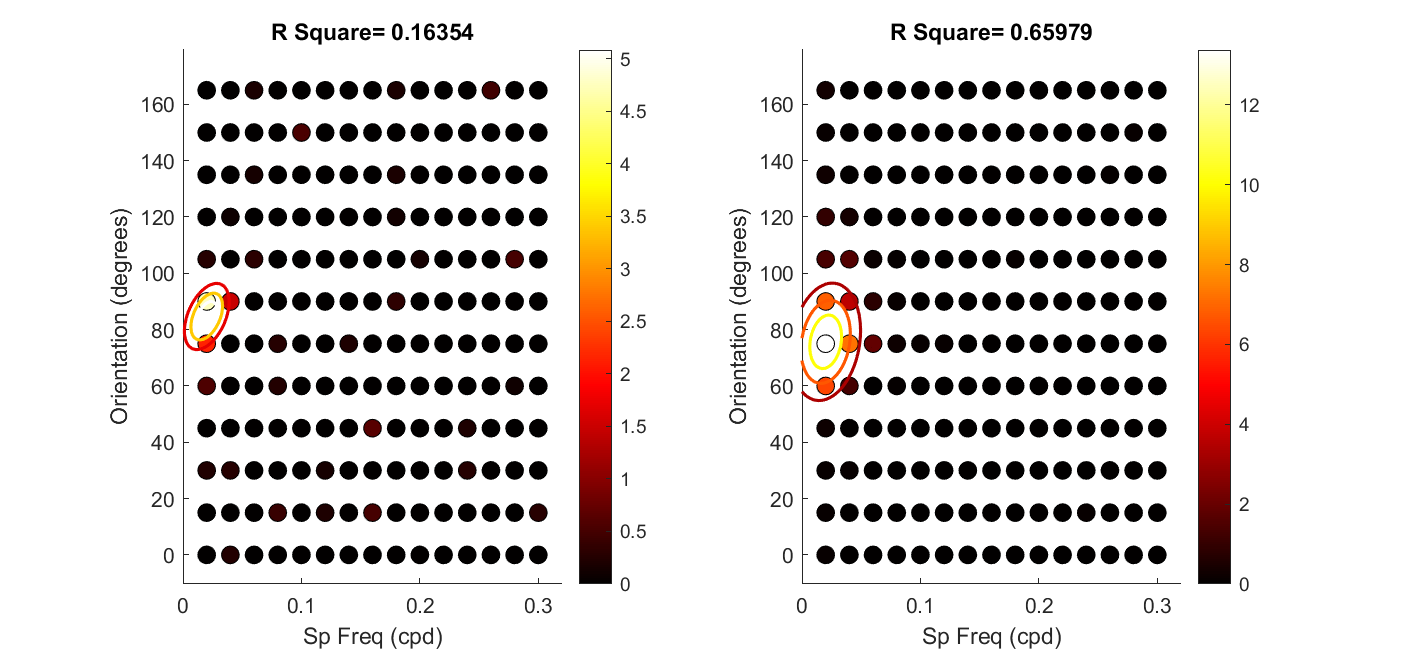

Supplement: Source data 1. — Tuning curves for all neurons scored as significantly tuned and tracked, for each of the three conditions: control, dark exposure (DE), and light reintroduction (LRx). [file elife-80361-data1.zip › SourceData1/b1_b2/2472_1L_cellPairID_37.png]

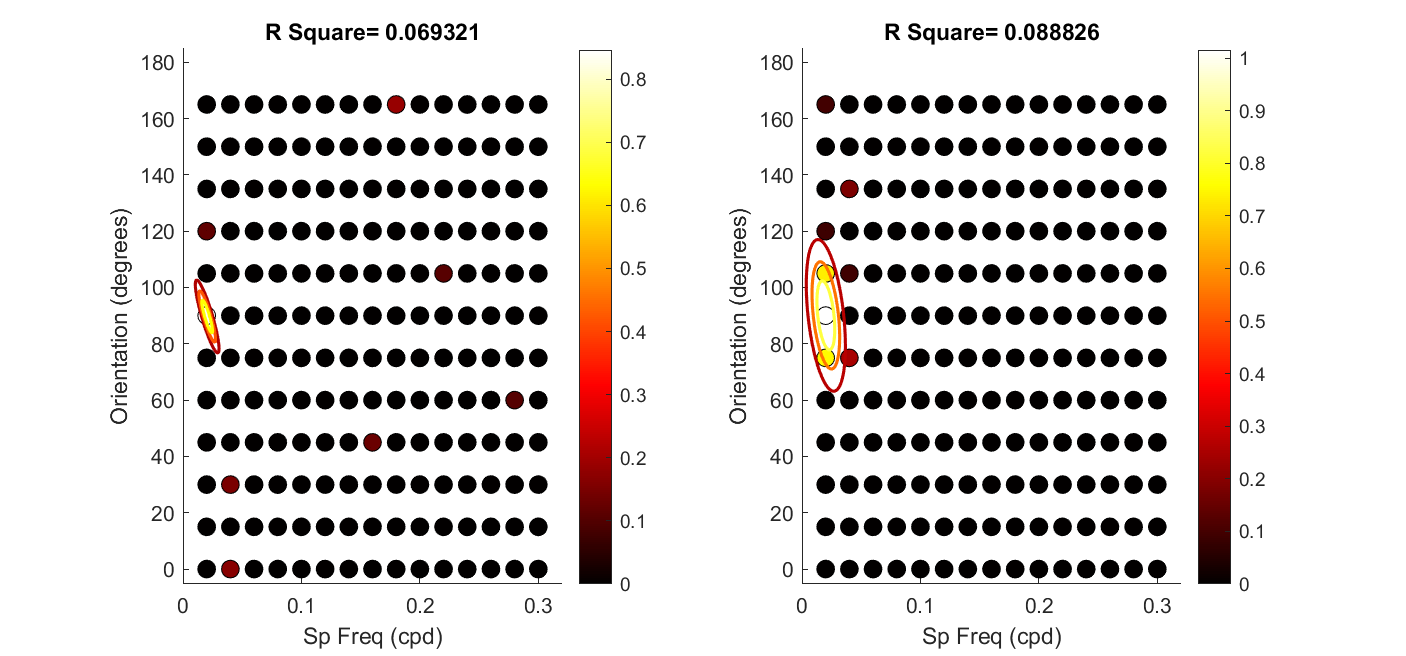

Supplement: Source data 1. — Tuning curves for all neurons scored as significantly tuned and tracked, for each of the three conditions: control, dark exposure (DE), and light reintroduction (LRx). [file elife-80361-data1.zip › SourceData1/b1_b2/2472_1L_cellPairID_38.png]

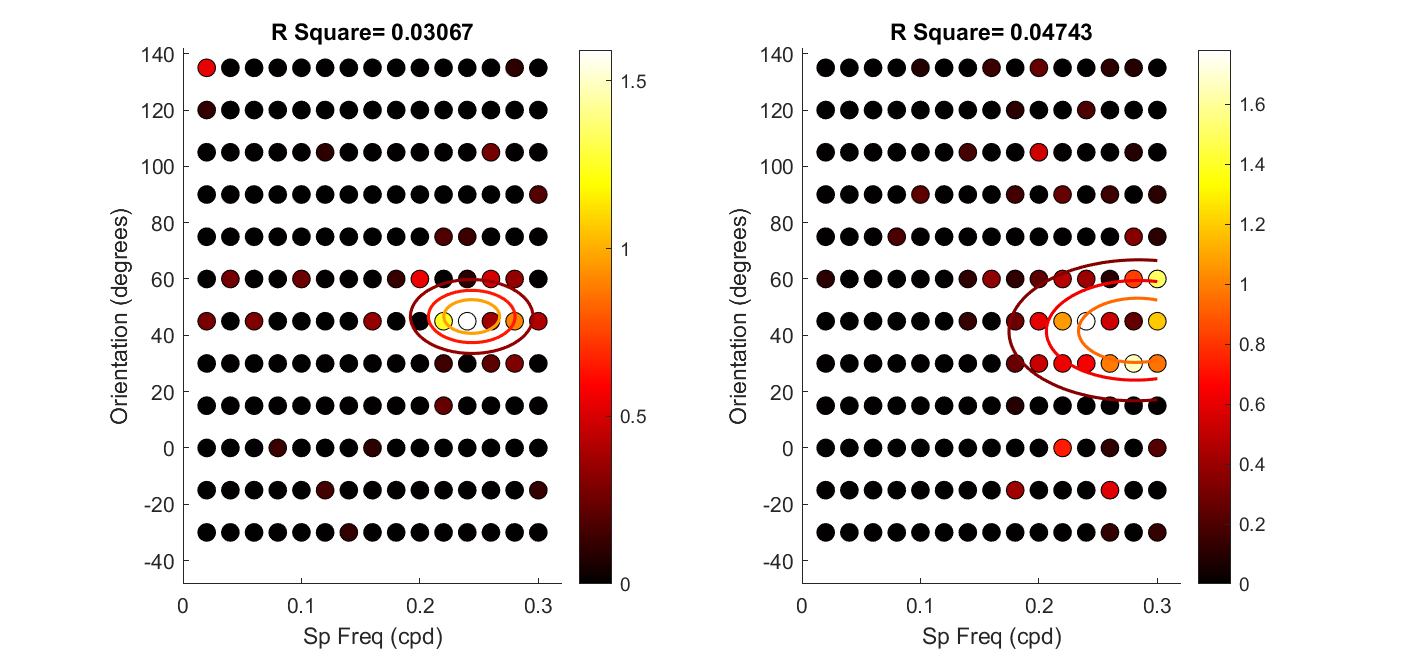

Supplement: Source data 1. — Tuning curves for all neurons scored as significantly tuned and tracked, for each of the three conditions: control, dark exposure (DE), and light reintroduction (LRx). [file elife-80361-data1.zip › SourceData1/b1_b2/2472_1L_cellPairID_4.png]

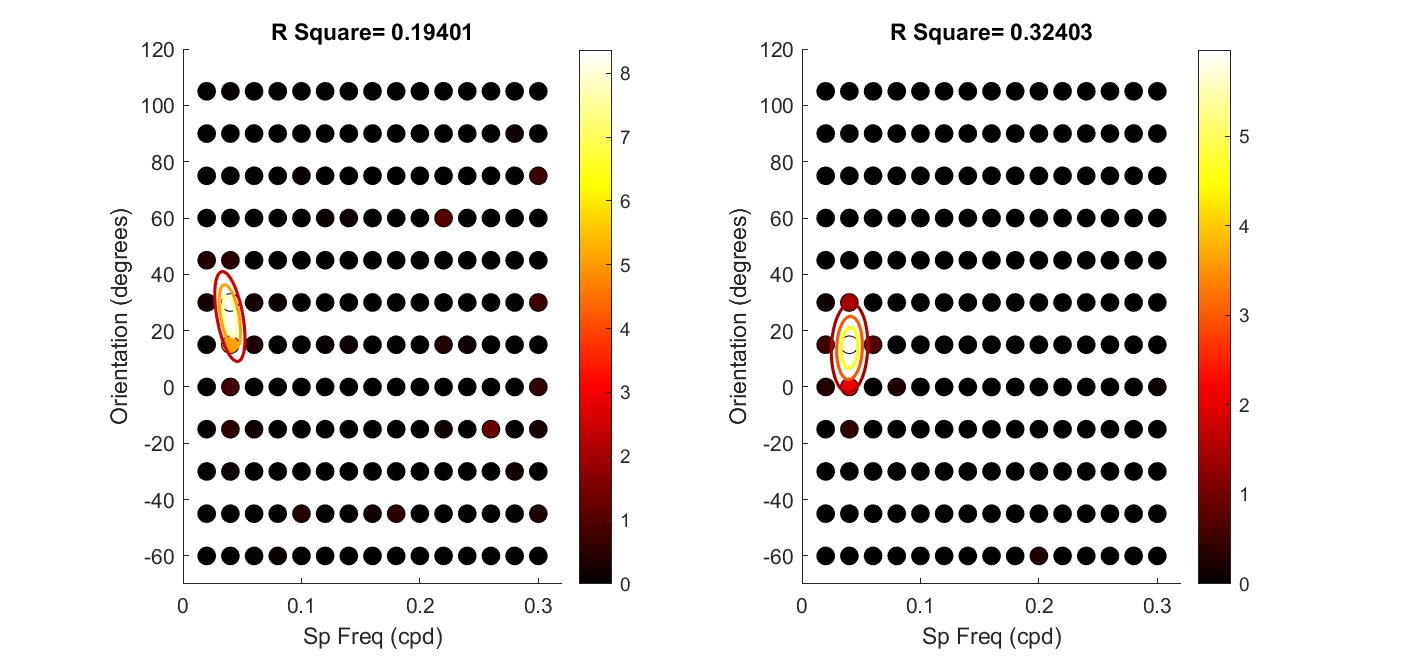

Supplement: Source data 1. — Tuning curves for all neurons scored as significantly tuned and tracked, for each of the three conditions: control, dark exposure (DE), and light reintroduction (LRx). [file elife-80361-data1.zip › SourceData1/b1_b2/2472_1L_cellPairID_5.png]

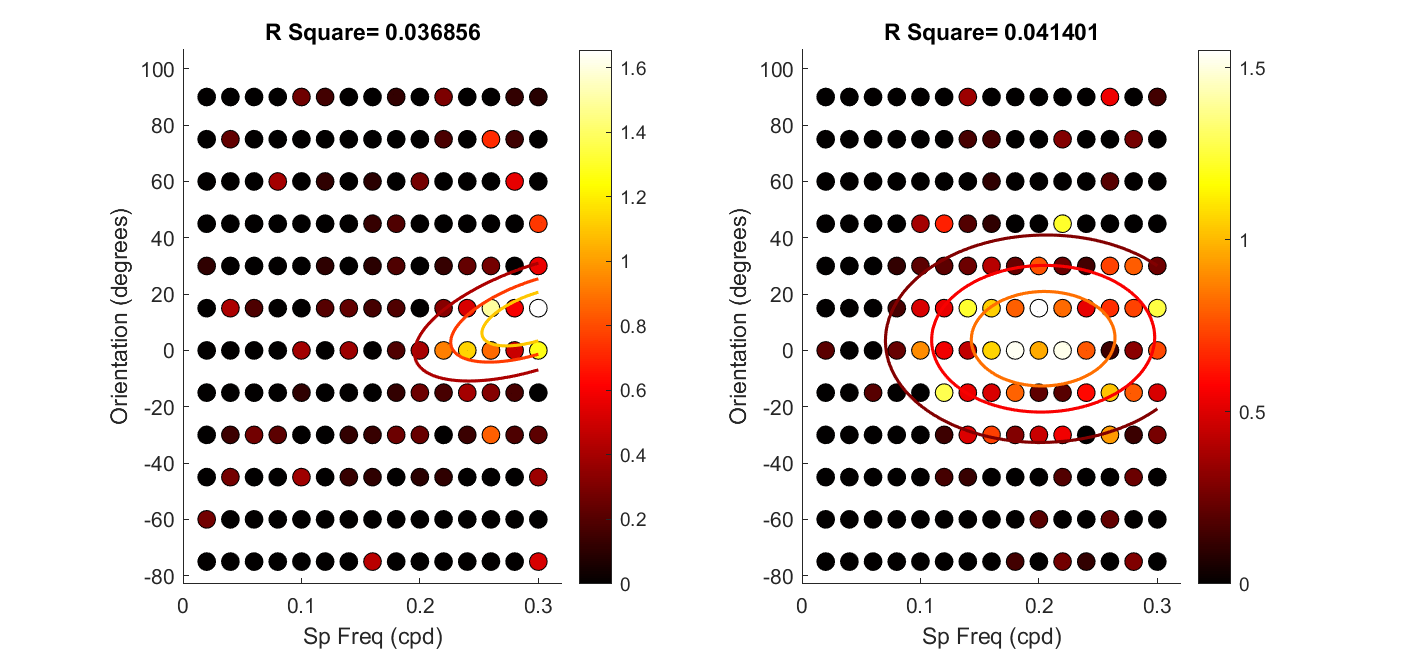

Supplement: Source data 1. — Tuning curves for all neurons scored as significantly tuned and tracked, for each of the three conditions: control, dark exposure (DE), and light reintroduction (LRx). [file elife-80361-data1.zip › SourceData1/b1_b2/2472_1L_cellPairID_6.png]

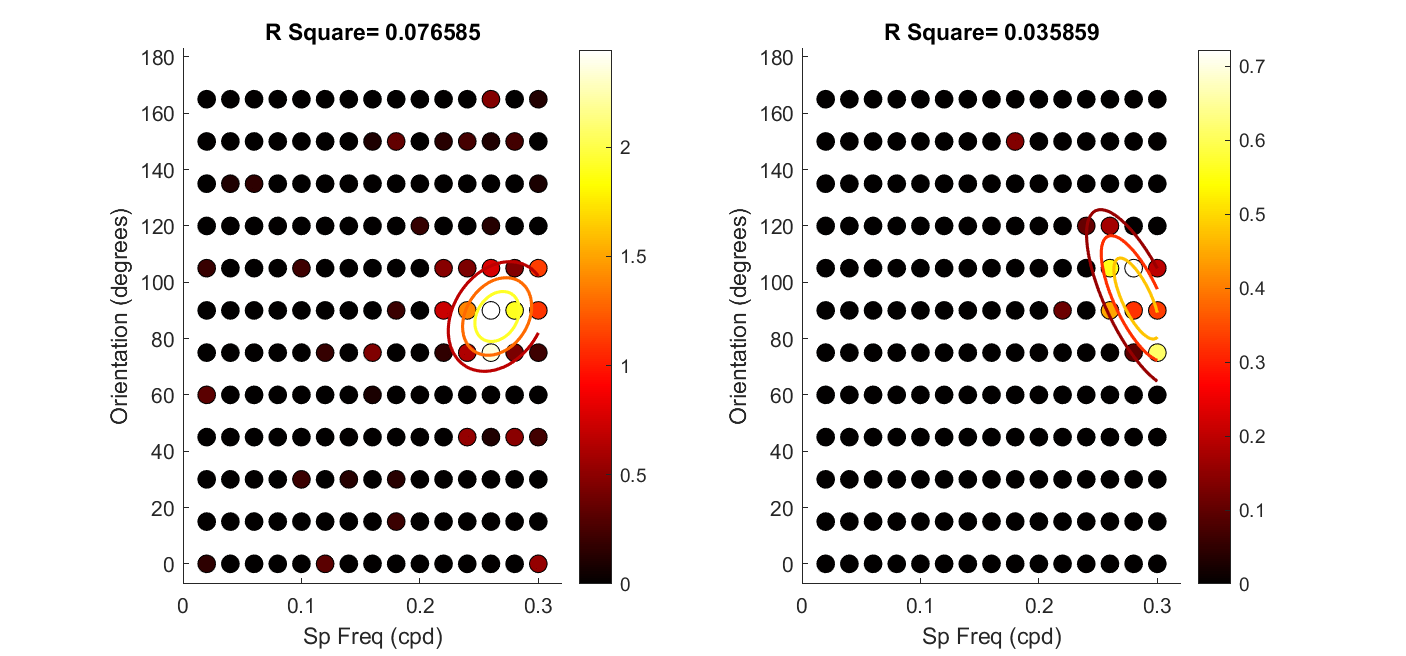

Supplement: Source data 1. — Tuning curves for all neurons scored as significantly tuned and tracked, for each of the three conditions: control, dark exposure (DE), and light reintroduction (LRx). [file elife-80361-data1.zip › SourceData1/b1_b2/2472_1L_cellPairID_7.png]

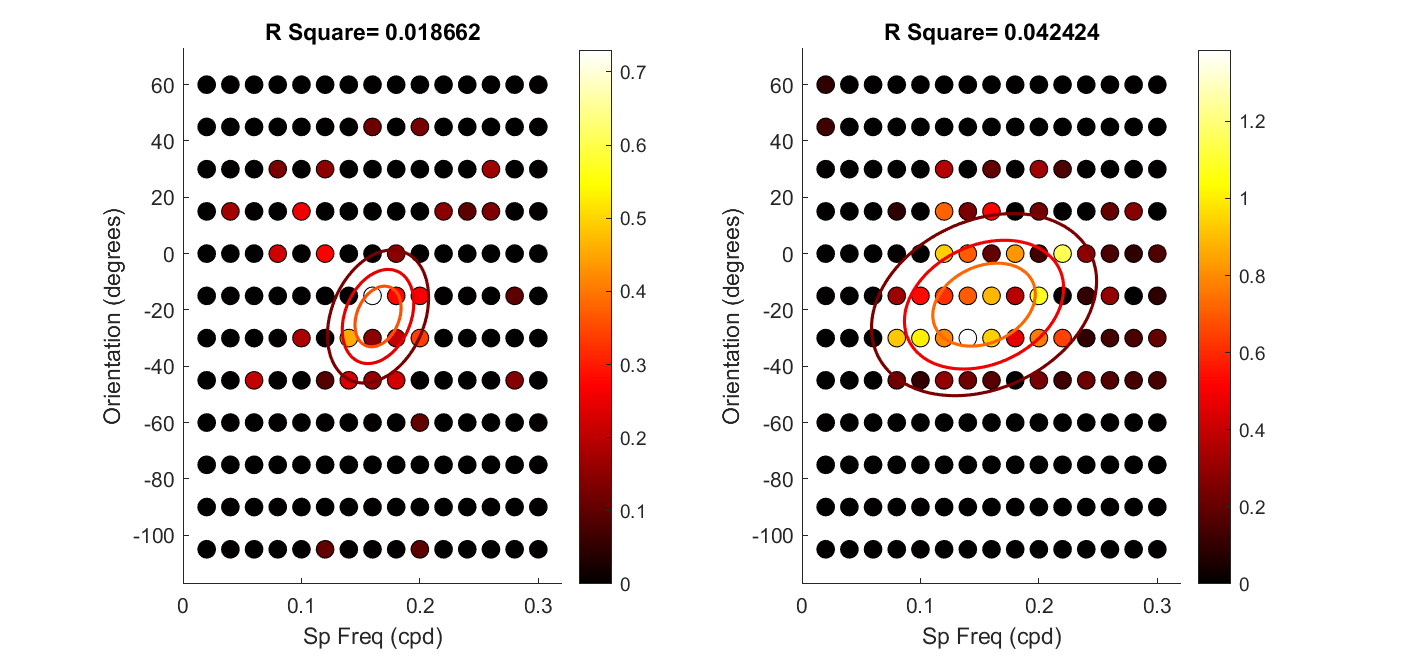

Supplement: Source data 1. — Tuning curves for all neurons scored as significantly tuned and tracked, for each of the three conditions: control, dark exposure (DE), and light reintroduction (LRx). [file elife-80361-data1.zip › SourceData1/b1_b2/2472_1L_cellPairID_8.png]

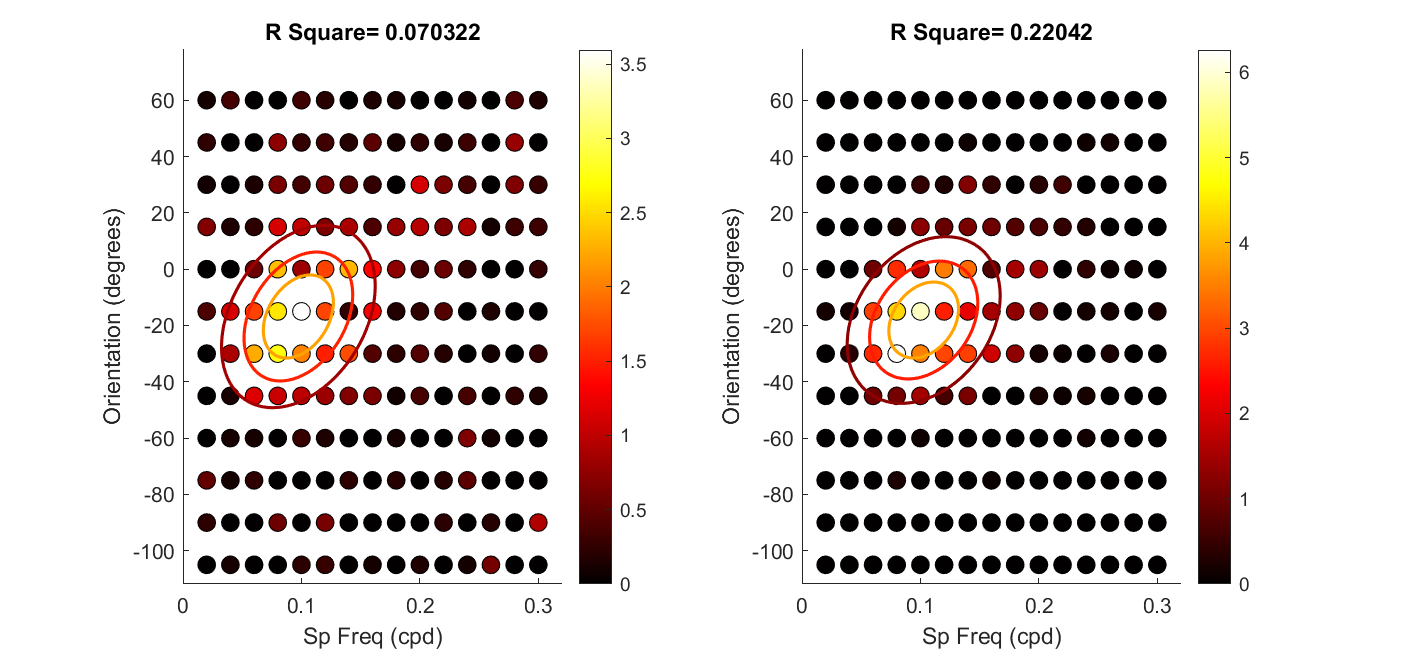

Supplement: Source data 1. — Tuning curves for all neurons scored as significantly tuned and tracked, for each of the three conditions: control, dark exposure (DE), and light reintroduction (LRx). [file elife-80361-data1.zip › SourceData1/b1_b2/2472_1L_cellPairID_9.png]

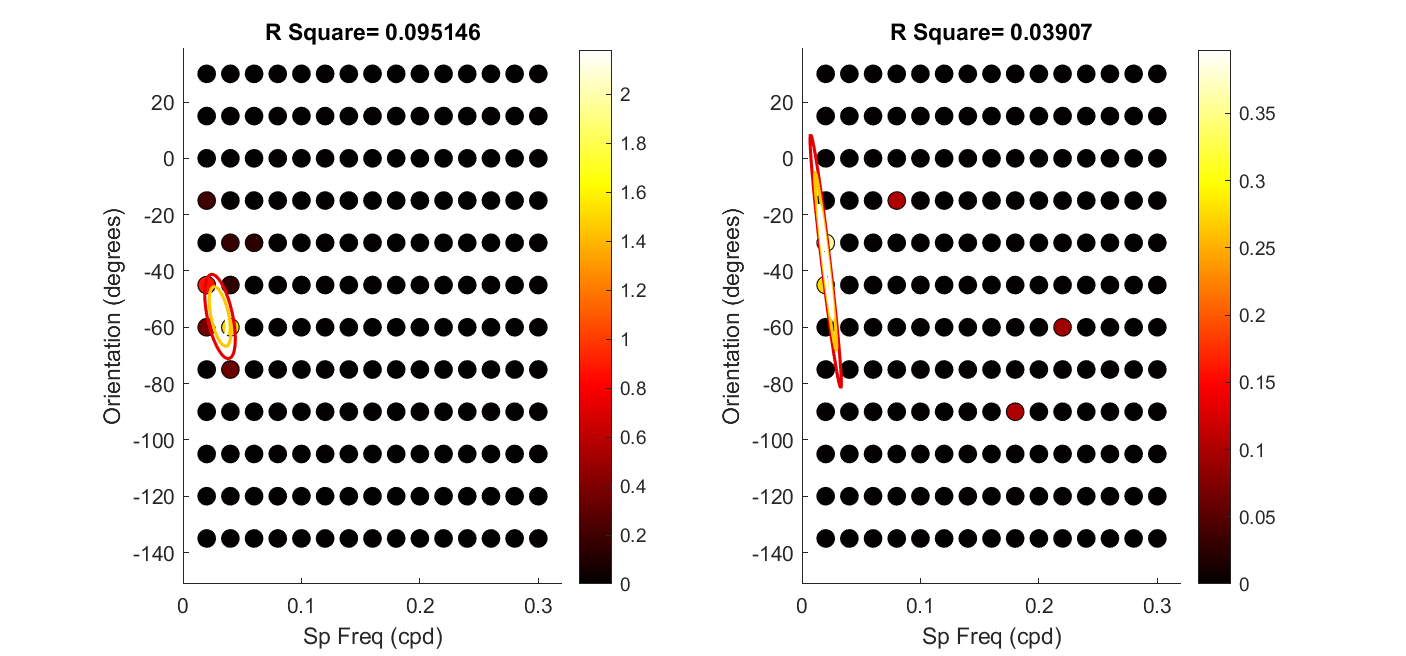

Supplement: Source data 1. — Tuning curves for all neurons scored as significantly tuned and tracked, for each of the three conditions: control, dark exposure (DE), and light reintroduction (LRx). [file elife-80361-data1.zip › SourceData1/b1_b2/2473_1R_cellPairID_1.png]

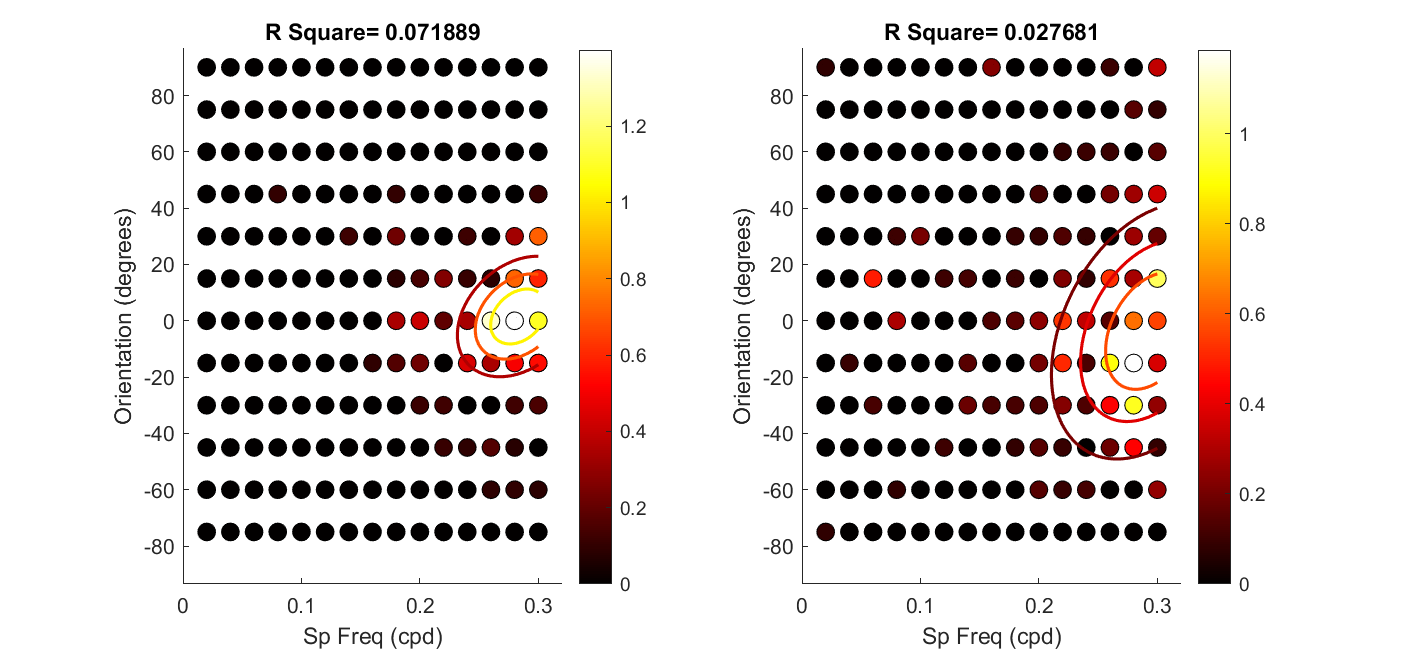

Supplement: Source data 1. — Tuning curves for all neurons scored as significantly tuned and tracked, for each of the three conditions: control, dark exposure (DE), and light reintroduction (LRx). [file elife-80361-data1.zip › SourceData1/b1_b2/2473_1R_cellPairID_10.png]

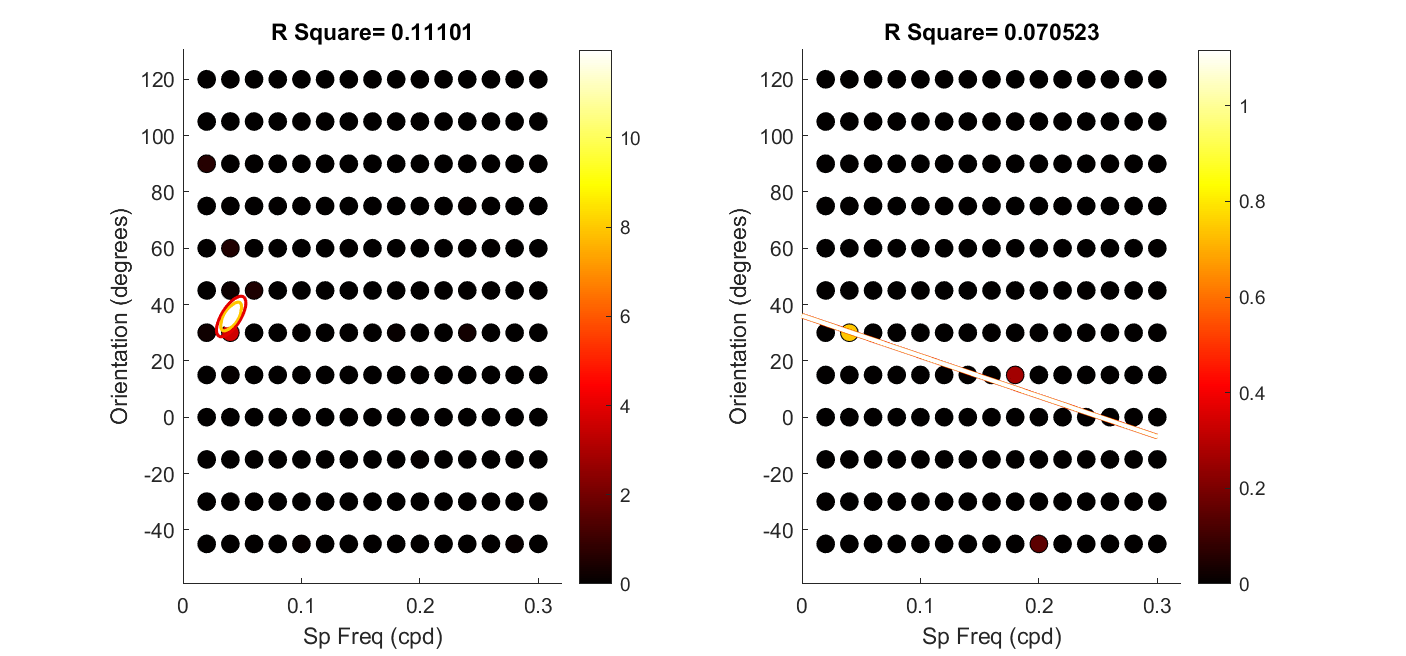

Supplement: Source data 1. — Tuning curves for all neurons scored as significantly tuned and tracked, for each of the three conditions: control, dark exposure (DE), and light reintroduction (LRx). [file elife-80361-data1.zip › SourceData1/b1_b2/2473_1R_cellPairID_11.png]

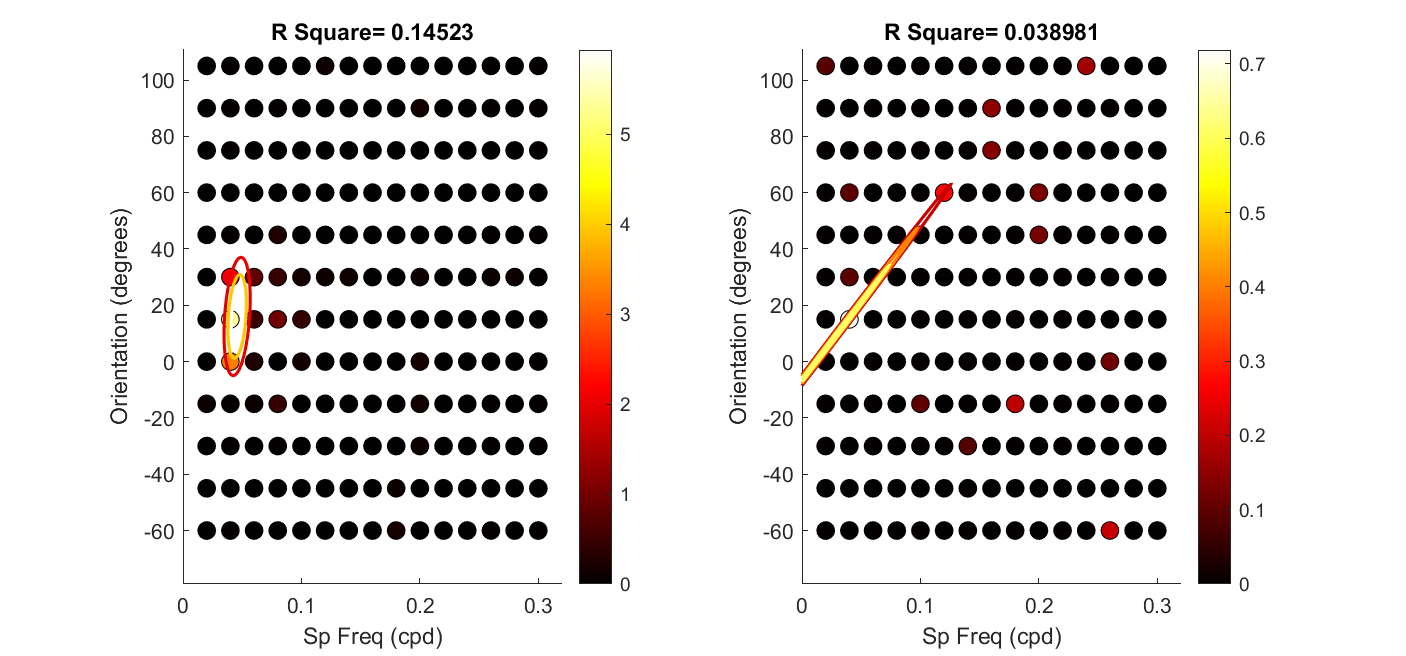

Supplement: Source data 1. — Tuning curves for all neurons scored as significantly tuned and tracked, for each of the three conditions: control, dark exposure (DE), and light reintroduction (LRx). [file elife-80361-data1.zip › SourceData1/b1_b2/2473_1R_cellPairID_12.png]

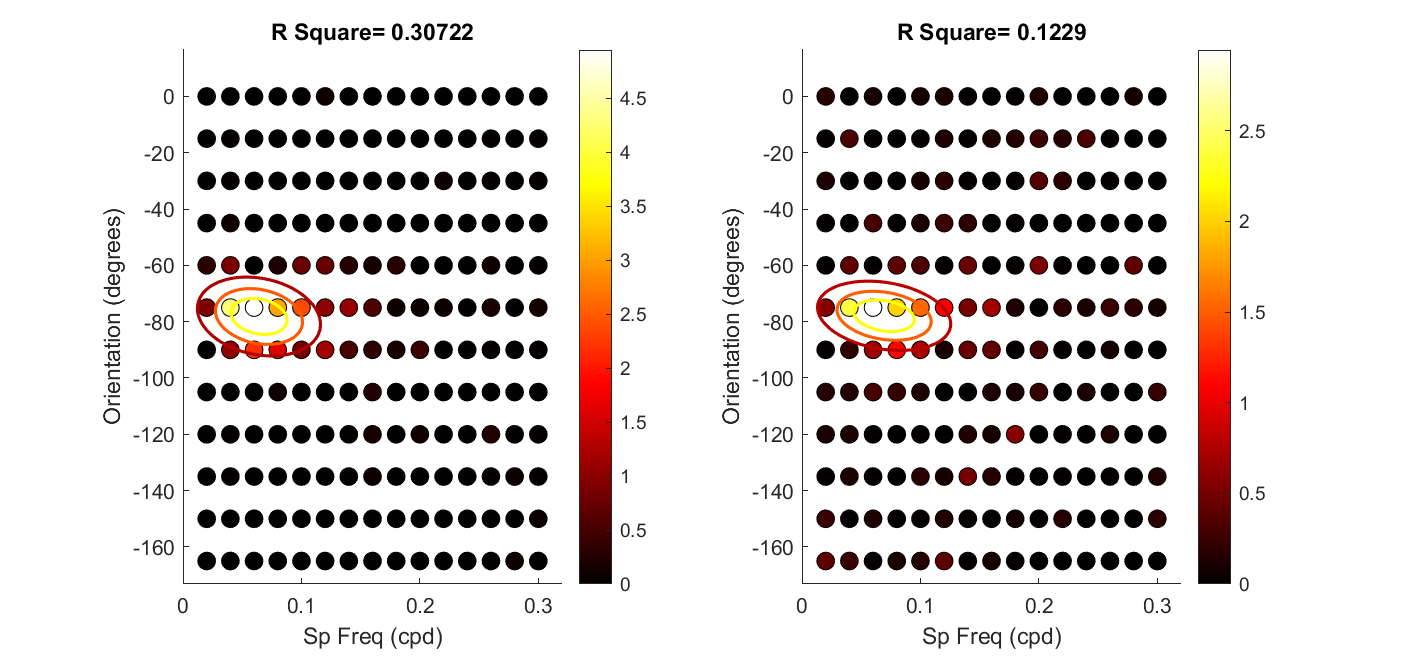

Supplement: Source data 1. — Tuning curves for all neurons scored as significantly tuned and tracked, for each of the three conditions: control, dark exposure (DE), and light reintroduction (LRx). [file elife-80361-data1.zip › SourceData1/b1_b2/2473_1R_cellPairID_13.png]

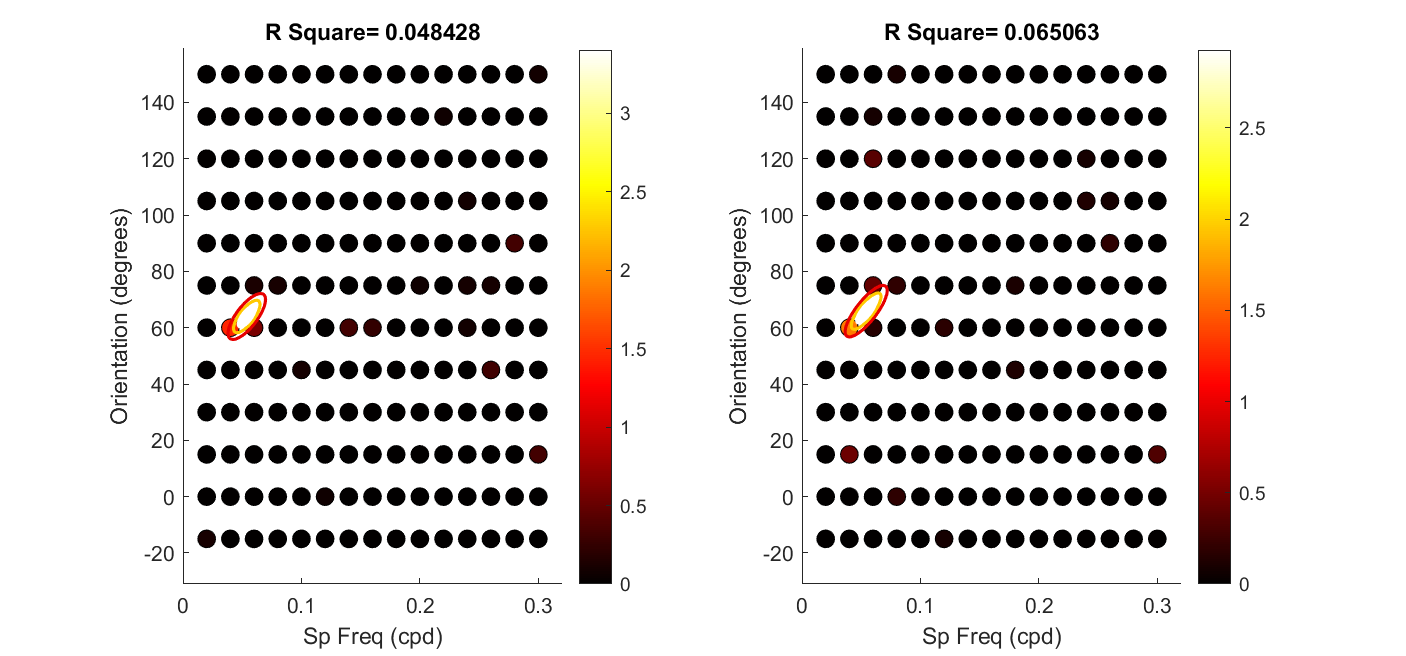

Supplement: Source data 1. — Tuning curves for all neurons scored as significantly tuned and tracked, for each of the three conditions: control, dark exposure (DE), and light reintroduction (LRx). [file elife-80361-data1.zip › SourceData1/b1_b2/2473_1R_cellPairID_14.png]

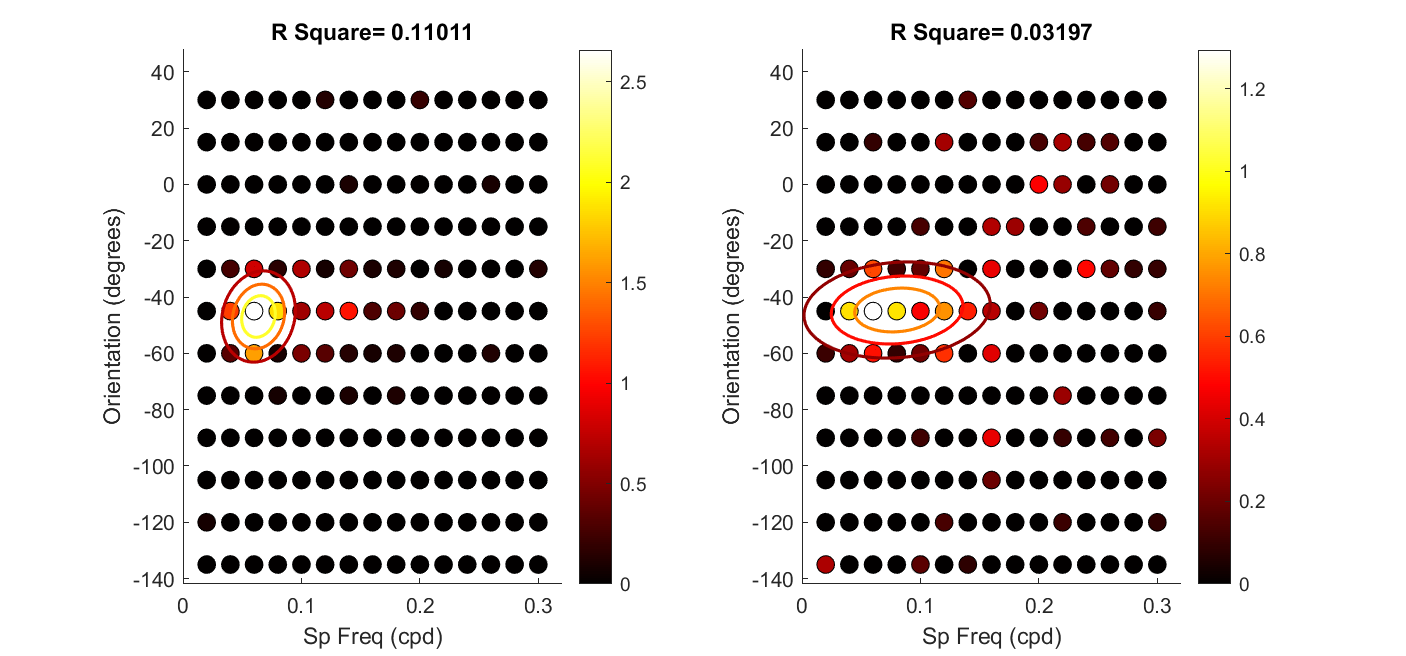

Supplement: Source data 1. — Tuning curves for all neurons scored as significantly tuned and tracked, for each of the three conditions: control, dark exposure (DE), and light reintroduction (LRx). [file elife-80361-data1.zip › SourceData1/b1_b2/2473_1R_cellPairID_15.png]

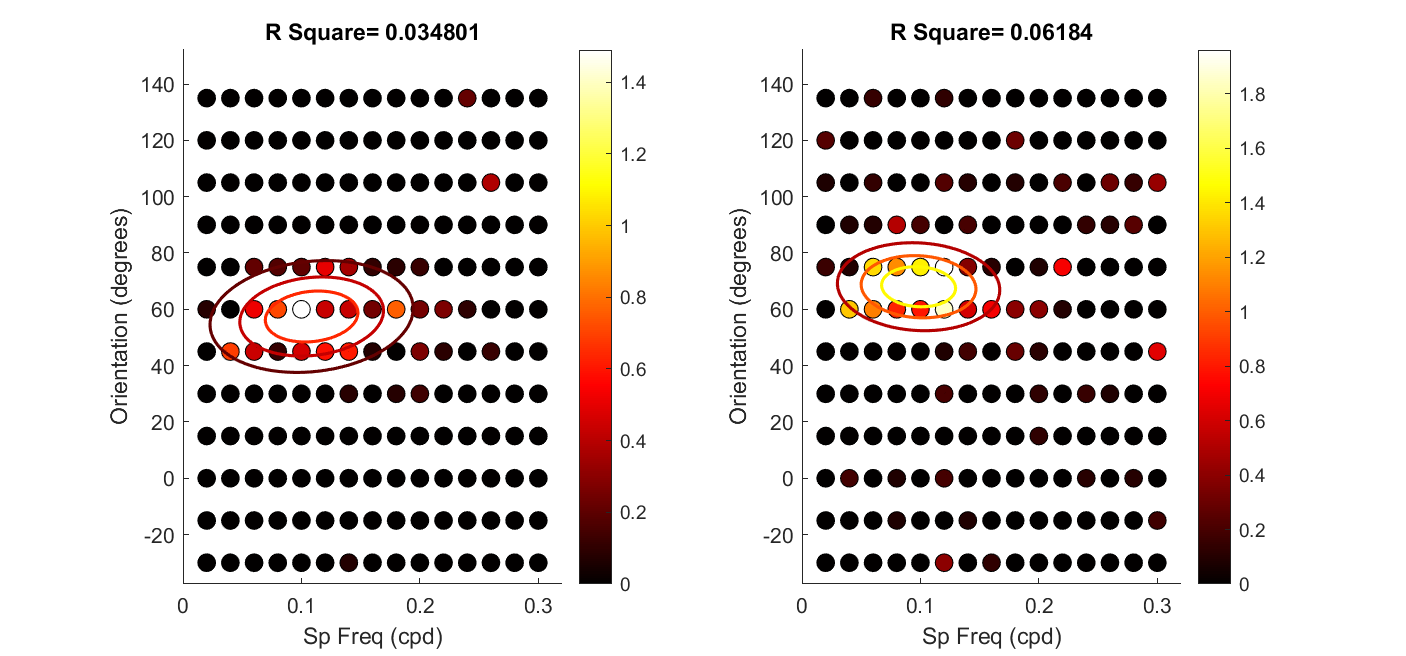

Supplement: Source data 1. — Tuning curves for all neurons scored as significantly tuned and tracked, for each of the three conditions: control, dark exposure (DE), and light reintroduction (LRx). [file elife-80361-data1.zip › SourceData1/b1_b2/2473_1R_cellPairID_16.png]

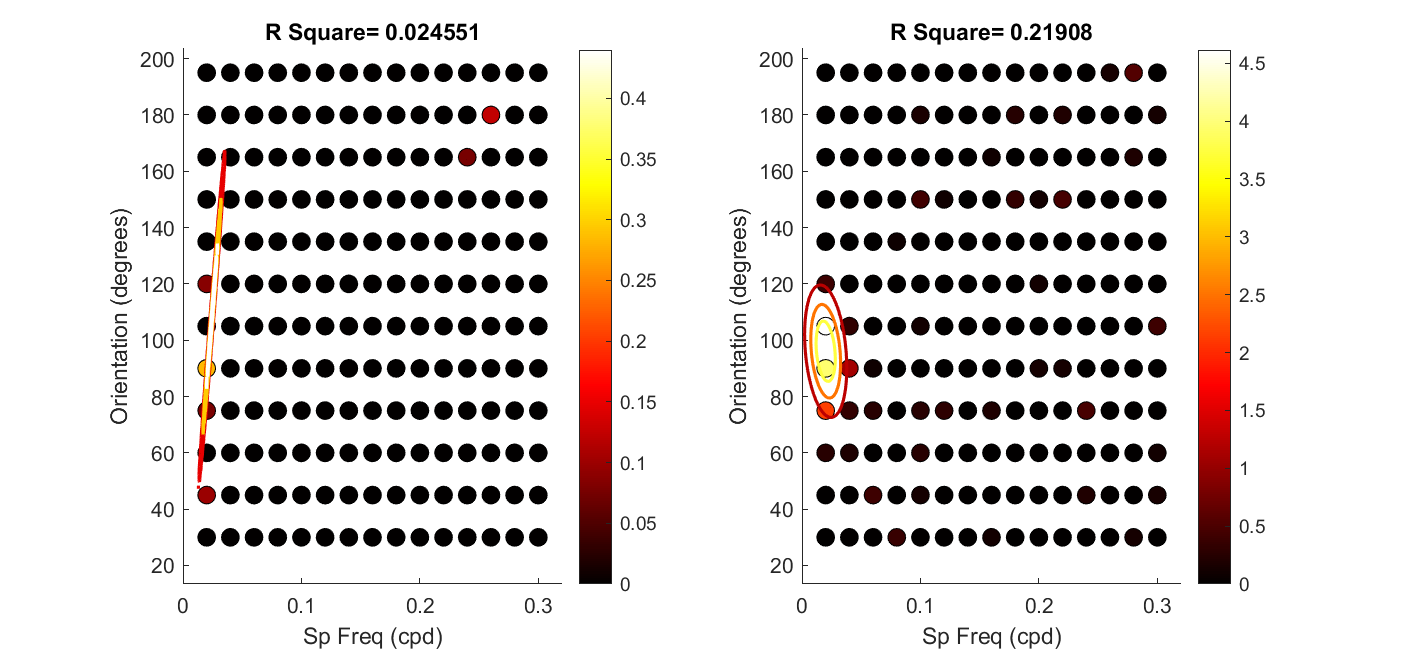

Supplement: Source data 1. — Tuning curves for all neurons scored as significantly tuned and tracked, for each of the three conditions: control, dark exposure (DE), and light reintroduction (LRx). [file elife-80361-data1.zip › SourceData1/b1_b2/2473_1R_cellPairID_17.png]

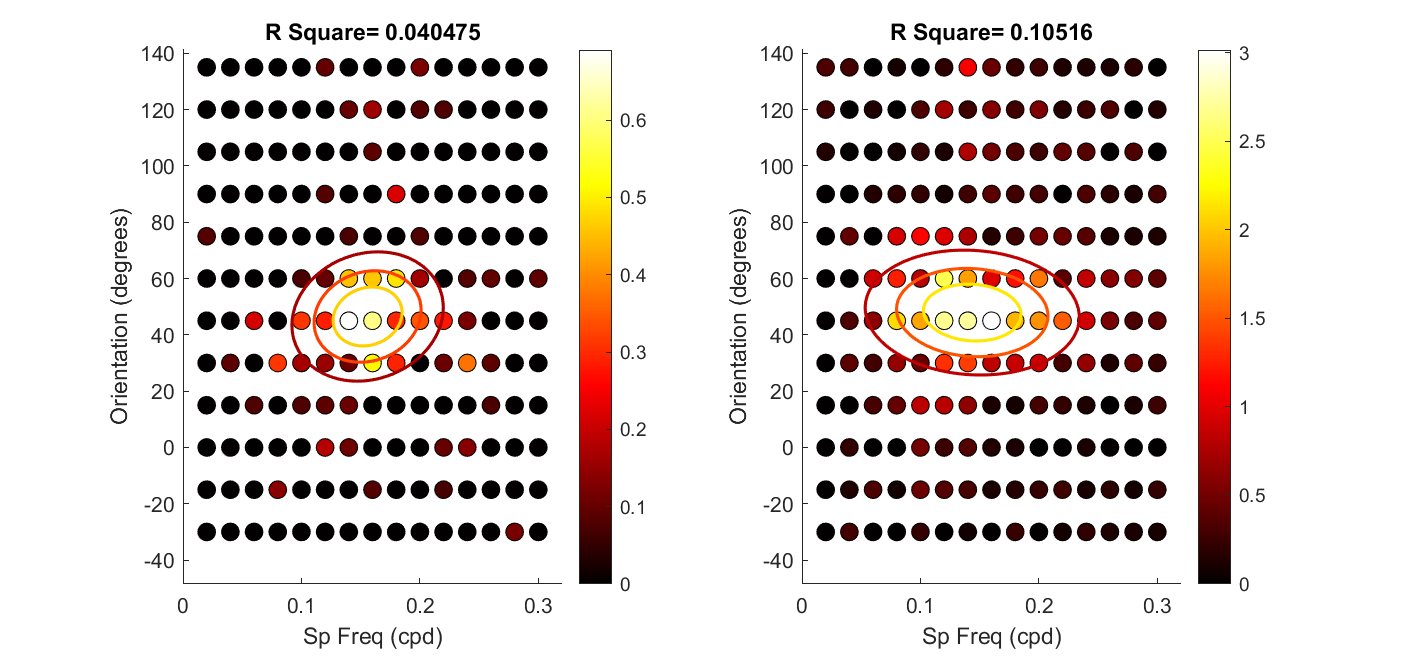

Supplement: Source data 1. — Tuning curves for all neurons scored as significantly tuned and tracked, for each of the three conditions: control, dark exposure (DE), and light reintroduction (LRx). [file elife-80361-data1.zip › SourceData1/b1_b2/2473_1R_cellPairID_18.png]

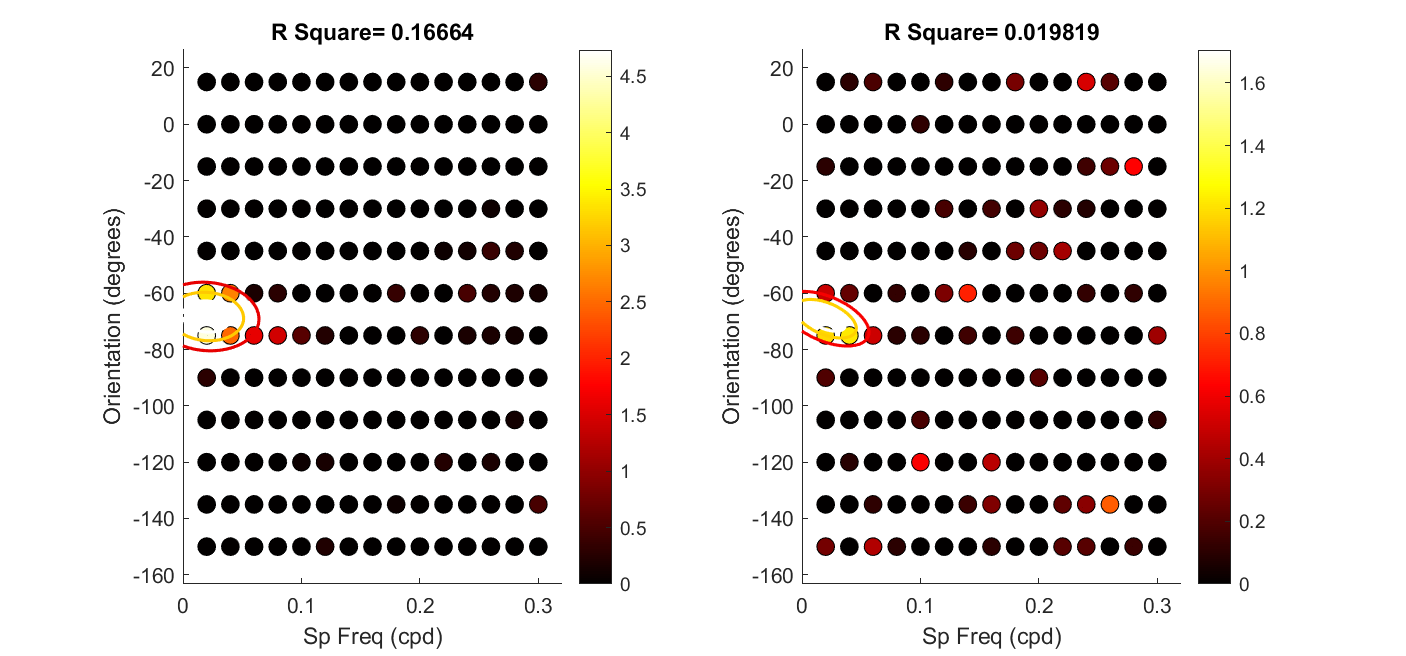

Supplement: Source data 1. — Tuning curves for all neurons scored as significantly tuned and tracked, for each of the three conditions: control, dark exposure (DE), and light reintroduction (LRx). [file elife-80361-data1.zip › SourceData1/b1_b2/2473_1R_cellPairID_19.png]

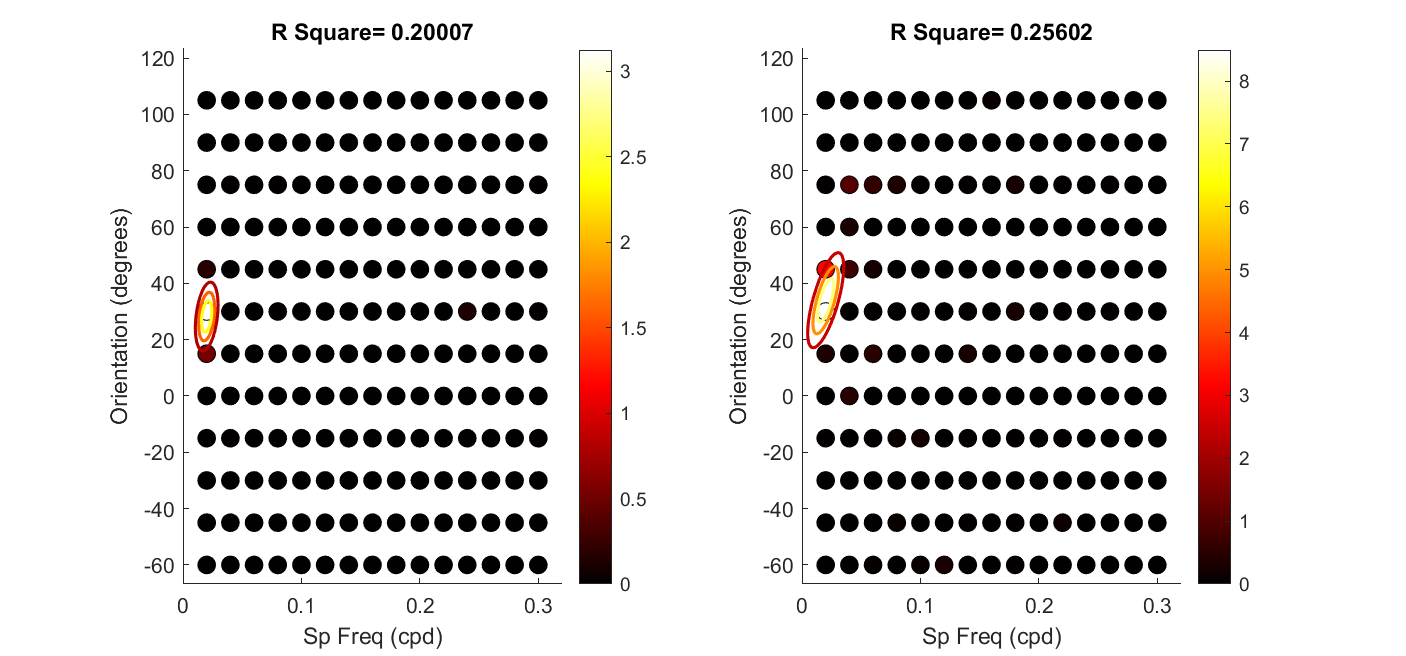

Supplement: Source data 1. — Tuning curves for all neurons scored as significantly tuned and tracked, for each of the three conditions: control, dark exposure (DE), and light reintroduction (LRx). [file elife-80361-data1.zip › SourceData1/b1_b2/2473_1R_cellPairID_2.png]

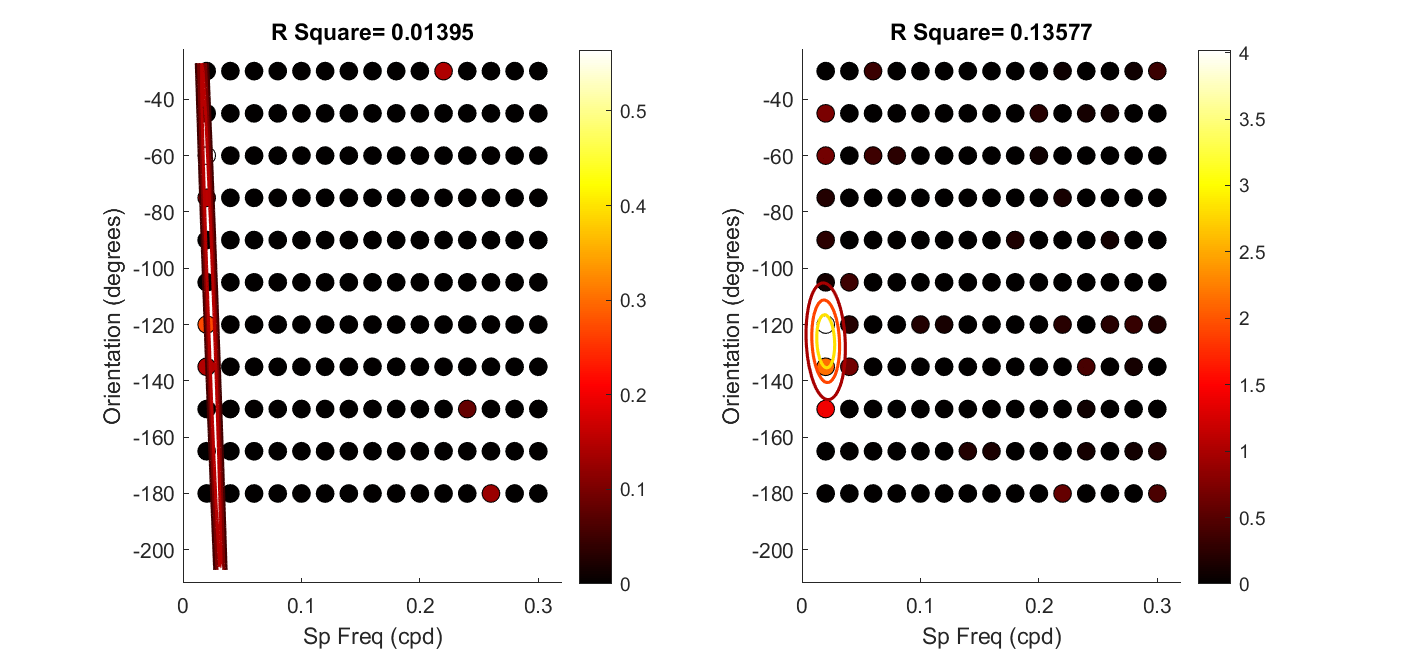

Supplement: Source data 1. — Tuning curves for all neurons scored as significantly tuned and tracked, for each of the three conditions: control, dark exposure (DE), and light reintroduction (LRx). [file elife-80361-data1.zip › SourceData1/b1_b2/2473_1R_cellPairID_20.png]

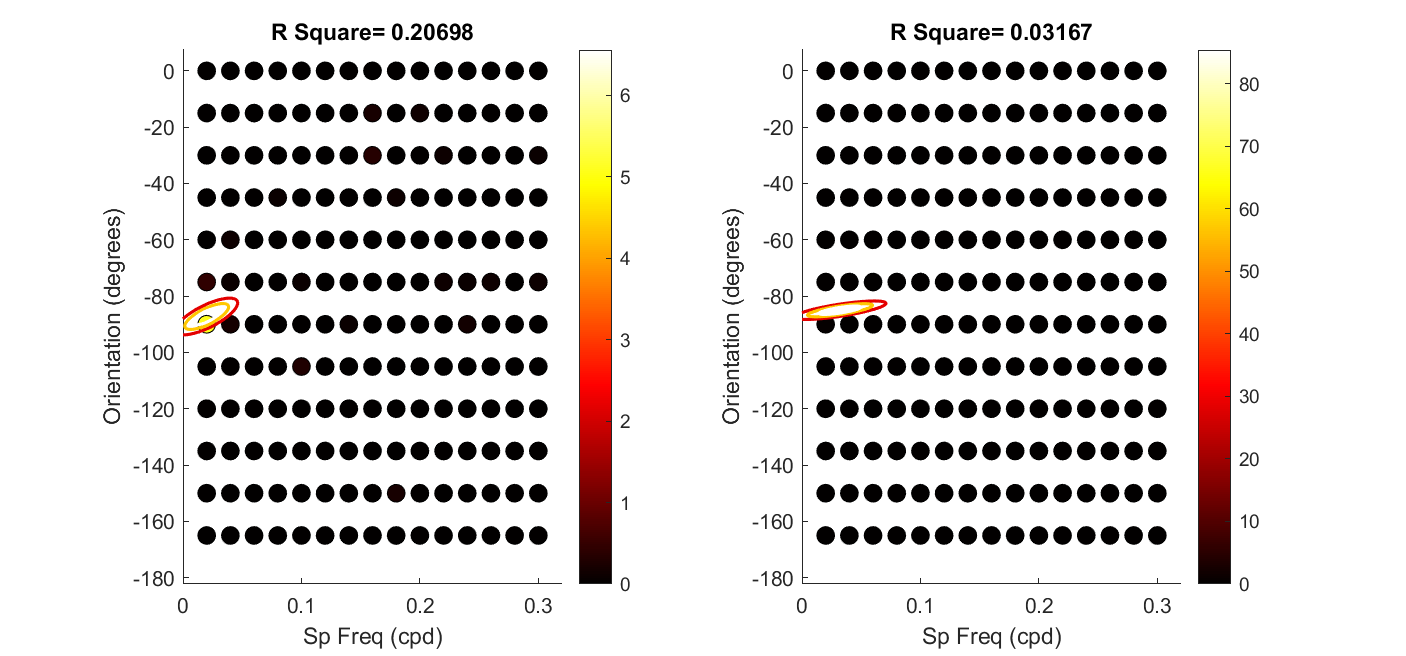

Supplement: Source data 1. — Tuning curves for all neurons scored as significantly tuned and tracked, for each of the three conditions: control, dark exposure (DE), and light reintroduction (LRx). [file elife-80361-data1.zip › SourceData1/b1_b2/2473_1R_cellPairID_21.png]

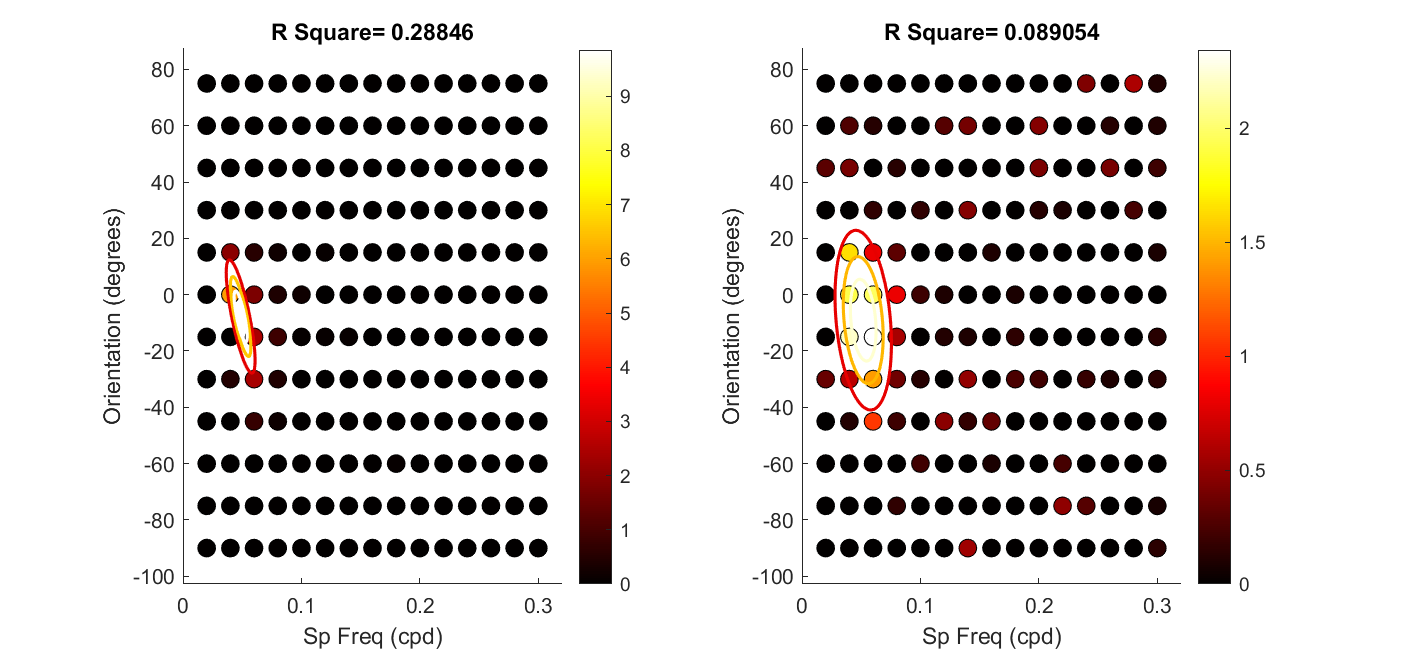

Supplement: Source data 1. — Tuning curves for all neurons scored as significantly tuned and tracked, for each of the three conditions: control, dark exposure (DE), and light reintroduction (LRx). [file elife-80361-data1.zip › SourceData1/b1_b2/2473_1R_cellPairID_22.png]

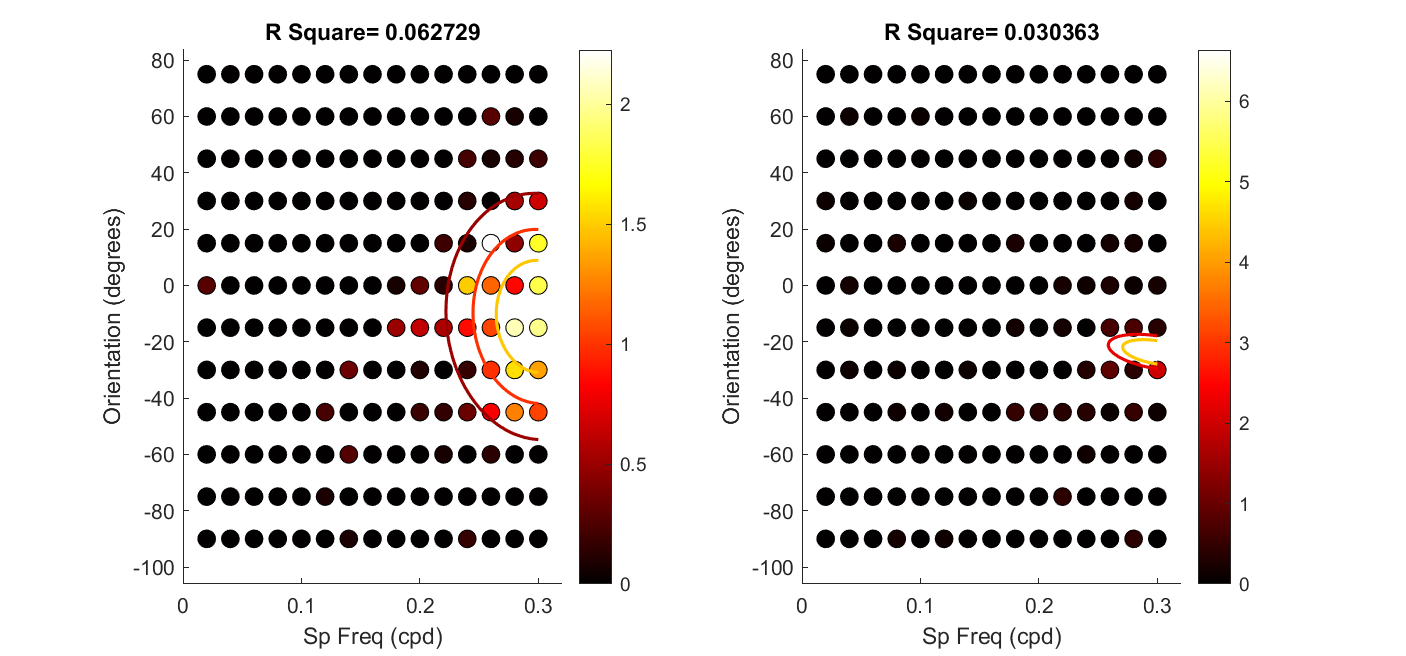

Supplement: Source data 1. — Tuning curves for all neurons scored as significantly tuned and tracked, for each of the three conditions: control, dark exposure (DE), and light reintroduction (LRx). [file elife-80361-data1.zip › SourceData1/b1_b2/2473_1R_cellPairID_23.png]

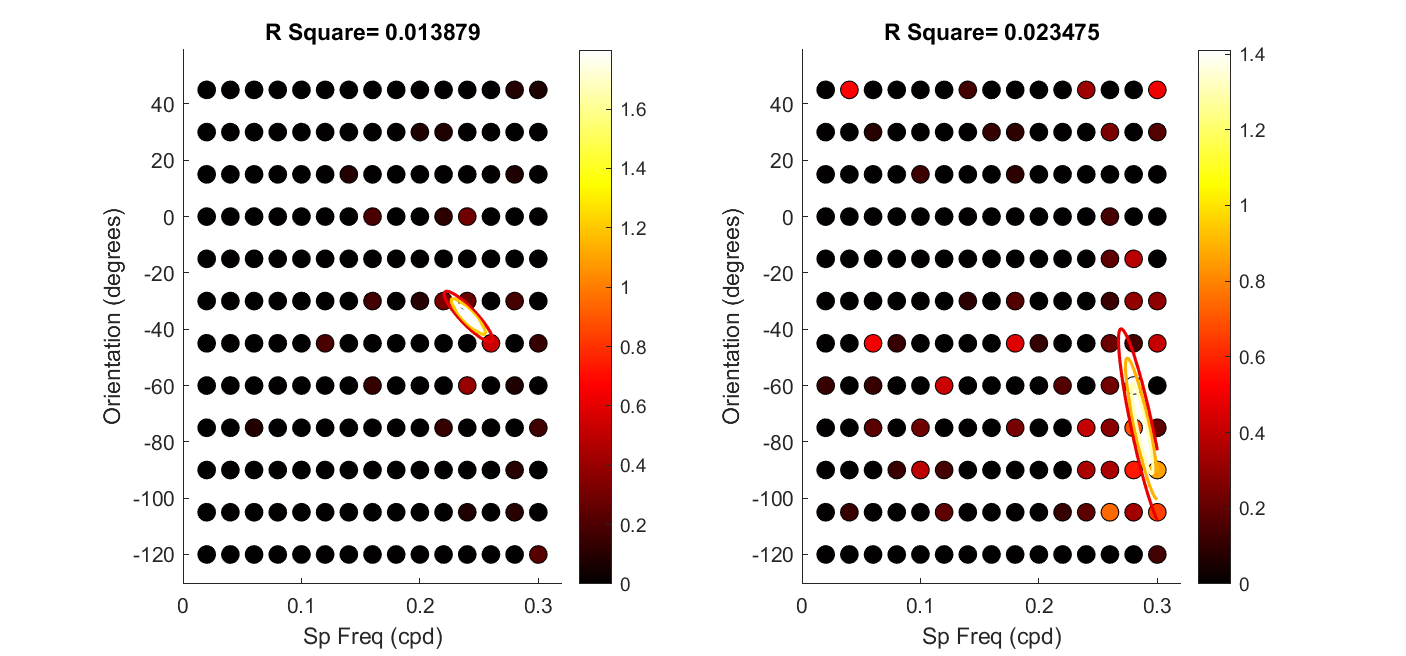

Supplement: Source data 1. — Tuning curves for all neurons scored as significantly tuned and tracked, for each of the three conditions: control, dark exposure (DE), and light reintroduction (LRx). [file elife-80361-data1.zip › SourceData1/b1_b2/2473_1R_cellPairID_24.png]

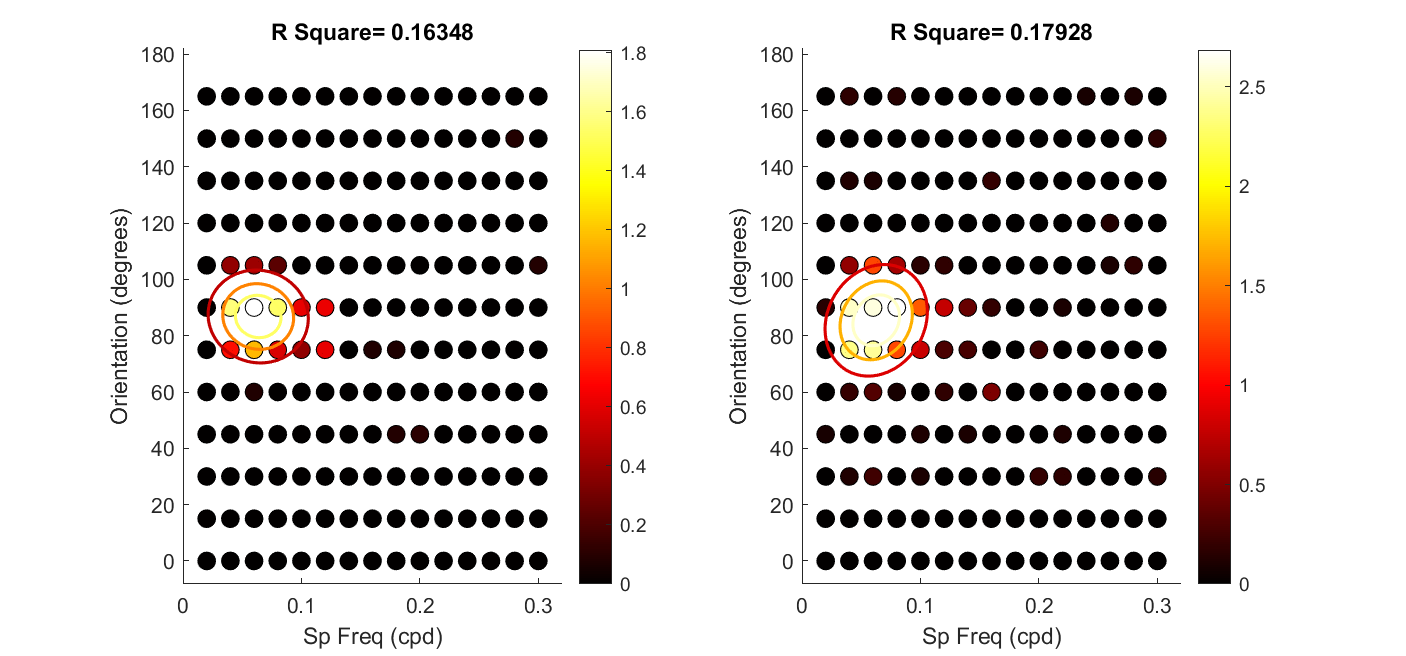

Supplement: Source data 1. — Tuning curves for all neurons scored as significantly tuned and tracked, for each of the three conditions: control, dark exposure (DE), and light reintroduction (LRx). [file elife-80361-data1.zip › SourceData1/b1_b2/2473_1R_cellPairID_25.png]

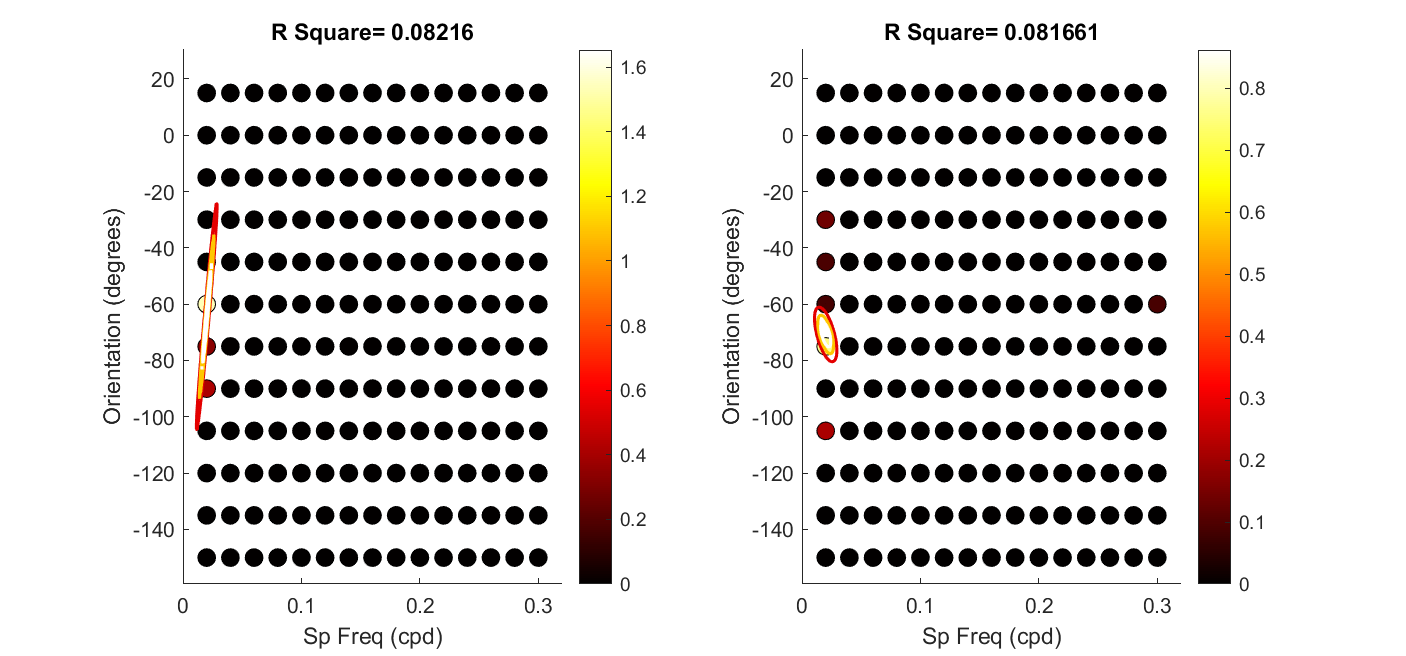

Supplement: Source data 1. — Tuning curves for all neurons scored as significantly tuned and tracked, for each of the three conditions: control, dark exposure (DE), and light reintroduction (LRx). [file elife-80361-data1.zip › SourceData1/b1_b2/2473_1R_cellPairID_26.png]

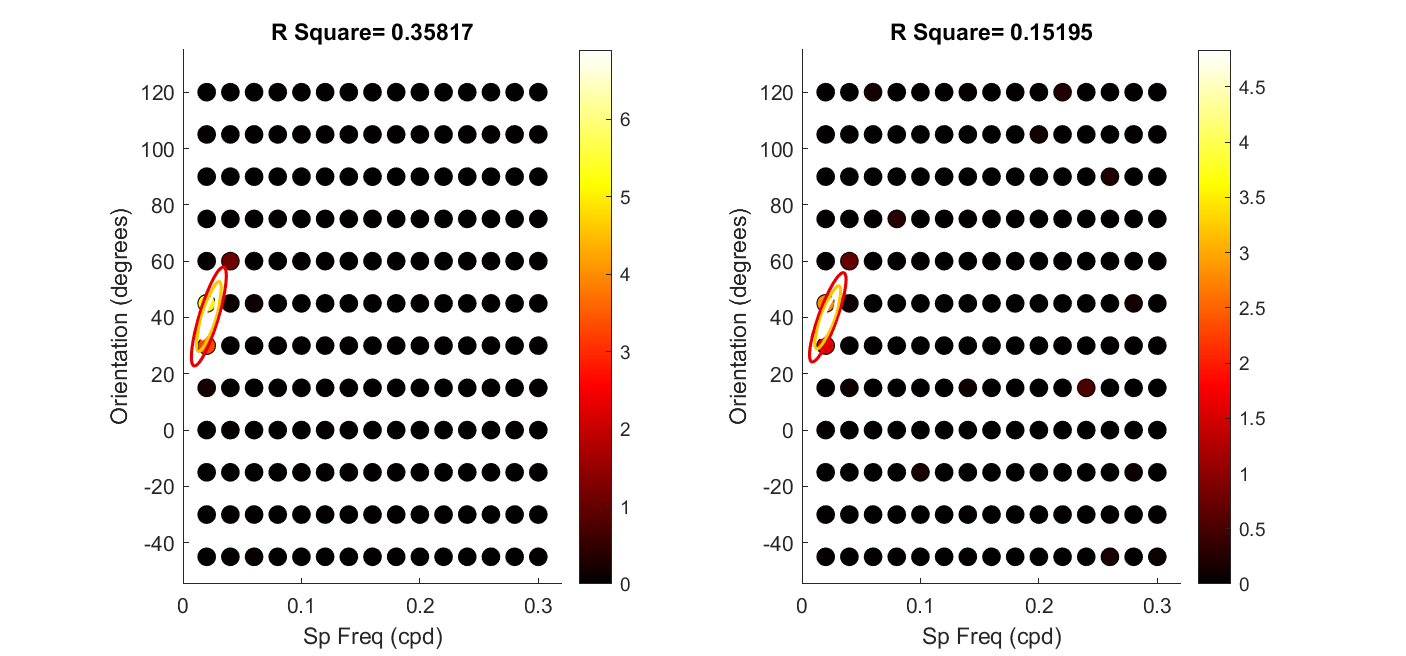

Supplement: Source data 1. — Tuning curves for all neurons scored as significantly tuned and tracked, for each of the three conditions: control, dark exposure (DE), and light reintroduction (LRx). [file elife-80361-data1.zip › SourceData1/b1_b2/2473_1R_cellPairID_27.png]

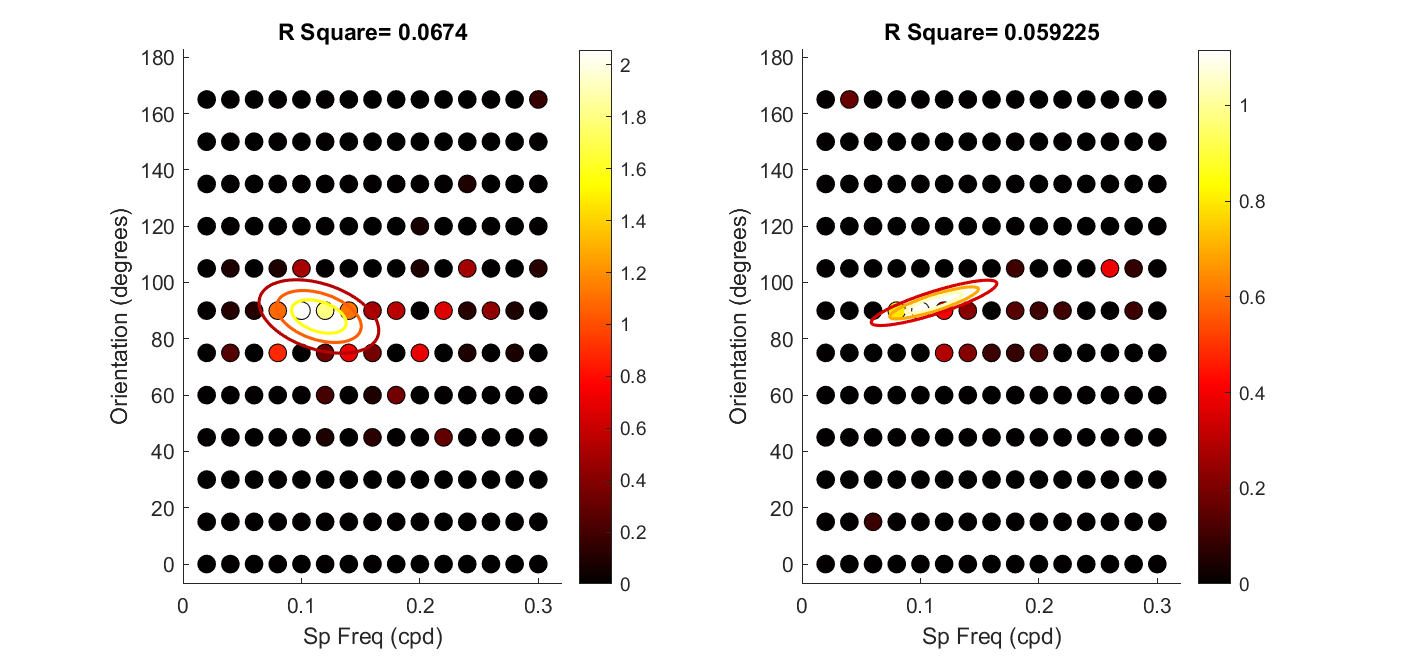

Supplement: Source data 1. — Tuning curves for all neurons scored as significantly tuned and tracked, for each of the three conditions: control, dark exposure (DE), and light reintroduction (LRx). [file elife-80361-data1.zip › SourceData1/b1_b2/2473_1R_cellPairID_28.png]

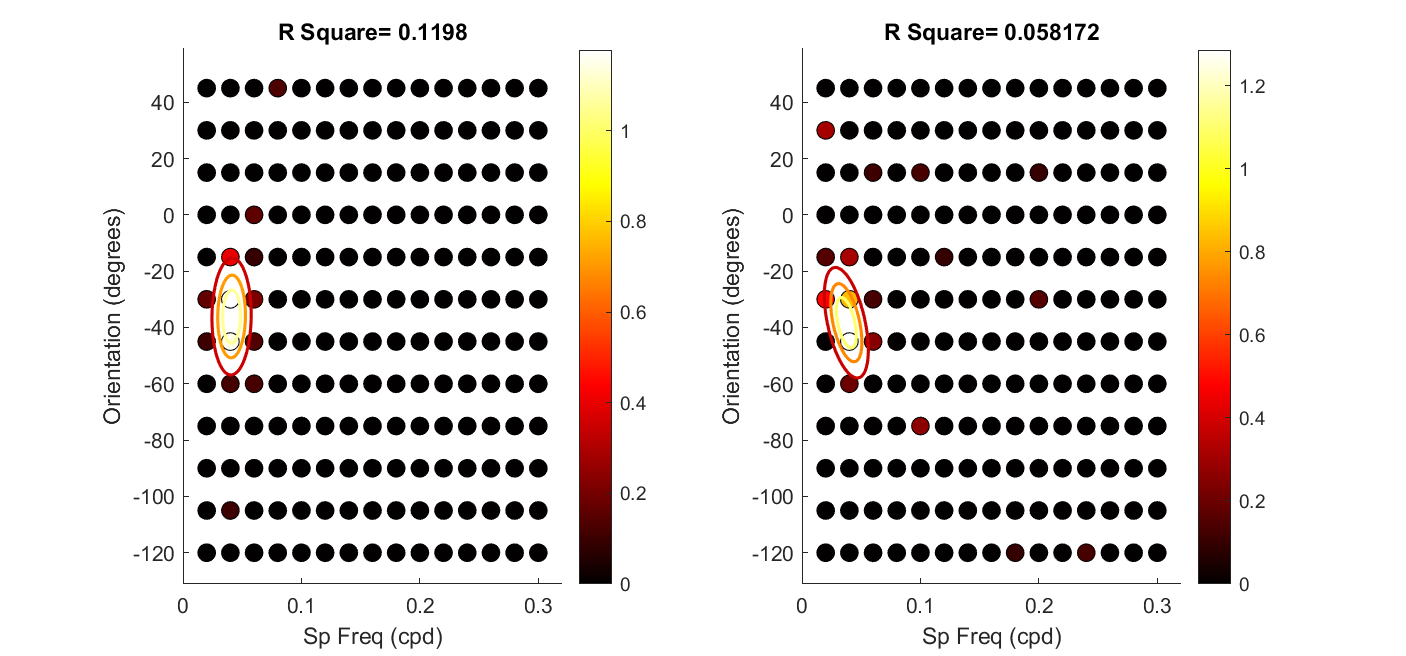

Supplement: Source data 1. — Tuning curves for all neurons scored as significantly tuned and tracked, for each of the three conditions: control, dark exposure (DE), and light reintroduction (LRx). [file elife-80361-data1.zip › SourceData1/b1_b2/2473_1R_cellPairID_29.png]

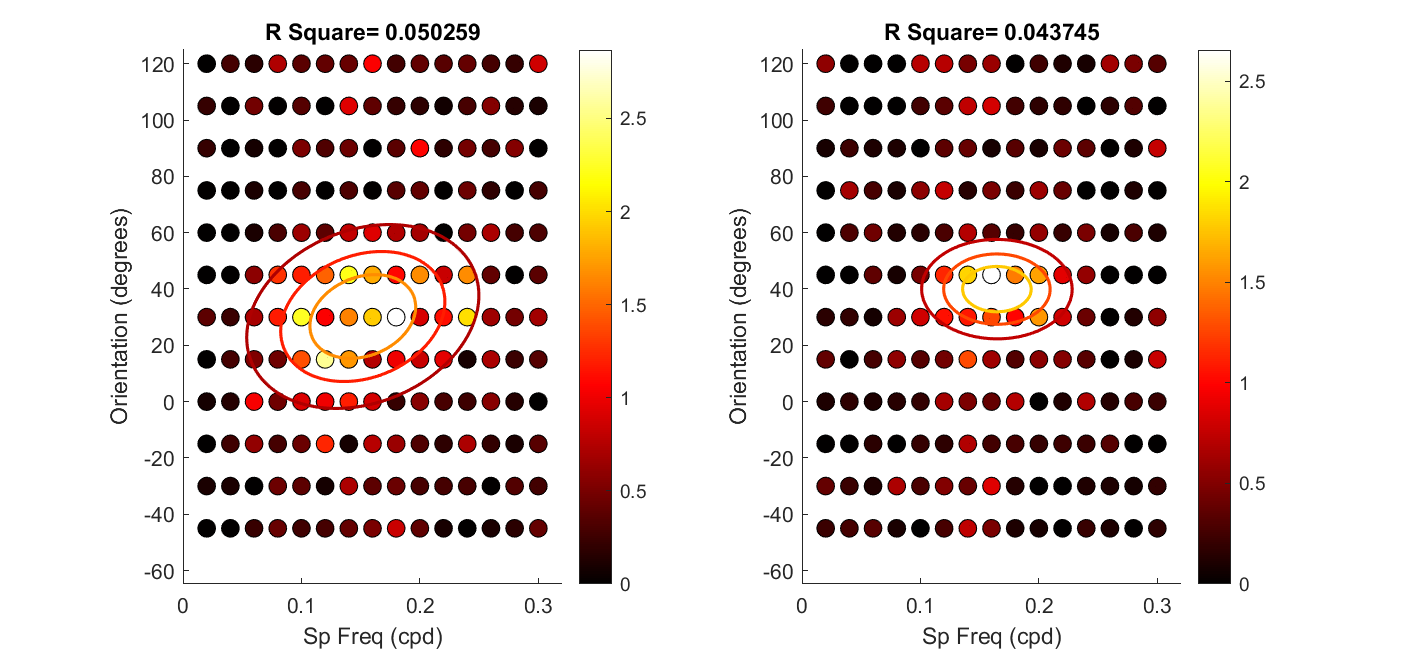

Supplement: Source data 1. — Tuning curves for all neurons scored as significantly tuned and tracked, for each of the three conditions: control, dark exposure (DE), and light reintroduction (LRx). [file elife-80361-data1.zip › SourceData1/b1_b2/2473_1R_cellPairID_3.png]

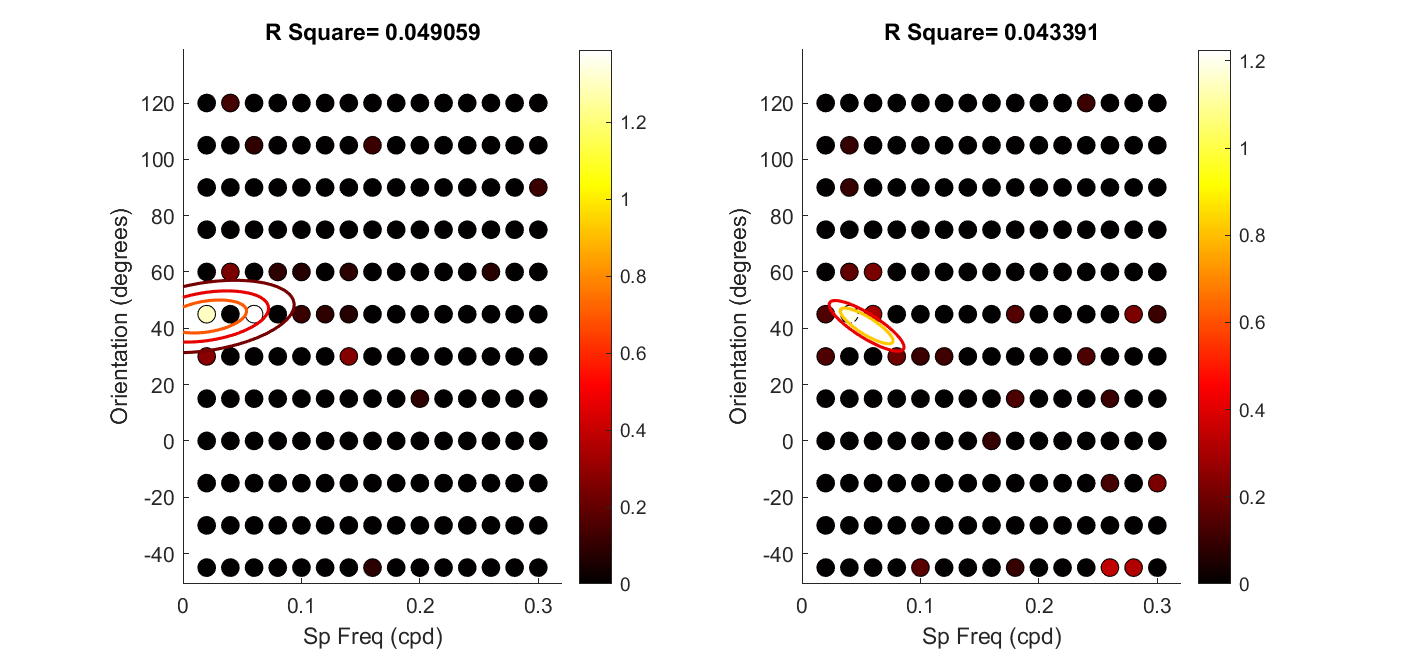

Supplement: Source data 1. — Tuning curves for all neurons scored as significantly tuned and tracked, for each of the three conditions: control, dark exposure (DE), and light reintroduction (LRx). [file elife-80361-data1.zip › SourceData1/b1_b2/2473_1R_cellPairID_30.png]

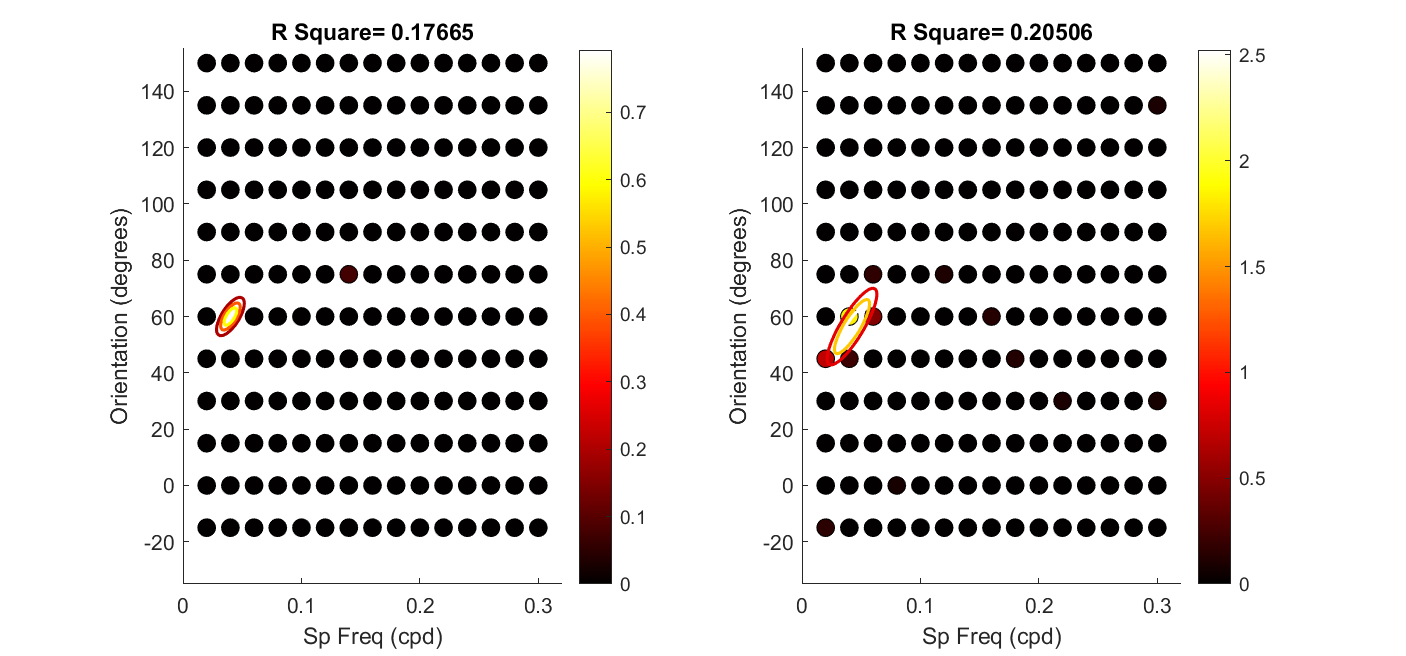

Supplement: Source data 1. — Tuning curves for all neurons scored as significantly tuned and tracked, for each of the three conditions: control, dark exposure (DE), and light reintroduction (LRx). [file elife-80361-data1.zip › SourceData1/b1_b2/2473_1R_cellPairID_31.png]

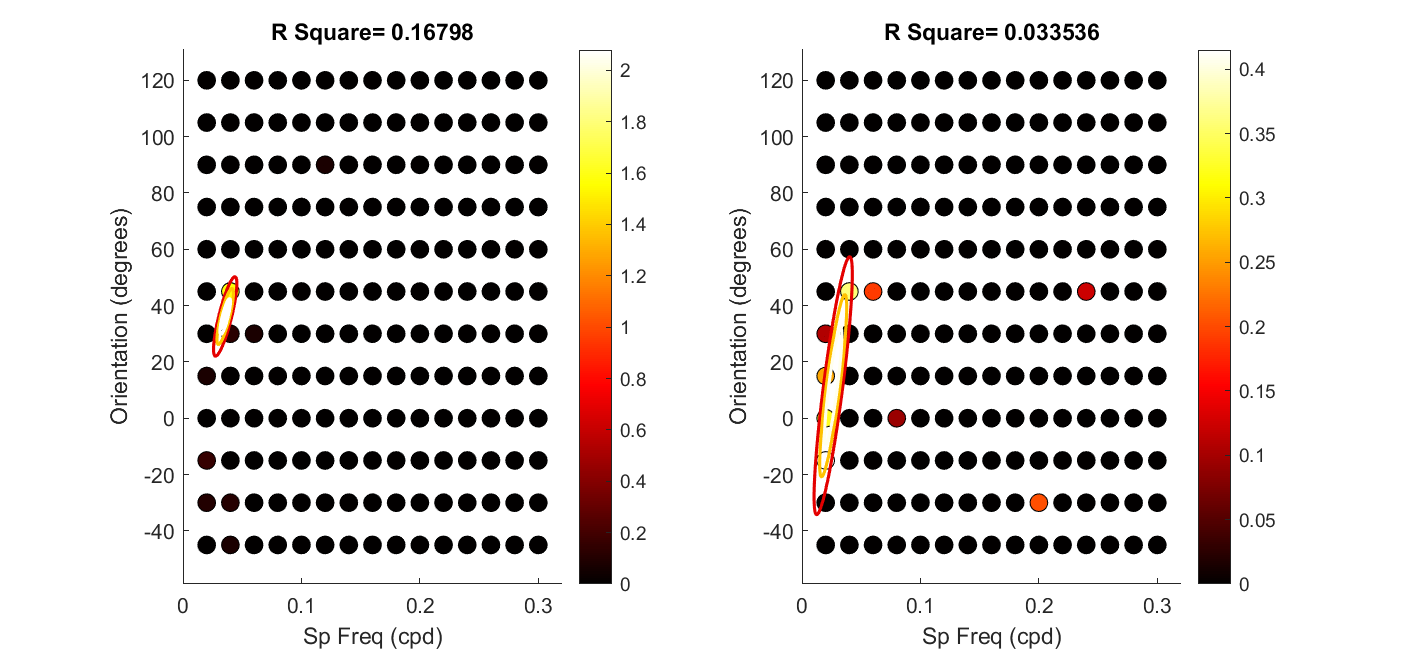

Supplement: Source data 1. — Tuning curves for all neurons scored as significantly tuned and tracked, for each of the three conditions: control, dark exposure (DE), and light reintroduction (LRx). [file elife-80361-data1.zip › SourceData1/b1_b2/2473_1R_cellPairID_32.png]

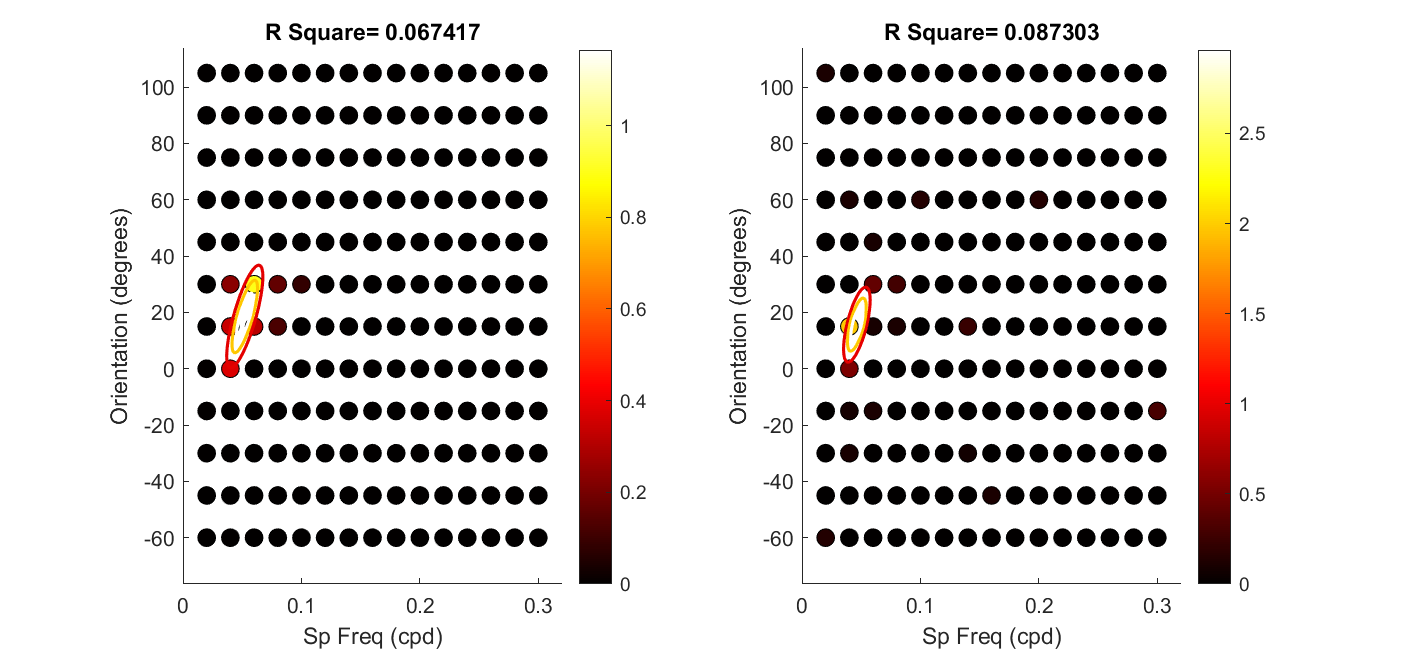

Supplement: Source data 1. — Tuning curves for all neurons scored as significantly tuned and tracked, for each of the three conditions: control, dark exposure (DE), and light reintroduction (LRx). [file elife-80361-data1.zip › SourceData1/b1_b2/2473_1R_cellPairID_33.png]
